# A Phase I Clinical Study of a Live Attenuated *Bordetella pertussis* Vaccine - BPZE1; A Single Centre, Double-Blind, Placebo-Controlled, Dose-Escalating Study of BPZE1 Given Intranasally to Healthy Adult Male Volunteers

**DOI:** 10.1371/journal.pone.0083449

**Published:** 2014-01-08

**Authors:** Rigmor Thorstensson, Birger Trollfors, Nabil Al-Tawil, Maja Jahnmatz, Jakob Bergström, Margaretha Ljungman, Anna Törner, Lena Wehlin, Annie Van Broekhoven, Fons Bosman, Anne-Sophie Debrie, Nathalie Mielcarek, Camille Locht

**Affiliations:** 1 Swedish Institute for Communicable Disease Control, Solna, Sweden; 2 Karolinska Trial Alliance, Karolinska University Hospital, Stockholm, Sweden; 3 Department of Microbiology, Tumor and Cell Biology, Karolinska Institutet, Stockholm, Sweden; 4 Q-Biologicals, BioIncubator, Zwijnaarde, Belgium; 5 Inserm, Lille, France; 6 National Center for Scientific Research, Lille, France; 7 Université Lille-Nord de France, Lille, France; 8 Center for Infection and Immunity of Lille, Institut Pasteur de Lille, Lille, France; National Institute for Public Health and the Environment, The Netherlands

## Abstract

**Background:**

Acellular pertussis vaccines do not control pertussis. A new approach to offer protection to infants is necessary. BPZE1, a genetically modified *Bordetella pertussis* strain, was developed as a live attenuated nasal pertussis vaccine by genetically eliminating or detoxifying 3 toxins.

**Methods:**

We performed a double-blind, placebo-controlled, dose-escalating study of BPZE1 given intranasally for the first time to human volunteers, the first trial of a live attenuated bacterial vaccine specifically designed for the respiratory tract. 12 subjects per dose group received 10^3^, 10^5^ or 10^7^ colony-forming units as droplets with half of the dose in each nostril. 12 controls received the diluent. Local and systemic safety and immune responses were assessed during 6 months, and nasopharyngeal colonization with BPZE1 was determined with repeated cultures during the first 4 weeks after vaccination.

**Results:**

Colonization was seen in one subject in the low dose, one in the medium dose and five in the high dose group. Significant increases in immune responses against pertussis antigens were seen in all colonized subjects. There was one serious adverse event not related to the vaccine. Other adverse events were trivial and occurred with similar frequency in the placebo and vaccine groups.

**Conclusions:**

BPZE1 is safe in healthy adults and able to transiently colonize the nasopharynx. It induces immune responses in all colonized individuals. BPZE1 can thus undergo further clinical development, including dose optimization and trials in younger age groups.

**Trial Registration:**

ClinicalTrials.gov NCT01188512

## Introduction

Pertussis is today the most prevalent vaccine-preventable disease in the developed world [1]. Two types of pertussis vaccines are currently available, the first-generation whole-cell vaccines and the more recent acellular vaccines. Thanks to a substantially improved safety profile and high efficacy [2] acellular vaccines have now replaced whole-cell vaccines in many parts of the world. However, none of these vaccines have resulted in satisfactory global pertussis control, despite a global vaccine coverage of 85% through the Extended Program on Immunization [3]. In addition, recent evidence has indicated that immunity induced by acellular pertussis vaccines wanes faster than anticipated [4], [5], which may have participated in the recent incidence increase in industrialized countries. In the US record-high numbers of cases are predicted for 2012, not seen over the last 60 years [6], illustrating the shortcomings of current vaccination strategies.

The disease is most severe and fatal in infants too young to be protected by the current vaccination programs [7]. Several strategies are being explored to protect this most vulnerable age group [8], including maternal immunization, universal lifespan vaccination to limit *Bordetella pertussis* circulation, and a cocooning strategy of vaccinating people in close contact with infants to prevent transmission to this group. However, they all suffer from their inherent limitations [9]. As an alternative, early vaccination, possibly at birth, would be desirable, but the functional immaturity of the neonatal immune system [10] may hamper this approach using the current pertussis vaccines.

However, in contrast to immunization, natural infection by *B. pertussis* induces strong immune responses, even in very young infants [11], suggesting that the best way to induce early protection in infants is through infection. A genetically modified *B. pertussis* strain has therefore been constructed as a vaccine candidate to be administered by the nasal route, in order to mimic natural infection without inducing disease. In this strain, named BPZE1, dermonecrotic toxin and tracheal cytotoxin have been genetically removed, whereas pertussis toxin (PT) has been genetically detoxified by two independent mutations, each deactivating the toxic activity of PT, without affecting the immunogenic properties [12]. In pre-clinical models, BPZE1 was shown to be safe, including in severely immuno-compromised animals [13], and able to induce fast, strong and long-lasting protective immunity after a single nasal administration, even in newborn animals [14], [15]. The excellent safety profile of BPZE1 and its demonstrated genetic stability [16] have allowed this strain to be declassified from the safety level 2 to level 1 in several countries, a pre-requisite for its clinical development.

Here, we tested BPZE1 for the first time in man in a placebo-controlled, double-blind, dose-escalation trial to provide proof of concept of its safety, its ability to colonize and its immunogenicity when given nasally to healthy young adult male volunteers in a single dose. This is the first time that a live attenuated bacterial vaccine specifically designed for the nasal route has ever been tested in humans.

## Methods

The protocol for this trial and supporting CONSORT checklist are available as supporting information; see Protocol S1 and Checklist S1.

### Ethical Conduct of the Study

The study was conducted in accordance with the protocol, ICH Good Clinical Practices standards, Declaration of Helsinki and applicable regulatory requirements as well as any European and Swedish applicable laws and regulations relating. The trial is registered with ClinicalTrials.gov, NCT01188512. The Swedish Medical Product Agency (MPA) and the regional ethical review board in Stockholm approved the study and the amendments. Since this was a first-in-man study of a genetically modified microorganism it was a requirement from the MPA, that only males should be enrolled in the study. The volunteers signed the informed consent form after receiving written and oral information. Information was given before and during visit 1, so the subject had sufficient time to consider participation, which started on visit 2.

The study was performed at the phase I unit, Karolinska Trial Alliance, Karolinska University Hospital, Stockholm, Sweden (http://www.karolinskatrialalliance.se/en).

### Study Subjects

Healthy adult male volunteers aged 19–31 years, born in Sweden between 1979 and 1991, when no pertussis vaccination was performed in Sweden, were included step-wise. A volunteer was eligible for inclusion in the study if he had not experienced clinical pertussis during the preceding 10 years and had serum anti-PT IgG ≤20 IU/ml. Persons in frequent contacts with children less than one year of age were not included. Other inclusion and exclusion criteria are listed in Method S1 and all scheduled visits are shown in Table S1. The first visit of the first study subject was on August 10^th^ 2010 and the last visit of the last subject was on June 21^st^ 2011.

Baseline data of the volunteers are shown in Table 1.

**Table 1:** Demografic and baseline data per dose group.

Variable	Placebo *(N = 12)*	Low dose *(N = 12)*	Medium dose *(N = 12)*	High dose *(N = 12)*
Age (year)	22 (22–28)	23 (21–26)	24 (22–27)	22 (22–25)
Height (cm)	187 (184–190)	182 (180–186)	182 (180–185)	182 (176–186)
Weight (kg)	86 (78–94)	74 (64–82)	82 (74–88)	80 (74–86)
Systolic blood pressure, mm Hg	130 (128–132)	118 (114–124)	125 (118–130)	128 (125–136)
Diastolic blood pressure, mm Hg	72 (69–74)	69 (66–70)	67 (64–72)	72 (68–76)
Heart rate, beats/min	62 (58–66)	59 (50–64)	64 (59–66)	60 (53–68)
Respiratory rate, breaths/min	16 (13–17)	16 (16–17)	16 (14–16)	16 (15–16)
Oral temperature, °C	36.6 (36.3–36.7)	36.5 (36.3–36.6)	36.6 (36.4–36.7)	36.5 (36.0–36.7)

Results are presented as median with interquartile ranges within parenthesis.

### Randomization and Vaccination

**Figure 1:**
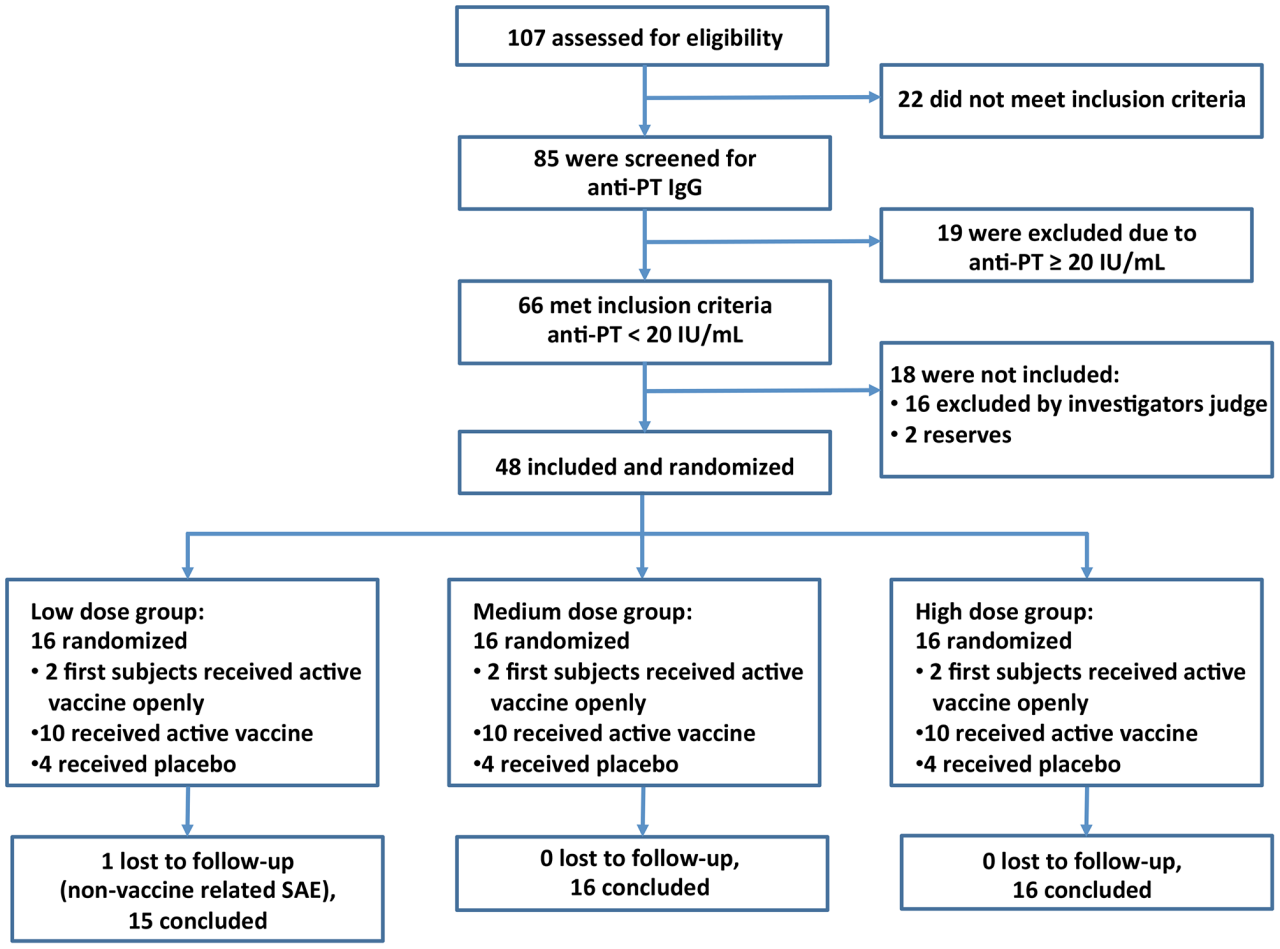
CONSORT Flow Diagram. Number of subjects assessed for eligibility, enrolled and randomized to study vaccine or placebo. Subjects were included in a step-wise fashion with 16 subjects in each group. Interim safety analysis was performed before vaccination of the next, higher dose group.

Sixteen individuals were included in each of the three dose groups (Figure 1), with 12 volunteers/group receiving BPZE1 [Group 1 (low dose), 10^3^ colony forming units (cfu); Group 2 (medium dose), 10^5^ cfu; Group 3 (high dose), 10^7^ cfu] and 4 volunteers/group receiving the diluent as placebo. Thus the 12 placebo recipients were evenly distributed throughout the whole study period. The administration of vaccine or diluent alone was performed in a double-blind fashion with administration of coded vials with exception for the two first study subjects in each dosage group, who received active substance (unblinded) as required by the Swedish MPA.

A liquid formulation of three different BPZE1 batches (low, medium and high dose) in phosphate-buffered saline containing 5% saccharose (manufactured, randomized and sequentially numbered by Innogenetics, Belgium) was used. Vaccine or placebo was given as single administration by nasal application of 0.1 ml in each nostril. The dose of the active ingredient or placebo was administered within 2 hours after thawing. The volunteers were vaccinated between September 9^th^, 2010 and January 20^th^, 2011.

### Safety Follow-up

The volunteers provided written informed consent and were screened 2–6 weeks before vaccination. The Toxicity Grading Scale for Healthy Adult and Adolescent Volunteers Enrolled in Preventive Vaccine Trials [17] was followed for definition of adverse events (AE) and their intensity. The principal investigator or delegate determined the relationship of an AE to the study vaccine as follows: None, Possible, Probable and Definite. To evaluate immediate AE, subjects were kept at the investigator site under surveillance for at least 6 hours following vaccination. Any events occurring during this time period were to be reported by the investigator or delegate. Thereafter, the volunteers visited the clinic at days 4±1, 7±1, 11±1, 14±1 and 28 (−1; +7) and 5–6 months after vaccination for physical examination, sample collection and solicited questions concerning general and local AE and unsolicited AE. In addition, from the day of vaccination to day 14 after vaccination the subjects recorded AE in a diary, which was returned on day 28 to the investigator. Severe AE (SAE) were reported at all visits, and the subjects were instructed to immediately contact the investigator in case of any SAE. The relation of the adverse events to the vaccine were determined jointly and unanimously by the principal investigator, who was also the clinical investigator (author NA), and the author responsible for safety issues (BT). All serious adverse events were discussed with the sponsor and the Independent Data Monitoring Committee (IDMC).

Group 1 was followed for at least 7 weeks before group 2 was vaccinated. Group 2 was followed for at least 9 weeks before group 3 was vaccinated. Interim safety meetings were held with IDMC and the sponsor before administering the next dose.

### Sample Collection

Venous blood samples were collected at screening 2–6 weeks prior to vaccination, on the day of vaccination and at 1, 2 and 4 weeks and 5–6 months after vaccination.

Nasopharyngeal aspirates were collected to determine colonization by BPZE1 of the nasopharyngeal mucosa 4, 7, 11, 14 and 28 days and, if necessary, 45 days after vaccination. The aspirates were grown on charcoal agar plates (Oxoid, CM119, with 10% horse blood and 40 mg/L cephalexine). The plates were read daily for up to 7 days. The presence of *B. pertussis* was confirmed by Gram stain and Catalase and Oxidase test. For further details see Supporting information Method S2.

Serum IgG antibodies against PT, filamentous hemagglutinin (FHA), pertactin and fimbriae types 2/3 were analyzed by standardized ELISA as described in Supporting information Method S3.

Haemoglobin and cell blood count (total and differential white blood cells, red blood cells, and platelets) were analyzed on a Sysmex XE-5000 instrument.

### Data and sample deposition

Case report forms, patient journals and raw data collected at the clinic are archived at the Karolinska Trial Alliance, Karolinska University Hospital. Raw data from the laboratory testing and all other documentation from the trial are archived at the Swedish Institute for Communicable Disease Control for at least 15 years. Samples collected for isolation and immunogenicity testing are stored in the biobank at the Swedish Institute for Communicable Disease Control.

### Statistical Analysis

The difference between the vaccine dose groups (low-, medium-, and high dose and placebo) in the number of local and systemic solicited AE, and the proportion of subjects colonized with BPZE1 was evaluated using Fisheŕs exact test for contingency tables.

Due to the limited number in each placebo group (n = 4) the power of any test between the three placebo groups would be very low. Therefore only comparisons were performed between the dosage groups and the whole placebo group (n = 12).

Due to non-normally distributed data for the specific antibody levels, Friedman’s one-way analysis of variance for repeated measures was used to compare the overall differences in serum IgG distribution over time. In case of a significant overall test, pairwise comparisons with day 0 were performed between time-points within respective dose group.

For the same reason, Kruskal-Wallis one-way analysis of variance was used to compare the overall differences in IgG levels between dose groups. The dose groups were compared at all time points in separate analyses. Pairwise comparisons between dose groups were performed if the overall test showed a significant test result.

All hypothesis tests performed were two-sided and p<0.05 was considered significant.

All statistical analyses were done using the software R (R Core Team (2012). R: A language and environment for statistical computing. R Foundation for Statistical Computing, Vienna, Austria.) [18].

## Results

There were no differences in baseline data between the volunteers assigned to vaccine or placebo except for the volunteers in the low dose group, who had lower systolic blood pressure than other groups, but still within normal limits (Table 1). In total 107 subjects were recruited of which 48 were vaccinated. All vaccinated volunteers were evaluable for safety and immunogenicity during the first month after vaccination, and only one was lost to follow up after 5–6 months (Figure 1).

### Adverse Events

There was one SAE. The first subject vaccinated openly with the low dose died 7 weeks after vaccination. The SAE was reported to the sponsor and the sponsor notified the Swedish MPA, the European Medical Agency Eudravigilance database and the Ethics Committee. After careful assessment, the death was judged by the IDMC as not vaccine-related. Out of consideration for the deceased and his family it was decided not to give more information about the death.

Immediate AE within 6 hours of vaccination were all trivial and short-lasting with no differences between the placebo and different dosage groups.

Local and systemic solicited AE, which were noted in the diary and confirmed with interviews at subsequent visits, were all mild to moderate. The most common were rhinorrhoea, sneezing and cough. There were no differences between placebo and any of the treatment groups (Tables 2, 3, S2 and S3) or between colonized and non-colonized subjects (Table S4). Ten volunteers experienced cough during the first week after vaccination and five during the second week. The cough episodes were evenly distributed among the placebo and treatment groups, short-lasting and without similarity with whooping cough.

**Table 2:** Number of local solicited adverse events during week 1 and 2 for each dose group (n = 12 per group).

		Week 1				Week 2			
Adverse event	Intensity	Placebo	Low dose	Medium dose	High dose	Placebo	Low dose	Medium dose	High dose
**Cough**	None	10	8	9	11	11	9	10	12
	Mild	1	3	3	1	0	2	2	0
	Moderate	0	1	0	0	1	1	0	0
	High	1	0	0	0	0	0	0	0
	All intensities	2	4	3	1	1	3	2	0
**Nasal congestion**	None	6	7	10	7	11	8	9	10
	Mild	4	4	2	5	1	3	3	2
	Moderate	2	1	0	0	0	1	0	0
	High	0	0	0	0	0	0	0	0
	All intensities	6	5	2	5	1	4	3	2
**Epistaxis**	None	12	10	12	11	12	12	12	12
	Mild	0	2	0	1	0	0	0	0
	Moderate	0	0	0	0	0	0	0	0
	High	0	0	0	0	0	0	0	0
	All intensities	0	2	0	1	0	0	0	0
**Rhinorrhoea**	None	5	8	9	6	10	7	11	10
	Mild	5	4	3	4	2	4	1	2
	Moderate	2	0	0	2	0	1	0	0
	High	0	0	0	0	0	0	0	0
	All intensities	7	4	3	6	2	5	1	2
**Sneezing**	None	6	11	7	8	9	10	9	11
	Mild	6	1	5	3	3	1	3	1
	Moderate	0	0	0	1	0	1	0	0
	High	0	0	0	0	0	0	0	0
	All intensities	6	1	5	4	3	2	3	1
**Ear pain**	None	12	12	12	10	12	11	12	11
	Mild	0	0	0	2	0	1	0	1
	Moderate	0	0	0	0	0	0	0	0
	High	0	0	0	0	0	0	0	0
	All intensities	0	0	0	2	0	1	0	1
**Eye pain**	None	12	12	12	11	12	12	12	12
	Mild	0	0	0	1	0	0	0	0
	Moderate	0	0	0	0	0	0	0	0
	High	0	0	0	0	0	0	0	0
	All intensities	0	0	0	1	0	0	0	0
**Dyspnoea**	None	12	11	11	12	12	12	12	12
	Mild	0	1	1	0	0	0	0	0
	Moderate	0	0	0	0	0	0	0	0
	High	0	0	0	0	0	0	0	0
	All intensities	0	1	1	0	0	0	0	0

**Table 3:** Number of systemic solicited adverse events during week 1 and 2 for each dose group (n = 12 per group).

		Week 1				Week 2			
Adverse event	Intensity	Placebo	Low dose	Medium dose	High dose	Placebo	Low dose	Medium dose	High dose
**Tiredness**	None	6	5	8	8	12	10	12	10
	Mild	5	5	1	4	0	2	0	1
	Moderate	0	1	3	0	0	0	0	1
	High	1	1	0	0	0	0	0	0
	All intensities	6	7	4	4	0	2	0	2
**Headache**	None	7	9	9	7	11	10	10	10
	Mild	3	2	3	4	1	2	2	2
	Moderate	1	1	0	1	0	0	0	0
	High	1	0	0	0	0	0	0	0
	All intensities	5	3	3	5	1	2	2	2
**Pyrexia**	None	12	12	12	12	12	10	11	12
	Mild	0	0	0	0	0	1	0	0
	Moderate	0	0	0	0	0	1	1	0
	High	0	0	0	0	0	0	0	0
	All intensities	0	0	0	0	0	2	1	0
**Other specified pain**	None	12	12	12	11	12	12	12	12
	Mild	0	0	0	1	0	0	0	0
	Moderate	0	0	0	0	0	0	0	0
	High	0	0	0	0	0	0	0	0
	All intensities	0	0	0	1	0	0	0	0

There were no abnormalities in vital signs or blood haematology in any volunteer during the study (data not shown).

All volunteers were asked unsolicited questions about AE at all visits, including the last 5–6 months after the vaccination. There was no SAE and only mild to moderate episodes of upper respiratory tract infections and short-lasting fever with no differences between groups.

### Colonization

**Figure 2:**
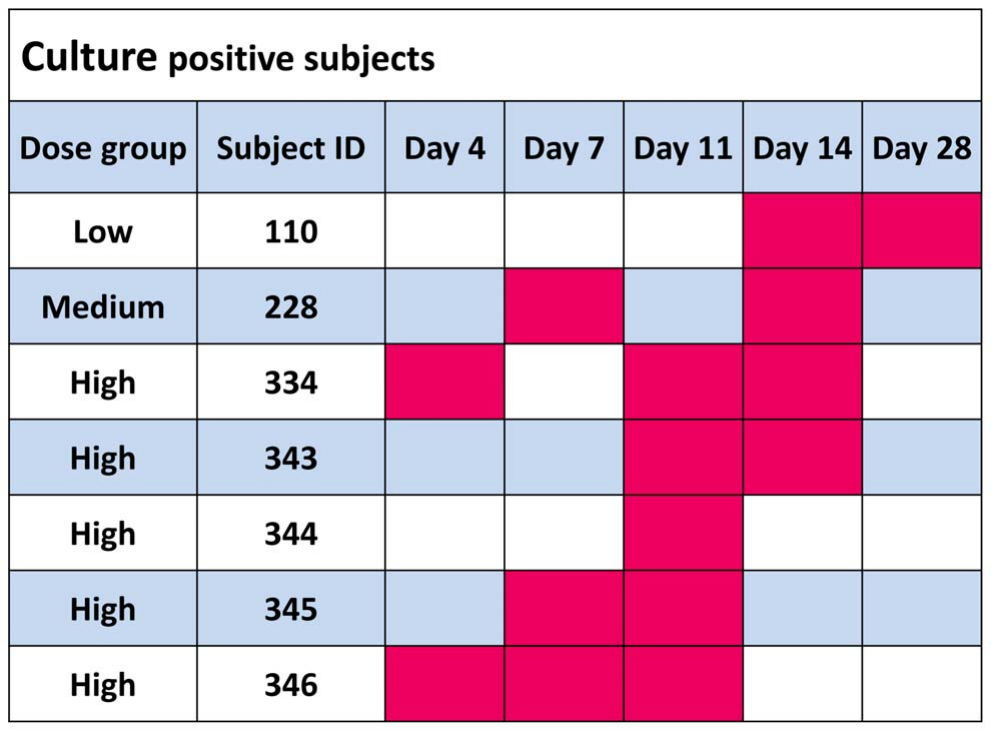
Colonization of nasopharyngeal mucosa from day 4 to day 28 after vaccination. Culture positive samples are shown in red.

BPZE1 was isolated 1–3 times from one subject in the low-dose group, one in the medium-dose group and five in the high-dose group between days 4 and 14 (Figure 2). On day 28 all were culture negative except the colonized subject in the low-dose group, who was negative on the next visit, at day 49 after vaccination. The frequency of colonization was significantly higher in the high-dose group compared to the low and medium-dose groups combined (p = 0.029).

### Immunogenicity

**Figure 3:**
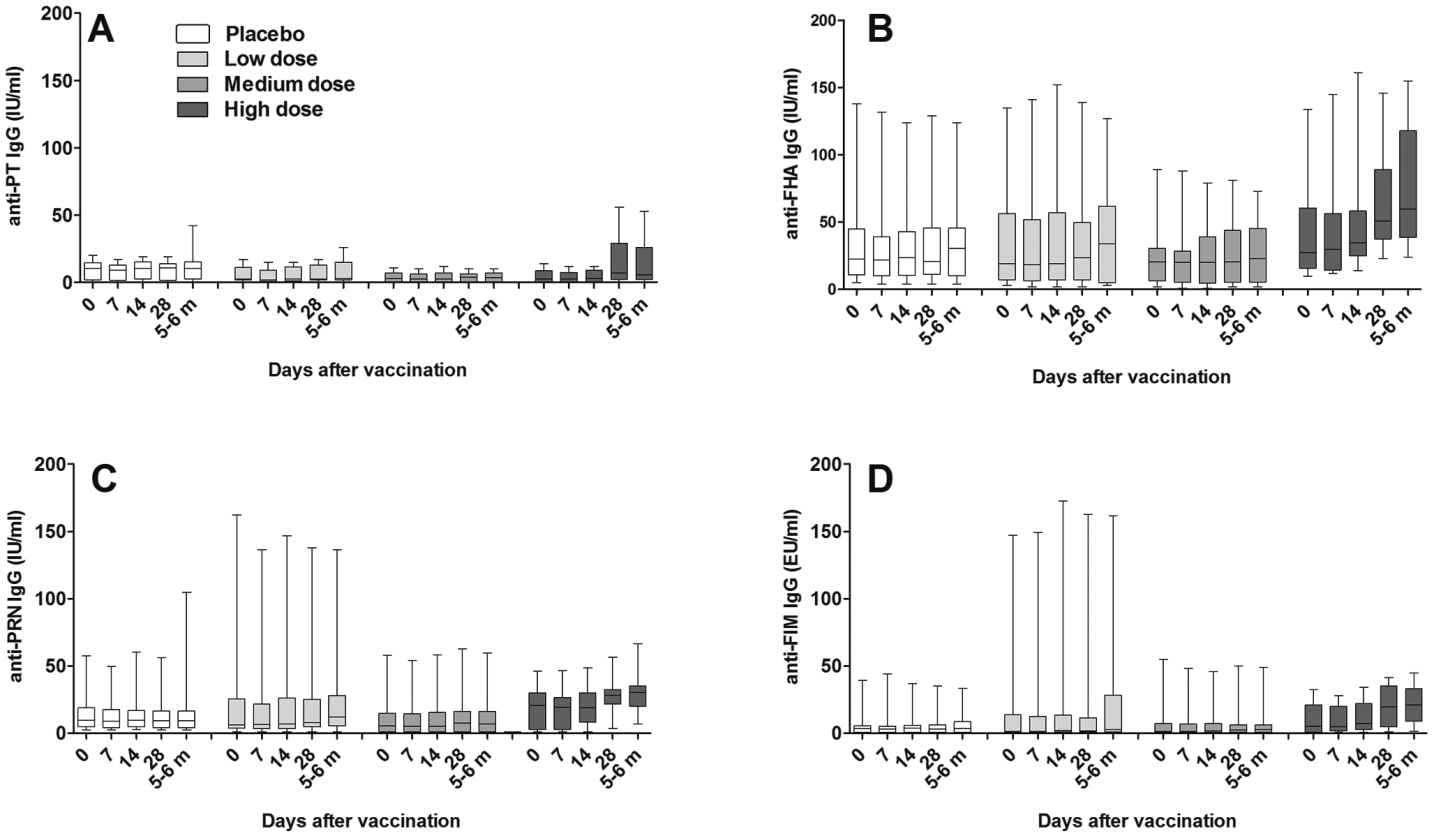
Serum IgG levels over time to *B. pertussis* antigens in respective dose group. A) anti-PT, B) anti-FHA, C) anti-PRN, and D) anti-Fim. Day 0 = Day of vaccination, 5–6 m = 5–6 months after vaccination. PT, pertussis toxin; FHA, filamentous hemagglutinin; PRN, pertactin; and Fim, fimbriae 2/3. Results are presented as boxplots with medians and interquartile ranges and whiskers for min-max range.

**Figure 4:**
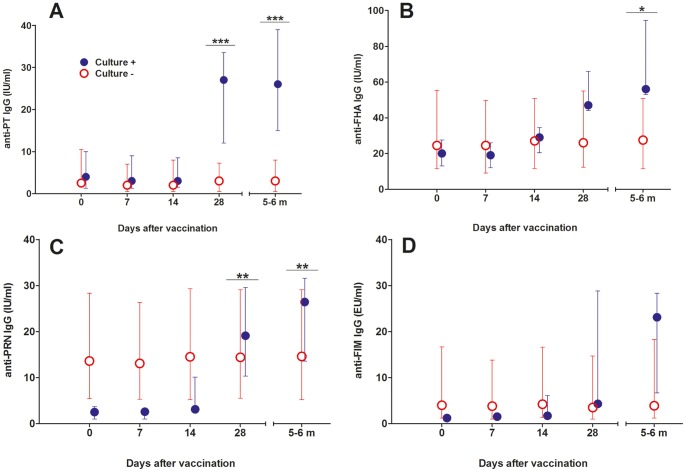
Serum IgG levels over time to *B. pertussis* antigens in the colonized compared to non-colonized subjects. A) anti-PT, B) anti-FHA, C) anti-PRN, and D) anti-Fim. PT, pertussis toxin; FHA, filamentous hemagglutinin; PRN, pertactin; and Fim, fimbriae 2/3. Significant differences were found between the two groups for anti-PT at 28 days (p = 0.001) and 5–6 months (p = 0.001); for anti-FHA at 5–6 months (p = 0.026); and for anti-PRN at 28 days (p = 0.009) and 5–6 months (p = 0.008). Results are presented as medians (circles) and interquartile ranges (error bars).

Serum antibodies against PT, FHA, pertactin and fimbriae appeared between days 14 and 28 and remained high during the 6 months follow up (Figures 3 and 4). Pairwise comparisons within the high dose group showed significant increases in IgG levels against pertactin at day 28 (p = 0.018) and after 5–6 months (p = 0.001) compared to day 0 and also for fimbriae at both day 28 (p = 0.015) and after 5–6 months (p = 0.006). Increases in anti-PT and anti-FHA IgG levels were not significant. Results between dose groups were similar. The pairwise tests between dose groups showed that anti-FHA and anti-pertactin were significantly higher in the high dose group than in the placebo, the low- and medium dose groups at day 28 and/or at 5–6 month. Anti-fimbriae IgG was only found significantly lower in the medium dose group, while no differences between dose groups were seen for anti-PT IgG (Figure 3 and Table 3).

However, when colonized subjects were compared to non-colonized volunteers pronounced differences were detected for all tested antigens. No increase in serum IgG to *B. pertussis* antigens was detected in the placebo group and among non-colonized subjects in any dose group, while pronounced increases against all four tested antigens occurred among the colonized subjects. Overall significant increases were seen in the colonized group for IgG to PT (p = 0.007), FHA (p<0.001), pertactin (p<0.001), and fimbriae (p<0.001). Pairwise comparisons between culture positive and negative subjects showed significant differences for anti-PT, anti-FHA and anti-pertactin but not for anti-fimbriae (Figure 4). Individual data for culture positive subjects are shown in Table 4.

**Table 4:** Individual immune responses to BPZE1 vaccination in culture positive volunteers.

	Serum IgG anti-PT (IU/ml)					Serum IgG anti-FHA (IU/ml)					Serum IgG anti-PRN (IU/ml)					Serum IgG anti-FIM (EU/ml)				
	Day 0	Day 7	Day 14	Day 28	5–6 Months	Day 0	Day 7	Day 14	Day 28	5–6 Months	Day 0	Day 7	Day 14	Day 28	5–6 Months	Day 0	Day 7	Day 14	Day 28	5–6 Months
**110**	4	3	3	**13**	**26**	20	19	19	30	**47**	3	3	3	**8**	**28**	1	2	2	**4**	**28**
**228**	1	1	1	**4**	**4**	31	29	42	54	**56**	1	1	2	**12**	**13**	1	1	2	3	**4**
**334**	2	2	2	**30**	**20**	13	12	22	**144**	**127**	1	1	3	**27**	**14**	1	2	**8**	**36**	**10**
**343**	14	12	10	**37**	**50**	13	12	14	**42**	**51**	1	1	1	3	**7**	1	1	1	1	2
**344**	1	1	1	**56**	**53**	10	12	**37**	**78**	**124**	3	3	**19**	**45**	**66**	1	1	1	**4**	**29**
**345**	12	12	12	**27**	**28**	31	35	32	47	**65**	6	6	7	**19**	**35**	3	3	5	**41**	**45**
**346**	8	6	7	11	10	24	23	29	46	**55**	4	4	**13**	**33**	**26**	13	14	17	22	23
***Cult.pos Median***	*4*	*3*	*3*	*27*	*26*	*20*	*19*	*29*	*47*	*56*	*3*	*3*	*3*	*19*	*26*	*1*	*2*	*2*	*4*	*23*
***Cult.neg Median***	*3*	*2*	*2*	*3*	*3*	*25*	*25*	*27*	*26*	*28*	*14*	*13*	*15*	*14*	*15*	*4*	*4*	*4*	*4*	*4*

Concentrations of anti-PT, anti-FHA, anti-PRN and anti-FIM serum IgG in culture positive volunteers over time. Results are given in IU/ml.

An increase is considered significant (bold numbers) if antibody level is doubled compared to pre-vaccination level and at least four times the minimum level of detection (4 IU/ml for PT and FHA, 8 IU/ml for PRN, and 4 EU/ml for FIM).

**Figure 5:**
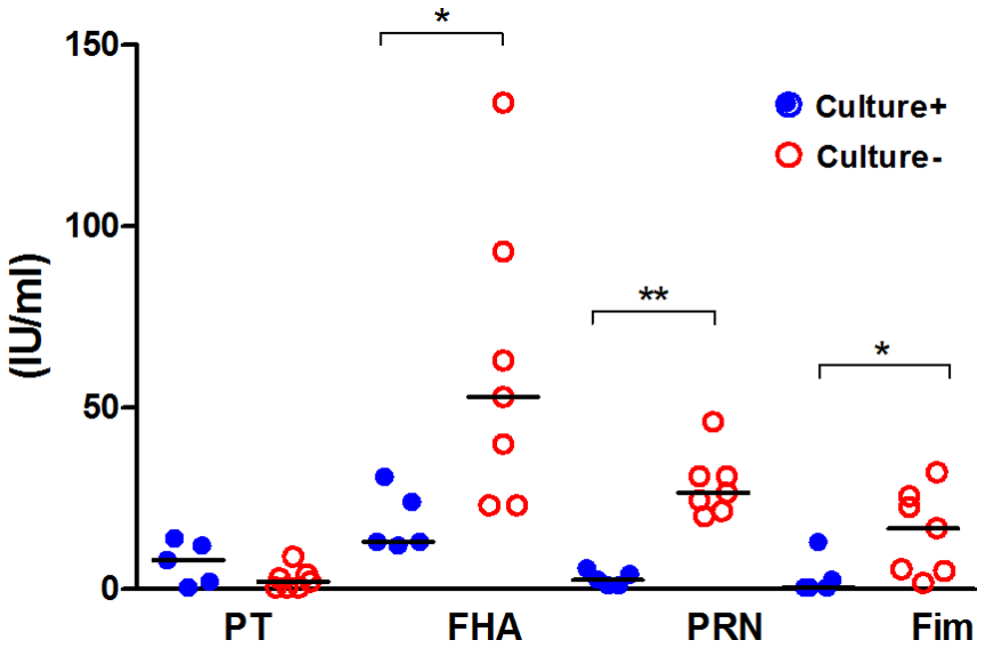
Pre-vaccination serum IgG levels to *B. pertussis* antigens in the seven culture-negative and the five culture-positive volunteers vaccinated with high dose BPZE1. PT, pertussis toxin; FHA, filamentous hemagglutinin; PRN, pertactin; and Fim, fimbriae 2/3. Culture positive volunteers are shown in blue filled circles and culture negative volunteers in red open circles. P-values were: PT p = 0.246; FHA p = 0.034 (*): PRN p = 0.006 (**); Fim p = 0.034 (*).

All volunteers had detectable pre-vaccination antibodies against at least one antigen (data not shown). Figure 5 shows the pre-vaccination serum IgG concentrations to the four antigens in the seven culture-negative and the five culture-positive volunteers vaccinated with the high dose. Although the numbers are small the pre-vaccination anti-FHA, anti-pertactin and anti-fimbriae antibody levels were significantly higher in the culture-negative subjects than in the culture-positive volunteers.

## Discussion

Live attenuated mucosal vaccines have a number of advantages over injectable subunit or inactivated vaccines, including the relatively low production cost and the ease of needle-free administration, which increases compliance. With a few exceptions [19], [20], most attenuated live vaccines are viral vaccines. BPZE1 is the first live attenuated bacterial vaccine specifically designed for the respiratory tract tested in humans. Since this was the first-in-man study of BPZE1, the number of volunteers was limited, and the primary objective of the study was the local and general safety assessment. As no data on a human dose range were available to guide us, three different doses, differing 100-fold from 10^3^ cfu on, were used sequentially. The doses chosen were based on the results of pre-clinical studies in mice [21]. A control group was included and evenly spread out over the study period. Without a control group it would not have been possible to evaluate whether the general or upper respiratory tract symptoms appeared in a normal or increased frequency among the vaccine recipients. The control group also reduces the risk of bias if the volunteers knew each other and shared experience of symptoms, as well as the bias of the investigators when evaluating the symptoms.

Except for one, which was not related to the vaccine, no SAE was noted. All other symptoms, mostly from the upper respiratory tract, headache and short-lasting fever, were trivial and evenly distributed in the placebo and three dosage groups. Seven of the 36 vaccinated individuals were colonized, and colonization was dose-dependent, with five of the seven in the high-dose group. Similar to what is seen with natural infection by *B. pertussis*, colonization was detected between days 4 and 28 after administration [22]. There was no significant difference in the occurrence of AE between the colonized and the non-colonized individuals. Thus, the vaccine can be considered safe for healthy male adults, which is a prerequisite for further studies in larger trials as well as in females and younger age groups.

A strong correlation was found between colonization and the induction of serum antibody responses to PT, FHA, pertactin and fimbriae. All colonized individuals mounted an antibody response to these antigens, most often to all four of them in contrast to the non-colonized volunteers. Interestingly, the antibody titers in the colonized low-dose vaccinee were not inferior to those of the high-dose subjects, suggesting that as long as colonization is established, regardless of the dose, antibodies to all B. pertussis antigens are induced. The genetic alteration of PT did not appear to affect its immunogenicity, since the median PT antibody levels were of the same magnitude as those that we have observed after a fifth dose of a combined tetanus, low dose diphtheria and acellular pertussis vaccine (TRIAXIS®, Sanofi Pasteur). That study included 115 adolescents aged 14–15 years primed with a five-component acellular pertussis vaccine at 3 and 5 months of age and boostered at 12 months and 5.5 years of age. In that study the level of PT antibodies was 20 IU/ml one month after the 5^th^ dose (manuscript in preparation). The PT antibodies induced in the culture positive individuals in this study were of the same magnitude, 27 IU/ml, one month after vaccination. Interestingly, five out of seven culture positive subjects responded against fimbriae 2/3, and one had an increased level but was defined as a non-responder, because he had a high pre-vaccination antibody level to fimbriae. This is in contrast to what has been shown after infection with *B. pertussis* when only 14% developed low levels of antibodies against fimbriae, unless vaccinated with vaccine containing fimbriae [23], [24].

Although increasing doses led to increased colonization and immune response frequencies, even at the highest vaccine dose not all volunteers were colonized. Several non-mutually exclusive explanations can account for this and will be the subject of future studies. It is possible that a dose of 10^7^ cfu is still not high enough, and that a higher dose is needed. A dose of 10^9^ cfu of virulent *B. pertussis* was necessary to establish an infection in non-human primates [25], and in a veterinary vaccine 10^8^ cfu/dose of live attenuated *Bordetella bronchiseptica* are used for the vaccination of dogs against kennel cough [26]. Also, the volume applied to each nostril (100 µl) may be suboptimal, and much larger volumes, up to 500 µl/nostril have been used for the application of live attenuated viral vaccines [27]. Alternatively, the vaccine may be administered by aerosol, which has been shown to be superior to nasal drops in an influenza vaccine trial [28]. Finally, it is also possible that pre-existing immunity may have prevented vaccine take. In that regard it is interesting to note that in the high-dose group the non-responders had pre-vaccination levels of antibodies to FHA, pertactin and fimbriae that were significantly higher than those of the responders. This may be due to unnoticed prior subclinical infection with *B. pertussis* or *Bordetella parapertussis* or to cross-reactivity with antigens from other bacteria. No relation between anti-PT IgG and colonization was found, but it should be noted that volunteers with pre-existing anti-PT IgG ≥20 IU/ml were excluded from the study. Whether pre-existing immunity prevents BPZE1 uptake and whether a higher dose or vaccine volume may overcome this will be addressed in future studies. These observations may also be relevant for the understanding of protective immunity against *B. pertussis* infection and support results from previous clinical studies Low anti-pertactin IgG levels have been related to susceptibility to *B. pertussis* infection while high anti-pertactin and high anti-PT antibody levels indicated protection following household exposure [29], [30].

In conclusion, this first-in-man study shows that live BPZE1 given intranasally is safe and able to colonize the nasopharynx of subjects with low pre-vaccination antibodies to FHA, pertactin and fimbriae. It induces serum IgG antibody responses in all colonized but not in non-colonized subjects. However, before BPZE1 can be used in the target population, the newborns, it will be necessary to first conduct further trials in older populations, such as adolescents and children of both sexes. The steps required to reach the target population strongly depend on the decisions made by the regulatory agencies of countries in which these trials can be performed. Discussions with several of these are currently under way. As a first live attenuated bacterial vaccine specifically designed for the respiratory tract, this proof-of-concept study may have a broad impact. Furthermore, recombinant BPZE1 derivatives producing heterologous antigens can be used for the development of multivalent vaccines [31]. Thus, the field of application of BPZE1 and its derivatives may potentially be very broad and extend far beyond that of a control tool against pertussis.

## Acknowledgments

The sponsor Inserm and the Strategic Planning and Clinical Trial Monitoring Committee (COSSEC) of Inserm represented by Sebastien Boy and Anne Puech and the European Clinical Research Infrastructure Network (ECRIN) for their support; Dominique Raze, Linda Kostic, Eva Hansson Pihlainen, Carina Bengtsson, Siri Wahlquist for skilful technical assistance at the laboratory; Ria Bral and Geert Deschamps from Innogenetics, Martine Vandermarliere and Marijke Verhaeghe from Innogenetics and Q-Biologicals for their input and work during the development and cGMP production; The study team at the Karolinska Trial Alliance (KTA) for vaccination, sample collection, and excellent surveillance of the volunteers at the clinic. The members of the Scientific Advisory Board and the Independent Data Monitoring Committee, Patrick Olin, Ragnar Norrby, Roland Dobbelaer, Odile Launay, Marta Granström and Micha Roumianzeff for invaluable advice during the study. Jérome Weinbach, Delphine Sondaz and Marie Clotteau from Inserm Transfert for management of the Child Innovac project.

## Supporting Information

Table S1 Frequency and Timing of Safety and Immunogenicity Measurements.
(DOCX)

Table S2 No Differences were found between Vaccine Groups Regarding Local and Systemic Solicited Adverse Events.
(DOCX)

Table S3 Differences versus High-Dose Group in Antigen Specific Serum IgG Levels.
(DOCX)

Table S4 Number of Local Solicited Adverse Events during Week 1 and 2 in the Placebo Group, the Culture Negative Subjects and the Culture Positive Subjects.
(DOCX)

Study Protocol S1 (PDF)

Consort Checklist S1 (DOC)

Method S1 Inclusion and Exclusion Criteria.
(DOCX)

Method S2 Nasopharyngeal Aspirate Cultivation.
(DOCX)

Method S3 Serum IgG Antibody Analyses.
(DOCX)
