# Supplementary material for: A Phase I Clinical Study of a Live Attenuated Bordetella pertussis Vaccine - BPZE1; A Single Centre, Double-Blind, Placebo-Controlled, Dose-Escalating Study of BPZE1 Given Intranasally to Healthy Adult Male Volunteers
Source: PLoS One. 2014 Jan 8;9(1):e83449. doi: 10.1371/journal.pone.0083449 (PMC3885431; doi:10.1371/journal.pone.0083449)
Supplement: Table S1 — Frequency and Timing of Safety and Immunogenicity Measurements. (DOCX) [file pone.0083449.s001.docx]

**TABLE S1. FREQUENCY AND TIMING OF SAFETY AND IMMUNOGENICITY MEASUREMENTS**

| **Study visit** | **1** | **2** | **3** | **4** | **5** | **6** | **7** | **7'** | **8** |
| --- | --- | --- | --- | --- | --- | --- | --- | --- | --- |
|  | **2-6 weeks prior to visit 2** | **Day 0** | **Day 4±1** | **Day 7±1** | **Day 11±1** | **Day 14±1** | **Day 28+7-1** | **Day 45±5** | **5-6 mon** |
| **Administrative requirements:** |  |  |  |  |  |  |  |  |  |
| -Recruitment, medical record number, screen form, | x |  |  |  |  |  |  |  |  |
| -Information and study consent^a^ | x |  |  |  |  |  |  |  |  |
| -Demographic information (age, gender, race) | x |  |  |  |  |  |  |  |  |
| **Randomisation and vaccination:** |  | x |  |  |  |  |  |  |  |
| **Clinical requirements:** |  |  |  |  |  |  |  |  |  |
| -Complete medical history | x |  |  |  |  |  |  |  |  |
| -Physical examination including vital signs^b^ | x | x | x | x | x | x | x | x | x |
| -Interim history/diary (AE documentation, concomitant medication)^c,d^ |  | x | x | x | x | x | x | x | x |
| **Safety labs:** |  |  |  |  |  |  |  |  |  |
| -ECG | x |  |  |  |  |  |  |  |  |
| -Hematology^e^ | x | x |  | x |  | x | x |  | x |
| -Blood chemistry^f^ | x |  |  |  |  |  |  |  |  |
| -Screening for HIV, hepatitis B and C^g^ | x |  |  |  |  |  |  |  |  |
| -Serum pertussis toxin IgG antibodies | x |  |  |  |  |  |  |  |  |
| -Urine analysis^h^ | x |  |  |  |  |  |  |  |  |
| -Total IgE | x |  |  |  |  |  | x |  | x |
| -Nasopharyngeal culture for B. pertussis |  |  | x | x | x | x | x^i^ | x^i^ |  |
| **Immunogenicity labs and sample archive:** | x | x |  | x |  | x | x |  | x |
| **TOTAL BLOOD VOLUME:** | 58.5 ml | 109.0 ml |  | 48.5 ml |  | 101.0 ml | 112.5 ml |  | 120.5 ml |

^(a)^ The ICF must be signed by the subject and investigator before vaccination with a study vaccine.

**^(b)^** At Visit 1, full physical examination (e.g. inspection of tonsils, palpation of glands, auscultation of pulm, measuring height and weight) including ECG and vital signs (oral temperature, blood pressure, heart rate, respiratory rate). For the following visits, physical exam will be restricted to general appearance, heart, lung, skin, nose, throat and vital signs.

**^(c)^** Adverse events (AE) occurring within at least 6 hours post-vaccination have to be recorded at the study centre and AE occurring from Day 0 to Day 28 have to be recorded in the Diary. If a vaccine-related AE is not resolved by Day 28, it must be followed up until resolution or stabilisation.

**^(d)^**  Serious adverse events (SAE) occurring from Visit 1 (screening) to Visit 8 have to be recorded at the study site In the Diary SAE will be recorded up to visit 7. If an SAE is not resolved at the last visit of the concerned subject, it must be followed up until resolution or stabilisation.

**^(e)^**  Hematology (haemoglobin, total and differential WBC, RBC, platelets)

**^(f)^**  Blood chemistry (potassium, calcium, sodium, creatinin, albumin, serum bilirubin, alkaline phosphatases, alaninaminotransferase (ALAT), aspartataminotransferase (ASAT), glutamyltransferase (GT), high sensitive C-reactive protein, blood glucose, thyreoidea stimulating hormone (TSH).

**^(g)^** Including HIV1/HIV2, HBsAg, anti-HBc and anti-HCV.

**^(h)^** Dipstick analysis of pH, erythrocytes, leucocytes, protein, glucose, ketones and bacteria (nitrite) and the drugs cocaine, amphetamine, cannabis, morphine, benzodiazepines, and methylenedioxymethamphetamine.

**^(i)^** If positive culture after 4 weeks a new nasopharyngeal sample should be collected 2-3 weeks later for culture.
