# Supplementary material for: A Phase I Clinical Study of a Live Attenuated Bordetella pertussis Vaccine - BPZE1; A Single Centre, Double-Blind, Placebo-Controlled, Dose-Escalating Study of BPZE1 Given Intranasally to Healthy Adult Male Volunteers
Source: PLoS One. 2014 Jan 8;9(1):e83449. doi: 10.1371/journal.pone.0083449 (PMC3885431; doi:10.1371/journal.pone.0083449)
Supplement: Table S2 — No Differences were found between Vaccine Groups Regarding Local and Systemic Solicited Adverse Events. (DOCX) [file pone.0083449.s002.docx]

### TABLE S2. NO DIFFERENCES WERE FOUND BETWEEN VACCINE GROUPS REGARDING LOCAL AND SYSTEMIC SOLICITED ADVERSE EVENTS

|  | **Adverse event** | **P-value (week 1)** | **P-value (week 2)** | **P-value (week 3)** | **P-value (week 4)** |
| --- | --- | --- | --- | --- | --- |
| **Local solicited adverse events** | Cough | 0.551 | 0.383 | >0.999 | >0.999 |
|  | Nasal congestion | 0.483 | 0.533 | >0.999 | >0.999 |
|  | Epistaxis | 0.600 | >0.999 | >0.999 | >0.999 |
|  | Rhinorrhoea | 0.566 | 0.373 | >0.999 | 0.693 |
|  | Sneezing | 0.134 | 0.584 | >0.999 | >0.999 |
|  | Ear pain | 0.234 | >0.999 | >0.999 | >0.999 |
|  | Eye pain | >0.999 | >0.999 | >0.999 | >0.999 |
|  | Dyspnoea | >0.999 | >0.999 | >0.999 | >0.999 |
| **Systemic solicited adverse events** | Tiredness | 0.186 | 0.307 | >0.999 | >0.999 |
|  | Headache | 0.918 | >0.999 | >0.999 | >0.999 |
|  | Pyrexia | >0.999 | 0.600 | >0.999 | >0.999 |

Fishers exact test was used for statistical comparisons.
