# Supplementary material for: A Phase I Clinical Study of a Live Attenuated Bordetella pertussis Vaccine - BPZE1; A Single Centre, Double-Blind, Placebo-Controlled, Dose-Escalating Study of BPZE1 Given Intranasally to Healthy Adult Male Volunteers
Source: PLoS One. 2014 Jan 8;9(1):e83449. doi: 10.1371/journal.pone.0083449 (PMC3885431; doi:10.1371/journal.pone.0083449)
Supplement: Table S3 — Differences versus High-Dose Group in Antigen Specific Serum IgG Levels. (DOCX) [file pone.0083449.s003.docx]

### TABLE S3. DIFFERENCES VERSUS HIGH-DOSE GROUP IN ANTIGEN SPECIFIC SERUM IgG LEVELS (p-values are shown)

|  | **28 DAYS AFTER VACCINATION** | | | **5-6 MONTHS AFTER VACCINATION** | | |
| --- | --- | --- | --- | --- | --- | --- |
|  | **Low dose** | **Medium dose** | **Placebo** | **Low dose** | **Medium dose** | **Placebo** |
| **FHA** | 0.062 | *0.025** | 0.062 | *0.046** | *0.022** | *0.046** |
| **PRN** | 0.100 | *0.016** | *0.033** | 0.144 | *0.009** | *0.023** |
| **Fim** | 0.077 | *0.040** | 0.071 | 0.117 | *0.036** | 0.059 |

Kruskal-Wallis one-way ANOVA was used followed by pairwise comparisons if the overall test showed a significant test result. Significant differences are in *italics* and indicated with *.
