# Supplementary material for: A Phase I Clinical Study of a Live Attenuated Bordetella pertussis Vaccine - BPZE1; A Single Centre, Double-Blind, Placebo-Controlled, Dose-Escalating Study of BPZE1 Given Intranasally to Healthy Adult Male Volunteers
Source: PLoS One. 2014 Jan 8;9(1):e83449. doi: 10.1371/journal.pone.0083449 (PMC3885431; doi:10.1371/journal.pone.0083449)
Supplement: Table S4 — Number of Local Solicited Adverse Events during Week 1 and 2 in the Placebo Group, the Culture Negative Subjects and the Culture Positive Subjects. (DOCX) [file pone.0083449.s004.docx]

**TABLE S4. NUMBER OF LOCAL SOLICITED ADVERSE EVENTS DURING WEEK 1 AND 2 IN THE PLACEBO GROUP, THE CULTURE NEGATIVE SUBJECTS AND THE CULTURE POSITIVE SUBJECTS**

|  |  | **WEEK 1** |  |  |  | **WEEK 2** |  |  |
| --- | --- | --- | --- | --- | --- | --- | --- | --- |
| **Adverse event** | **Intensity** | **Placebo**  N=12 | **Culture negative**  N=28 | **Culture positive**  N=7 |  | **Placebo**  N=12 | **Culture negative**  N=28 | **Culture positive**  N=7 |
| **Cough** | None | 10 | 21 | 7 |  | 11 | 24 | 6 |
|  | Mild | 1 | 6 | 0 |  | 0 | 3 | 1 |
|  | Moderate | 0 | 1 | 0 |  | 1 | 1 | 0 |
|  | High | 1 | 0 | 0 |  | 0 | 0 | 0 |
|  | All intensities | 2 | 7 | 0 |  | 1 | 4 | 1 |
| **Nasal congestion** | None | 6 | 20 | 4 |  | 11 | 22 | 4 |
|  | Mild | 4 | 7 | 3 |  | 1 | 5 | 3 |
|  | Moderate | 2 | 1 | 0 |  | 0 | 1 | 0 |
|  | High | 0 | 0 | 0 |  | 0 | 0 | 0 |
|  | All intensities | 6 | 8 | 3 |  | 1 | 6 | 3 |
| **Epistaxis** | None | 12 | 25 | 7 |  | 12 | 28 | 7 |
|  | Mild | 0 | 3 | 0 |  | 0 | 0 | 0 |
|  | Moderate | 0 | 0 | 0 |  | 0 | 0 | 0 |
|  | High | 0 | 0 | 0 |  | 0 | 0 | 0 |
|  | All intensities | 0 | 3 | 0 |  | 0 | 0 | 0 |
| **Rhinorrhoea** | None | 5 | 18 | 4 |  | 10 | 23 | 4 |
|  | Mild | 5 | 9 | 2 |  | 2 | 4 | 3 |
|  | Moderate | 2 | 1 | 1 |  | 0 | 1 | 0 |
|  | High | 0 | 0 | 0 |  | 0 | 0 | 0 |
|  | All intensities | 7 | 10 | 3 |  | 2 | 5 | 3 |
| **Sneezing** | None | 6 | 20 | 5 |  | 9 | 25 | 4 |
|  | Mild | 6 | 7 | 2 |  | 3 | 2 | 3 |
|  | Moderate | 0 | 1 | 0 |  | 0 | 1 | 0 |
|  | High | 0 | 0 | 0 |  | 0 | 0 | 0 |
|  | All intensities | 6 | 8 | 2 |  | 3 | 3 | 3 |
| **Ear problem** | None | 12 | 27 | 6 |  | 12 | 26 | 7 |
|  | Mild | 0 | 1 | 1 |  | 0 | 2 | 0 |
|  | Moderate | 0 | 0 | 0 |  | 0 | 0 | 0 |
|  | High | 0 | 0 | 0 |  | 0 | 0 | 0 |
|  | All intensities | 0 | 1 | 1 |  | 0 | 2 | 0 |
| **Eye pain** | None | 12 | 27 | 7 |  | 12 | 28 | 7 |
|  | Mild | 0 | 1 | 0 |  | 0 | 0 | 0 |
|  | Moderate | 0 | 0 | 0 |  | 0 | 0 | 0 |
|  | High | 0 | 0 | 0 |  | 0 | 0 | 0 |
|  | All intensities | 0 | 1 | 0 |  | 0 | 0 | 0 |
| **Dyspnoea** | None | 12 | 26 | 7 |  | 12 | 28 | 7 |
|  | Mild | 0 | 2 | 0 |  | 0 | 0 | 0 |
|  | Moderate | 0 | 0 | 0 |  | 0 | 0 | 0 |
|  | High | 0 | 0 | 0 |  | 0 | 0 | 0 |
|  | All intensities | 0 | 2 | 0 |  | 0 | 0 | 0 |
