# Supplementary material for: A Phase I Clinical Study of a Live Attenuated Bordetella pertussis Vaccine - BPZE1; A Single Centre, Double-Blind, Placebo-Controlled, Dose-Escalating Study of BPZE1 Given Intranasally to Healthy Adult Male Volunteers
Source: PLoS One. 2014 Jan 8;9(1):e83449. doi: 10.1371/journal.pone.0083449 (PMC3885431; doi:10.1371/journal.pone.0083449)
Supplement: Study Protocol S1 — (PDF) [file pone.0083449.s005.pdf]

CONFIDENTIAL

A phase 1, single centre, dose-escalating, placebo-controlled study of a genetically modified B. pertussis strain given as a single intranasal dose to healthy adult male volunteers

CLINICAL STUDY PROTOCOL

EudraCT Number:	2010-019936-11
Study Identification Number:	BT0604
Short name:	Child Innovac I
Phase:	I
Principal Clinical Investigator:	Nabil Al-Tawil, MD, PhD<br>Karolinska Trial Alliance<br>Karolinska University Hospital<br>SE- 141 86 Stockholm<br>Phone: +46 8 51 77 50 62<br>Fax: +46 8 58 58 69 20<br>Email: nabil.al-tawil@karolinska.se
Medical Advisor and Safety Officer on behalf of SMI	Birger Trollfors, MD, Professor<br>Dept. of Immunology and Vaccinology<br>Swedish Institute for Infectious Disease Control<br>171 82 Solna<br>Sweden<br>Phone: +46 31 343 52 17<br>Email : birger.trollfors@vgregion.se
Principal Laboratory Investigator/ Swedish 'corresponding sponsor' with Swedish authorities	Rigmor Thorstensson, PhD, Ass. Prof, Head of Department<br>Dept. of Immunology and Vaccinology<br>Swedish Institute for Infectious Disease Control<br>171 82 Solna<br>Sweden<br>Phone: +46 70 256 26 70<br>Fax : +46 8 33 74 60<br>Email : rigmor.thorstensson@smi.se
Sponsor:	INSERM<br>101, rue de Tolbiac, F-75654 Paris, Cedex 13, France<br>Phone:+ 33 1 44 23 60 44<br>Fax: + 33 1 44 23 67 11
Scientific responsible INSERM:	Camille Locht, PhD, professor<br>Inserm – Institut Pasteur de Lille<br>1, rue du Prof. Calmette<br>F-59019 Lille Cedex, France<br>Phone: +33 3 20 87 11 51;<br>Fax: + 33 3 20 87 11 58;<br>e-mail: camille.locht@pasteur-lille.fr;
Protocol Version:	**2.0**
Protocol Date:	**100930**

CONFIDENTIAL

Institut national
de la santé et de la recherche médicale

Information contained in this document is the property of INSERM and is confidential. This information will not be disclosed to third parties without written authorization from INSERM except to obtain informed consent from those persons to whom the vaccine will be administered, and only to comply with legal regulations. No part of this document may be reproduced, stored in a retrieval system or transmitted in any form or by any means - electronic, mechanical, recording or otherwise - without the prior permission of INSERM.

Table of contents

I. GENERAL INFORMATION 7

I.1. CONTACT LIST 7

Sponsor, Investigators and Study Centre........ 7

I.2. GLOSSARY OF ABBREVIATIONS 9

I.3. SYNOPSIS........ 10

I.4. PROCEDURAL FLOW-CHART 16

II. IMPORTANT MEDICAL PROCEDURES TO BE FOLLOWED BY THE INVESTIGATOR 17

II.1. EYE EXPOSURE........ 17

II.2. STOPPING CRITERIA FOR DOSE ESCALATION/CONTINUATION OF STUDY 17

II.3. METHOD OF BLINDING AND BREAKING THE STUDY BLIND 17

III. INTRODUCTION 18

III.1. BACKGROUND AND STUDY RATIONALE........ 18

III.2. BENEFIT/RISK AND ETHICAL ASSESSMENT........ 18

IV. STUDY OBJECTIVES........ 21

IV.1. SAFETY (PRIMARY)........ 21

IV.2. ATTACHMENT OF THE BPZE1 STRAIN TO THE NASOPHARYNGEAL MUCOSA (SECONDARY) 21

IV.3. IMMUNOGENICITY (SECONDARY) 21

V. SELECTION OF VOLUNTEERS........ 22

V.1. INCLUSION CRITERIA........ 22

V.2. EXCLUSION CRITERIA 22

V.3. TEMPORARY CONTRAINDICATIONS 23

VI. STUDY PLAN AND DESIGN 24

VI.1. PLANNED STUDY CALENDAR 24

VI.2. GENERAL STUDY PLAN AND DESIGN........ 24

VI.3. STUDY VISITS........ 25

VI.3.1. First contact 25

VI.3.2. Visit 1, screening 2-6 weeks before vaccination 26

VI.3.3. Visit 2, day 0, vaccination 26

VI.3.4. Visit 3, 3-5 days after visit 2........ 27

VI.3.5. Visit 4, 7±1 days after visit 2........ 27

VI.3.6. Visit 5, 11±1 days after visit 2........ 28

VI.3.7. Visit 6, 14±1 days after visit 2........ 28

VI.3.8. Visit 7, 28±1 days after visit 2 .... *28*
VI.3.9. Visit 7', 45±5 days after visit 2 .... *29*
VI.3.10. Visit 8, 5-6 months after visit 2 .... *29*
VI.4. SCREENING NUMBER AND RANDOMISATION PROCEDURE 29
Subject *29*
Randomisation procedure and blinding *29*

VII. PRIOR AND CONCOMITANT MEDICATIONS AND NON-STUDY VACCINES 31

VII.1. PRIOR MEDICATIONS AND VACCINATION 31
VII.2. CONCOMITANT MEDICATIONS AND NON-STUDY VACCINES 31
VII.3. NOT ALLOWED MEDICATIONS & VACCINES 31

VIII. WITHDRAWALS/STUDY TERMINATION 32

IX. FOLLOW UP OF VOLUNTEERS 33

X. INVESTIGATIONAL MEDICINAL PRODUCTS I.E. STUDY VACCINE 34

X.1. IDENTITY OF STUDY VACCINE 34
X.2. SUPPLY OF STUDY VACCINE 34
X.3. DOSES AND TREATMENT REGIMENS 34
X.4. LABELLING AND PACKAGING OF STUDY VACCINE 34
X.5. SHIPMENT OF STUDY VACCINE 36
X.6. STORAGE OF STUDY VACCINE 36
X.7. HANDLING AND ADMINISTRATION OF STUDY VACCINE TO PARTICIPANTS 36
X.8. ACCOUNTABILITY OF STUDY VACCINE 37
X.9. DESTRUCTION OF STUDY VACCINE 37

XI. COLLECTION OF STUDY VARIABLES 38

XI.1. RECORDING OF DATA 38
XI.2. SCREENING AND DEMOGRAPHIC PROCEDURES 38
XI.3. SAFETY 38
XI.4. LABORATORY SAFETY ASSESSMENT 38

XII. COLLECTION, TRANSPORT AND STORAGE OF BIOLOGICAL SAMPLES 39

XII.1. RESPONSIBLE LABORATORIES 39
XII.2. LABELLING OF SAMPLES 39
XII.3. BLOOD SAMPLES 39
Blood Collection *39*
Blood samples transport *40*
Serum samples management and storage *40*
Peripheral blood monocytoid cells (PBMC) management and storage *40*

XII.4. SALIVA SAMPLES 41
Saliva collection *41*
Saliva samples transport, management and storage *41*
XII.5. NASOPHARYNGEAL SAMPLES 41
Nasopharyngeal aspirate collection *41*
Nasopharyngeal samples transport, management and storage *42*
XII.6. URINE SAMPLES 42
Urine collection *42*
Urine samples and transport *42*
XII.7. WITHDRAWAL OF INFORMED CONSENT FOR DONATED BIOLOGICAL SAMPLES 42
XII.8. BIOBANK STORAGE OF BIOLOGICAL SAMPLES 42

XIII. RECORDING AND REPORTING OF ADVERSE EVENTS 43

XIII.1. DEFINITIONS 43
Adverse Event (AE) *43*
Serious Adverse Event (SAE) *43*
Adverse Reaction (AR) *44*
Relationship of an Adverse Event to the Study Vaccine *44*
Intensity of an AE / AR *44*
XIII.2. RECORDING OF SAFETY PARAMETERS 45
Immediate AE *45*
Local AE from the respiratory tract *45*
Systemic AE *45*
Temperature *45*
Serious Adverse Event (SAE) *46*
General requirements *46*
XIII.3. REPORTING OF ADVERSE EVENTS BY THE INVESTIGATOR 46
Reporting of SAE *47*
Reporting of other safety information *47*
XIII.4. ASSESSMENT AND REPORTING OF ADVERSE EVENTS 47
XIII.5. REPORTING OF SUSAR 48
XIII.6. ANNUAL SAFETY REPORT 48
XIII.7. INDEPENDENT DATA MONITORING COMMITTEE (IDMC) 48

XIV. EVALUATION CRITERIA 50

XIV.1. SAFETY (PRIMARY) 50
XIV.2. ATTACHMENT OF THE BPZE1 STRAIN TO THE NASOPHARYNGEAL MUCOSA (SECONDARY) 50
XIV.3. IMMUNOGENICITY (SECONDARY) 50
Method for measurement *51*

XV. STATISTICAL CONSIDERATIONS 52
XV.1. LOCAL SYMPTOMS 52
XV.2. HEMATOLOGY 52
XV.3. IMMUNOGENICITY ANALYSIS 52
XVI. ADMINISTRATIVE AND REGULATORY REQUIREMENTS 53
XVI.1. STUDY DOCUMENTS AND RETENTION OF RECORDS 53
XVI.2. RECORDING OF DATA 53
XVI.3. DATA QUALITY ASSESSMENT 53
XVI.4. DATA MANAGEMENT 54
XVI.5. ETHICS COMMITTEE 54
XVI.6. HEALTH AUTHORITIES 55
XVI.7. CONFIDENTIALITY 55
XVI.8. COMPLIANCE WITH LAW AND AUDIT 55
XVI.9. INSURANCE 56
XVI.10. PUBLICATIONS AND DATA PROPERTY 56
XVII. STUDY MANAGEMENT 57
XVII.1. TRAINING OF STUDY SITE PERSONNEL 57
XVII.2. MONITORING OF THE STUDY 57
XVIII. LIST OF APPENDICES 58
APPENDIX 1 59
XVIII.1. ENVIRONMENT RISK ASSESSMENT FOR A VACCINE TO BE USED IN THE CHILD INNOVAC PHASE I CLINICAL TRIAL / MILJÖRISKANALYS FÖR VACCIN INGÅENDE I CHILD INNOVAC 1 59
APPENDIX 2 69
XVIII.2. OFFICIAL FRENCH AGREEMENT ON THE DOWNGRADING OF BPZE1 AS A CLASS I ORGANISM 69
APPENDIX 3 70
XVIII.3. DECLARATION OF HELSINKI 70
APPENDIX 4 74
XVIII.4. GUIDANCE FOR INDUSTRY. TOXCICITY GRADING SCALE FOR HEALTHY ADULT AND ADOLESCENT VOLUNTEERS IN PREVENTIVE VACCINE CLINICAL TRIALS 74

I. General information

I.1. CONTACT LIST

Sponsor, Investigators and Study Centre

Sponsor:
Anne Puech, PhD
INSERM
101, rue de Tolbiac, F-75654 Paris, Cedex 13, France
Phone:+ 331 44 23 67 78
Fax: + 33 1 44 23 67 11
Email: anne.puech@inserm.fr

Principal Clinical Investigator
Nabil Al-Tawil, MD, PhD, Consultant
Karolinska Trial Alliance
Karolinska University Hospital
SE- 141 86 Stockholm
Phone: +46 8 51 77 50 62
Fax: +46 8 5858 69 20
Email: nabil.al-tawil@karolinska.se

Medical Advisor and Safety Officer on behalf of SMI
Birger Trollfors, MD, Professor
Dept. of Immunology and Vaccinology
Swedish Institute for Infectious Disease Control
171 82 Solna
Sweden
Phone: +46 31 343 52 17
Email : birger.trollfors@vgregion.se

Principal Laboratory Investigator/Swedish 'corresponding sponsor' with the Swedish authorities
Rigmor Thorstensson, PhD, Assoc. Prof, Head of Department
Dept. of Immunology and Vaccinology
Swedish Institute for Infectious Disease Control
171 82 Solna
Sweden
Phone: +46 70 256 26 70
Fax: +46 8 33 74 60
Email: rigmor.thorstensson@smi.se

Legal partner with the Swedish authorities
Johan Carlson, M.D. PhD, Director General
Swedish Institute for Infectious Disease Control
171 82 Solna
Sweden
Phone+46 8 457 23 10
Email: Johan.carlson@smi.se

Study Centre

Karolinska Trial Alliance
Karolinska University Hospital
141 86 Stockholm
Contact person: Eva-Lena Forsberg
Phone: +46 70 484 13 74
Email: eva-lena.forsberg@karolinska.se

Scientific Responsible INSERM

Camille Locht, PhD, Professor
INSERM – Institut Pasteur de Lille
1, rue du Prof. Calmette
F-59019 Lille Cedex, France
Phone: +33 3 20 87 11 51;
Fax: + 33 3 20 87 11 58;
E-mail: camille.locht@pasteur-lille.fr;

Responsible for monitoring

Karolinska Trial Alliance
Karolinska University Hospital, M62
141 86 Stockholm
Contact person: Peter Westerling
Phone: +46 8-517 750 35
Mobile: 073-699 44 81
E-mail: peter.westerling@karolinska.se

Responsible for pharmacovigilance

Béatrice Barraud
INSERM
101, rue de Tolbiac, F-75654 Paris, Cedex 13, France
Phone:+ 33 1 44 23 67 29
Fax: + 33 1 44 23 67 10
Email: rqrc.siege@inserm.fr

I.2. GLOSSARY OF ABBREVIATIONS

**AE**	Adverse Event
**AR**	Adverse Reaction
**ANRS**	Agence Nationale de Recherche sur le Sida
**Bpm**	Beats per minute
**CIOMS**	Council of International Organisation of Medicinal Sciences
**CRA**	Clinical Research Associate
**CRF**	Case Report Form
**CRO**	Contract Research Organisation
**CSR**	Clinical Study Report
**ECG**	Electrocardiography
**ELISA**	Enzyme Immuno Assay / Enzyme-Linked Immunosorbent Assay
**ELISpot**	Enzyme Linked Immuno Spot
**EMEA**	European Medicines Agency
**EU/mL**	EIA Unit per milliliter
**FHA**	Filamentous Haemagglutinin
**FIM**	Fimbriae types 2 + 3
**FVFS**	First Visit First Subject
**FVLS**	First Visit Last Subject
**GMC**	Geometric Mean Concentration
**IB**	Investigator's Brochure
**ICF**	Informed Consent Form
**IDMC**	Independent Data Monitoring Committee
**IMP**	Investigational Medicinal Product
**INSERM**	"French Health and Medical Research National Institute"
**ISF**	Investigator Site File
**IU**	International Unit
**IU/mL**	International Unit per millilitre
**IWRS**	Interactive Web Response System
**LVFS**	Last Visit First Subject
**LVLS**	Last Visit Last Subject
**MedDRA**	Medical Dictionary for Regulatory Activities
**mL**	Millilitre
**PBMC**	Peripheral blood mononuclear cells
**PRN**	Pertactin
**PT**	Pertussis toxin
**RBC**	Red blood cells
**RT**	Room temperature
**SAE**	Serious Adverse Event
**SAR**	Serious Adverse Reaction
**SMI**	Smittskyddsinstitutet (Swedish Institute for Infectious Disease Control)
**SOP**	Standardised Operational Procedure
**SUSAR**	Suspected Unexpected Serious Adverse Reaction
**ULB**	Université Libre de Bruxelles
**USN**	Unique subject number
**WBC**	White blood cells

I.3. SYNOPSIS

**Study title**	A phase 1, single centre, dose-escalating, placebo-controlled study of a genetically modified B. pertussis strain given as a single intranasal dose to healthy adult male volunteers
**Study Identification Number:**	**BT0604**
**Sponsor**	INSERM

**Investigational product**	Live attenuated *Bordetella pertussis* BPZE1 strain or placebo
**Active ingredient**	Live attenuated *Bordetella pertussis* BPZE1 bacteria
**Phase**	I
Medical Advisor and Safety Officer on behalf of SMI	Birger Trollfors, MD, professor<br>Dept. of Immunology and Vaccinology<br>Swedish Institute for Infectious Disease Control<br>171 82 Solna<br>Sweden<br>Phone: +46 31 343 52 17<br>Email : birger.trollfors@vgregion.se
**Planned study period**	First visit of the first subject: August 2010<br>First visit of the last subject: December 2010<br>Last visit of the last subject: June 2011
**Objectives**	**Primary objective**<br>•To assess the general safety and local tolerability in the respiratory tract of a single ascending dose of the genetically modified B. pertussis strain<br>**Secondary objectives**<br>• To see if the modified B. pertussis strain has the ability to colonise the human respiratory tract<br>• To assess the B and T cell immune responses to 5 different B .pertussis antigens: pertussis toxin, filamentous hemagglutinin, pertactin, fimbriae and whole Bordetella pertussis cell lysates. Immune responses will be determined by serum IgG and IgA, IgA in saliva and nasopharyngeal aspirate, cytokines and numbers of effector and memory T and B cells after stimulation with the various B. pertussis antigens. Except for serum antibodies, the immunological assays can be regarded as exploratory.
**Sample size**	**Total to be included:** 48 (3 groups of different escalating doses – 12 active and 4 placebo per group)
**Study plan**	A dose-escalating study with a control group.<br>The volunteers will be recruited in a step-wise fashion with 16 individuals in each of three groups.<br><br>Group 1: 12 individuals will be vaccinated once intranasally with 1,000 colony forming units (cfu) of the organism. 4 individuals will get placebo (the diluent alone). Note! 2 active then 10 active and 4 placebo.<br><br>Group 2: 12 individuals will be vaccinated once intranasally with 100,000 colony forming units (cfu) of the organism. 4 individuals will get placebo (the diluent alone). Note! 2 active then 10 active and 4 placebo.<br><br>Group 3: 12 individuals will be vaccinated once intranasally with 10,000,000 colony forming units (cfu) of the organism. 4 individuals will get placebo (the diluent alone). Note! 2 active then 10 active and 4 placebo.

**Study title**	A phase 1, single centre, dose-escalating, placebo-controlled study of a genetically modified B. pertussis strain given as a single intranasal dose to healthy adult male volunteers
**Study Identification Number:**	**BT0604**
**Sponsor**	INSERM

	The administration of vaccine or diluent alone will be performed in a double-blind fashion with administration of coded vials with exception for the two first study subjects in each dosage group, who will receive active substance (unblinded).<br><br><u>Visit 1 (screening 2-6 weeks before vaccination)</u><br><br>The subjects will be informed and will provide written informed consent (ICF) to participate in the study.<br>Full physical examination, ECG, and vital signs.<br><br>Blood samples for analysis of cell blood counts (haemoglobin, total and differential WBC (RBC platelets), blood chemistry (potassium, calcium, sodium, creatinin, albumin, bilirubin, alkaline phosphatases, alaninaminotransferase (ALAT), aspartataminotransferase (ASAT), glutamyltransferase (GT), C-reactive protein, blood glucose, thyroid stimulating hormone (TSH), antibodies against pertussis toxin and total IgE. Blood samples for analysis for infection with HIV, hepatitis B and C. Urine samples for dipstick analysis of pH, erythrocytes, leucocytes, protein, glucose, ketones and bacteria (nitrite) and the drugs cocaine, amphetamine, cannabis, morphine, benzodiazepines, and methylenedioxymethamphetamine.<br><br><u>Visit 2 (day 0 the day of vaccination)</u><br><br>Limited physical examination and measure of vital signs.<br>Cell blood counts (haemoglobin, total and differential WBC, RBC, platelets).<br>Blood, saliva and nasopharyngeal samples for immunological assays.<br>Vaccination. The volunteer will stay at the study centre for 6 hours after administration of the vaccine.<br>Information to the volunteer how to fill in the Diary daily concerning adverse events.<br>Review inclusion/exclusion criteria.<br><br><u>Visit 3 (4±1 days after vaccination)</u>.<br><br>Limited physical examination and measure of vital signs.<br>Structured questionnaire concerning general and local adverse events and a question about unsolicited adverse events.<br>Nasopharyngeal culture for B. pertussis and nasopharyngeal samples for immunological assays.<br><br><u>Visit 4 (7±1 days after vaccination).</u><br><br>Limited physical examination and measure of vital signs.<br>Structured questionnaire concerning general and local adverse events and a question about unsolicited adverse events.<br>Cell blood counts (haemoglobin, total and differential WBC, RBC, platelets).<br>Nasopharyngeal culture for B. pertussis<br>Blood, nasopharyngeal and saliva samples for immunological assays.<br><br><u>Visit 5 (11±1 days after vaccination).</u><br><br>Limited physical examination and measure of vital signs.<br>Structured questionnaire concerning general and local adverse events and a question about unsolicited adverse events.<br>Nasopharyngeal culture for B. pertussis and nasopharyngeal samples for immunological assays.<br><br><u>Visit 6 (14±1 days after vaccination).</u>

**Study title**	A phase 1, single centre, dose-escalating, placebo-controlled study of a genetically modified B. pertussis strain given as a single intranasal dose to healthy adult male volunteers
**Study Identification Number:**	**BT0604**
**Sponsor**	INSERM

	Limited physical examination and measure of vital signs.<br>Questions concerning general and local adverse events and questions about unsolicited adverse events.<br>Cell blood counts (haemoglobin, total and differential WBC, RBC, platelets).<br>Nasopharyngeal culture for B. pertussis.<br>Blood, nasopharyngeal and saliva samples for immunological assays.<br><br>Visit 7 (28 (-1; + 7) days after vaccination).<br><br>Limited physical examination and measure of vital signs.<br>Questions concerning general and local adverse events and questions about unsolicited adverse events.<br>Subject diary collected and filed.<br><br>Cell blood counts (haemoglobin, total and differential WBC, RBC, platelets).<br>Nasopharyngeal culture for B. pertussis. If positive culture after 4 weeks a new nasopharyngeal sample should be collected 2-3 weeks later for culture.<br>Blood, nasopharyngeal and saliva samples for immunological assays.<br>Blood sample for total IgE.<br><br>Visit 7' (45±5 days after vaccination).<br><br>Nasopharyngeal culture for B. pertussis. This visit will only be scheduled if B. pertussis was detected in the sample collected on the visit day 28±1.<br><br>Visit 8 (5 - 6 months after vaccination).<br><br>Questions concerning unsolicited adverse events.<br>Cell blood counts (haemoglobin, total and differential WBC, rbc, platelets).<br>Blood, nasopharyngeal and saliva samples for immunological assays.<br>Blood sample for total IgE.
**Inclusion criteria**	Subject will be included in the study if he meets all the following criteria:<br>1. Healthy male born between 1979 and 1991 who has not experienced clinical pertussis (lab. verified) during the past 10 years and who has not been vaccinated with any pertussis vaccine.<br>2. Informed consent form signed by the subject.<br>3. Subject shall be able to attend all scheduled visits and to understand and comply with the study procedures (e.g. able to read and write Swedish).
**Exclusion criteria**	If any of the following criteria are met, the subject must not be included in the study:<br>1. Individuals with pertussis toxin serum IgG antibodies ≥20 International units/mL.<br>2. Blood pressure after resting ≥ 150/90 mmHg.<br>3. Heart rate after resting ≥80 bpm.<br>4. Respiratory rate after resting ≥ 20/minute.<br>5. Unwillingness to refrain from the use of nicotine products from screening through day 28.<br>6. Use of narcotic drugs and/or a history of drug/alcohol abuse whitin the past 2 years prior to screening<br>7. The subject has donated blood or suffered from blood loss of at least 450 ml (1 unit of blood) within 60 days prior to screening or donated plasma within 14 days prior to screening.

**Study title**	A phase 1, single centre, dose-escalating, placebo-controlled study of a genetically modified B. pertussis strain given as a single intranasal dose to healthy adult male volunteers
**Study Identification Number:**	**BT0604**
**Sponsor**	INSERM

	8. Receipt of immunoglobulin, blood derived products, systemic corticosteroids or other immunosuppressant drugs within 90 days prior to day 0.<br>9. Use of corticosteroids in the respiratory tract(e.g. nasal steroids, inhaled steroids) whitin 30 days prior to day 0.<br>10. Use of herbal medications or dietary supplements within 7 days prior to day 0 at the discretion of the investigator. Unwillingness to refrain from herbal medications or dietary supplements within 30 days after day 0 at the discretion of the investigator.<br>11. Receipt of a vaccine within the last 30 days prior to day 0 or planned vaccination within the next 30 days after day 0.<br>12. Evolving encephalopathy not attributable to another identifiable cause within 7 days of administration of a previous dose of any vaccine.<br>13. Known hypersensitivity to any component of the study vaccine.<br>14. Current participation in any other clinical trial or participation (and during the whole study) in any clinical trial in the previous 3 months prior to day 0.<br>15. Inability to adhere to the protocol, including plans to move from the area.<br>16. Family (first degree) history of congenital or hereditary immunodeficiency.<br>17. Infection with HIV, hepatitis B or C.<br>18. Any medical condition which, in the opinion of the investigator, might interfere with the evaluation of the study objectives.<br>19. Clinically significant abnormal laboratory values at the discretion of the investigator.<br>20. Person in frequent contact with children less than 1 year of age (father, childcare worker, nurse, etc…) or residence in the same household as persons with known immunodeficiency including persons on immunosuppressant therapy.
**Temporary contra-indications to vaccination**	Reschedule vaccination if<br>Febrile illness (oral temperature ≥ 38.0°C) at the day of vaccination.<br>Ongoing rhinitis and/or rhinoconjunctivitis.
**Investigational product**	Live attenuated *Bordetella pertussis* BPZE1 strain or placebo
**Presentation**	Vial
**Dose**	Dose 2 x 0.1 mL (0.1 mL per nostril)
**Route**	Nasal
**Duration of treatment:**	Single administration
**Excluded non-study vaccines/therapies during study**	The following therapies should be avoided during the first month after vaccination. If it is necessary to give a medication or a vaccine, this shall for obvious reasons be permitted. All medications and vaccines will be documented in the CRF. The volunteer will be followed as pre-planned for adverse events. The immunological part of the study can also continue, but the results from the volunteer must be reported separately under the following conditions:<br>• Antibacterial agents effective against B pertussis.

Study title	A phase 1, single centre, dose-escalating, placebo-controlled study of a genetically modified B. pertussis strain given as a single intranasal dose to healthy adult male volunteers
Study Identification Number:	**BT0604**
Sponsor	INSERM

	• Immunoglobulin, corticosteroids for systemic use or for use in the respiratory tract (e.g. nasal steroids, inhaled steroids), other immunosuppressive agents or immunostimulating agents.<br>• Pertussis vaccines.<br>• Topical nasal therapies.<br>If the volunteer needs a medication or a vaccine more than one month after inclusion in the study this will be registered and both the principal investigator and the sponsor will decide whether the volunteer can continue in the study or not.
**Vaccination schedule**	Drops administered by tuberculin syringes, one syringe containing 0.1 mL for each nostril at visit 2 with 0.1 mL of air on top of the fluid vaccine/placebo.
**Evaluation criteria**	**SAFETY (Primary evaluation)**<br>The safety evaluation criteria will be the number of subjects with the following adverse events:<br>From Day 0 (Visit 2) to Day 7 (Visit 4)<br>Solicited adverse reactions from the respiratory tract:<br>• Local AE which lead to difficulties in breathing will be considered as serious and reported to the independent data monitoring committee and sponsor.<br>• All other local AE from the respiratory tract will be recorded in the volunteer´s CRF.<br>From Day 0 to Day 28:<br>• Solicited systemic adverse events (pyrexia, headache, pain)<br>• Unsolicited respiratory tract reactions<br>• Unsolicited systemic adverse events<br>Adverse events (AE) should be reported at study visits during the first 28 days of the concerned subject.<br>Serious adverse events (SAE) should be reported as soon as possible but no later than 1 business day after detection.<br>**COLONISATION OF THE BPZE1 STRAIN IN THE NASOPHARYNGEAL MUCOSA (Secondary evaluation)**<br>The ability of the modified B. pertussis strain BPZE1 to colonise the human respiratory tract and for how long the microorganism is shedding.<br>**IMMUNOGENICITY (Secondary evaluation)**<br>Pertussis antibodies will be measured before and after vaccination at visits 2, 4, 6, 7 and 8. The number of individuals with positive IgA and IgG antibody response will be measured to the following antigens:<br>• Pertussis toxin<br>• FHA<br>• Fimbriae 2/3<br>• Pertactin<br>• Whole B. pertussis cell lysates<br>A positive antibody response after vaccination is defined as follows: At least 100% increase from pre- to post-vaccination, to at least 4 times MLD (minimum level of detection) for PT, FHA, pertactin, fimbriae 2/3 and whole B. pertussis cell lysate in the post-vaccination sample<br>For all serological assays the geometric means should be displayed for pre- and post-vaccination sera in each dosing group.<br>Sera will be tested blindly for pertussis specific IgG and IgA antibodies. Antibody levels to PT, FHA fimbriae 2/3, pertactin and whole B. pertussis cell lysate will be expressed in ELISA units (EU/mL) calibrated against reference antisera from the National Institute for Biological Standards and Controls.

**Study title**	A phase 1, single centre, dose-escalating, placebo-controlled study of a genetically modified B. pertussis strain given as a single intranasal dose to healthy adult male volunteers
**Study Identification Number:**	**BT0604**
**Sponsor**	INSERM

	Saliva samples and nasopharyngeal aspirate will be analysed for IgA antibodies against pertussis antigens.<br>Cell-mediated immunity will be analysed. The separated peripheral blood mononuclear cells will be analysed for cytokine production and memory T cell phenotypic markers after stimulation with vaccine antigens and for the presence of effector and memory B-cells.
**Statistical analysis**	Description of the number of adverse events. These analyses will describe local (respiratory tract) adverse reactions, systemic adverse events, daily temperatures and serious adverse events compared for each of the 3 vaccine groups with the control group and between the 3 vaccine groups.<br>To describe in each vaccine group pre- and postvaccination<br>• Number of responders and levels of IgG and IgA antibodies to pertussis toxin, filamentous hemagglutinin, pertactin, fimbriae and whole B. pertussis cell lysate in serum, and to IgA antibodies in saliva and nasopharyngeal aspirate.<br>• Number of responders and levels of T cell immune responses by measuring production of cytokines and T cell memory after stimulation with pertussis toxin, filamentous hemagglutinin, and pertactin (exploratory assays).<br>• Number of responders and levels of B cell immune responses as determined by the numbers of effector and memory B cells after stimulation with pertussis toxin, filamentous hemagglutinin, and pertaktin (exploratory assays).<br>For all assays the geometric means should be displayed for pre- and postvaccination samples in each vaccine group.

I.4. PROCEDURAL FLOW-CHART

**Study Visit**	**1**	**2**	**3**	**4**	**5**	**6**	**7**	**7'**	**8**
	2-6 weeks prior to visit 2	Day 0	Day 4±1	Day 7±1	Day 11±1	Day 14±1	Day 28±1	Day 45±5	5-6 mon
**Administrative Requirements:**									
Recruitment, Medical record number, Screen form,	x								
Information and study consent[a]	x								
Demographic information (age, gender, race)	x								
**Vaccination:**		x							
**Clinical Requirements:**									
Complete medical history	x								
Physical examination include. vital signs[b]	x	x	x	x	x	x	x	x	x
Interim History/Diary (AE documentation, concomitant medication)[c,d]		x	x	x	x	x	x	x	x
Randomization		x							
**Safety Labs:**									
ECG	x								
Hematology[e]	x	x		x		x	x		x
Blood chemistry[f]	x								
Screening for HIV, hepatitis B and C[g]	x								
Serum pertussis toxin IgG antibodies	x								
Urine analysis[h]	x								
Total IgE	x						x		x
Nasopharyngeal culture for B. pertussis			x	x	x	x	x[i]	x[i]	
**Immunogenicity Labs and Sample Archive:**									
Binding pertussis IgG and IgA serum antibodies		x		x		x	x		x
Binding pertussis IgG and IgA saliva antibodies		x		x		x	x		x
Binding pertussis IgG and IgA nasopharyngeal antibodies		x		x		x	x		x
B cell ELISpot		x		x[j]		x[j]	x		x
IFN-γ, IL-2 ELISpot		x				x[j]	x		x
Multicolour memory lymphoproliferative response		x					x		x
Cryo-preserved cells	x	x		x[j]		x[j]	x		x
TOTAL BLOOD VOLUME (ML)	58.5 ml	109 ml		48.5 ml		101 ml	112.5 ml		120.5 ml

(a) The ICF must be signed by the subject and investigator before vaccination with a study vaccine.

(b) At Visit 1, full physical examination (e.g. inspection of tonsils, palpation of glands, auscultation of pulm, measuring height and weight) including ECG and vital signs (oral temperature, blood pressure, heart rate, respiratory rate). For the following visits, physical exam will be restricted to general appearance, heart, lung, skin, nose, throat and vital signs.

(c) Adverse events (AE) occurring within at least 6 hours post-vaccination have to be recorded at the study centre and AE occurring from Day 0 to Day 28 have to be recorded in the Diary. I**f a vaccine-related AE is not resolved by Day 28, it must be followed up until resolution or stabilisation.**

(d) Serious adverse events (SAE) occurring from Visit 1 (screening) to Visit 8 have to be recorded at the study site In the Diary SAE will be recorded up to visit 7. If an SAE is not resolved at the last visit of the concerned subject, it must be followed up until resolution or stabilisation.

(e) Hematology (haemoglobin, total and differential WBC, RBC, platelets)

(f) Blood chemistry (potassium, calcium, sodium, creatinin, albumin, serum bilirubin, alkaline phosphatases, alaninaminotransferase (ALAT), aspartataminotransferase (ASAT), glutamyltransferase (GT), high sensitive C-reactive protein, blood glucose, thyreoidea stimulating hormone (TSH).

(g Including HIV1/HIV2, HBsAg, anti-HBc and anti-HCV.

(h) Dipstick analysis of pH, erythrocytes, leucocytes, protein, glucose, ketones and bacteria (nitrite) and the drugs cocaine, amphetamine, cannabis, morphine, benzodiazepines, and methylenedioxymethamphetamine.

(i) If positive culture after 4 weeks a new nasopharyngeal sample should be collected 2-3 weeks later for culture.

(j) Samples from the first two subjects of each group will not be collected on day 7(±1) and 14(±1).

II. Important medical procedures to be followed by the investigator

II.1. EYE EXPOSURE

The Principle Investigator (PI)/subinvestigator or delegate at the Clinical trial centre will see all staff and all volunteers who accidentally will have droplets of the vaccine in the eye. The principal investigator/ subinvestigator will see all non-trivial cases. Equipment for eye washing will be available. Protective goggles (included in universal precautions) will be worn by subject and staff when administering the vaccine.

II.2. STOPPING CRITERIA FOR DOSE ESCALATION/CONTINUATION OF STUDY

All severe and serious adverse events, whether believed to be caused by the vaccine or not, will be reported to the PI or delegate, who will contact the Independent data monitoring committee (IDMC) and the sponsor. The PI or delegate will decide whether the code should be broken for the individual case and in consultation with the sponsor make decisions about stopping the study (permanently or temporary), postponing further vaccination or continuing the study. Following criteria would be considered as stopping criteria:

- One volunteer has symptoms compatible with whopping cough and wild type pertussis is not cultured or demonstrated by PCR (and active vaccine given). In such a case, it must be suspected that BPZE1 has caused symptoms.
- One volunteer has a serious adverse event which is considered by the investigator, the members of the IDMC and the sponsor most probably caused by the investigational vaccine.
- Two volunteers have a serious adverse event, possibly caused by the investigational vaccine.

II.3. METHOD OF BLINDING AND BREAKING THE STUDY BLIND

For safety reasons the first two volunteers in each dosage group will be vaccinated with the active substance (unblind). The following 14 subjects in each dosage group study will be vaccinated in a double-blind manner. That means that all individuals involved in the administration of the vaccine and the assessment of reactogenicity of the vaccine and the subjects will be blinded. Sponsor staff involved in review/analysis of data is also unaware of the treatment assignments.
Since results of the cultivation of B. pertussis from the nasopharynx samples and the antibody determination may lead to treatment unblinding, these results will be strictly controlled within the laboratory. The access of these results outside the laboratory will be contingent on having a previous access to the randomization and therefore will only be authorized after the completion of the study. The study will be unblended after all safety and laboratory results are monitored and the data bases are locked.

EXCEPTION! If any subject has detectable vaccine strain bacteria in the nasopharyngeal sample collected 28 (±1) days after vaccination. Under such a circumstance the laboratory has to inform the investigator, who should arrange for an extra visit for collection of a new nasopharyngeal sample for control of bacterial shedding.

Set of individual codes will be held at the Karolinska hospital pharmacy (available 24 hours 7 days a week), at the manufacturer site (Innogenetics), and at the sponsor delegate site (ANRS vigilance team). The code will be broken by PI or delegate only in the case of medical events that investigator/physician in charge of the subject feels cannot be treated without knowing the identity of the study vaccine.

III. Introduction

III.1. Background and study rationale

Before introduction of general vaccination, pertussis was a typical childhood disease. The incidence in children has been drastically reduced with vaccination of infants and booster doses to school children, but B. pertussis continues to circulate even in populations with high vaccine coverage of infants and children. Pertussis in adolescents and adults is increasing, and from these age groups the organism may spread to neonates and infants too young to be vaccinated. Approximately 40 million whooping cough cases and between 200,000 and 400,000 pertussis-linked deaths are recorded each year, mostly in developing countries (WHO, 2004). Fatal cases among infants and patients with severe underlying diseases occur also in developed countries. Pertussis is one of the few infectious diseases with a high incidence also in countries with high vaccination coverage. Thus, even in countries with high vaccination coverage and 1 – 2 booster doses the herd immunity achieved does not seem to protect infants from exposure. This emphasizes the need for a new global vaccine strategy with a high degree of individual protection of neonates.

A genetically modified strain of B. pertussis has been constructed. In this strain three toxins have been inactivated or deleted. The gene for pertussis toxin, one of the major virulence factors and protective antigens, has been modified so that a nontoxic protein with preserved immunological properties is produced by the organism. This toxoïd is similar to the genetically modified toxin which had a high efficacy in a double-blind placebo-controlled trial in Italy (Greco D et al. A controlled trial of two acellular vaccines and one whole-cell vaccine against pertussis. N Engl J Med 1996;334:341-8).

The gene for dermonecrotic toxin has been deleted, so this toxin is not expressed by BPZE1, and a replacement of the ampG gene of B. pertussis by that of E. coli has decreased the production of tracheal cytotoxin to background levels.

The BPZE1 strain therefore has the potential for being a candidate for a safe nasal vaccine against pertussis, because the three major toxins are inactivated or deleted. This should make the risk for adverse events minimal. Furthermore, if the strain retains the ability to colonise the human respiratory tract it should be able to induce disease-like immune responses against several antigens: pertussis toxin, filamentous haemagglutinin, pertactin, fimbriae and whole B. pertussis cell lysates.

III.2. Benefit/risk and ethical assessment

General advantage of a modified live vaccine given intranasally over currently available injectable acellular pertussis vaccines. Effective vaccines are needed to protect young infants (from 0 to 6 months, today the most vulnerable age group), preferably after a single administration very early in life. One of the major impacts of CHILD-INNOVAC is to provide a solution to this problem. The successful outcome of this project would constitute an important milestone towards nasal vaccination of infants, possibly at birth with a novel, single-dose pertussis vaccine. The ultimate aim is to protect infants in the most vulnerable age group, before the regular vaccination schedule using already available vaccines is applied. The ultimate aim is thus not to replace current vaccination schedules with available vaccines, but to add a first nasal vaccination to protect very early in life. Classical vaccination schedules would then constitute important booster immunisations. Early immune imprinting by infection or vaccination has a long-term impact on immunity. Current vaccination with acellular pertussis vaccines has been shown to bias the immune responses towards Th2 type responses, or to retard the Th1 type responses in children, even towards unrelated antigens and polyclonal immune stimulation. Nasal vaccination with BPZE1 is expected to promote the development of Th1 type responses, as natural infection with B. pertussis induces strong levels of IFN-γ towards B. pertussis antigens in very young children. The induction of Th1 type responses by nasal vaccination with BPZE1 has already been shown in infant mouse models. In addition to Th1 type responses nasal vaccination with BPZE1 also induces antibodies against the major protective antigens. It is therefore expected that both protective immune parameters (antibodies and Th1 type responses) will be induced in infants vaccinated with BPZE1.

Pertussis is a world-wide disease, present both in developing countries and in the developed world, including in all European countries. The development of an effective nasal vaccine against this disease will thus have a planet-wide impact. It will improve the health of infants throughout the world. Because of the global importance of the disease, the market of the product developed through the CHILD-INNOVAC project is essentially world-wide. The final but distant goal would be to vaccinate all children world-wide with a live nasal vaccine similar to the goal for another live bacterial vaccine, BCG. After neonatal vaccination with a nasal vaccine, it is most likely that vaccination will have to continue with the currently available injectable acellular vaccines (or with whole cell vaccines in poor countries).

Environment risk assessment of the live attenuated pertussis vaccine BPZE1.

Detailed information about the genetically modified organism BPZE1 is available in the Environment risk assessment (Appendix 1 Environment risk assessment of vaccine used in the Child Innovac 1/Miljöriskanalys för vaccin ingående i Child Innovac 1). The attenuated strain of Bordetella pertussis named BPZE1 was engineered by eliminating or genetically detoxifing three B. pertussis toxins, pertussis toxin, dermonecrotic toxin and tracheal cytotoxin (Mielcarek et al., Live Attenuated B. pertussis as a Single-Dose Nasal Vaccine against Whooping Cough. 2006. PLoS Pathogens).

The genetic modifications in BPZE1 strongly increase the in vivo and in vitro safety:
- No lethal effect of BPZE1 was observed in mice, even after nasal administration of 10^6 colony forming units (cfu). The histological analysis data showed a decreased colonization and proliferation power of the BPZE1 cells in the trachea and no weight reduction was observed after nasal administration of the BPZE1-strain.
- Dissemination of BPZE1 was not observed in mice with severe immunodeficiency.
- No cell toxicity of the pneumocyte and macrophage cell lines was observed after incubation with the BPZE1-strain (in vitro safety test).
- The double nucleotide mutation in the substrate binding and the active site of the pertussis toxin (PTX) results in a strong reduction of the enzyme activity.
- The replacement of the B. pertussis *ampG* gene by the *E. coli ampG* gene results in an over 95% reduction in release of the tracheal cytotoxin (TCT) in the medium.
- The dermonecrotic toxin (DNT) is not expressed in the BPZE1 strain.

Based on these data the BPZE1 has been classified as a BSL 1 organism by the French authorities (Appendix 2).

There is no known animal vector or reservoir for B. pertussis. The genetic modifications (replacement of the ampG gene, deletion of the dermonecrotic toxin and the mutations of the pertussis toxin) are not expected to alter the host range of B. pertussis BPZE1 compared to the wild type *B. pertussis* bacteria.

The BPZE1 is not invasive and has no selective advantage in the environment. The potential for exchange of genetic material is virtually inexistant, since B. pertussis does not harbor plasmids or conjugative transposons. In addition, B. pertussis Tohama I (background used for the BPZE1 strain) does not harbor intact prophage genomes and is therefore incapable of producing functional phage particles.

In summary, the risk assessment for this study shows a very low risk for potential environmental impact associated with administering the BPZE1 to volunteers.

Volunteers risk assessment of the live attenuated pertussis vaccine BPZE1.

B. pertussis colonization is strictly limited to respiratory epithelium without extrapulmonary dissemination of the bacteria, which naturally excludes systemic bacteremia of the BPZE1 strain.

B. pertussis is spread by aerosol formed by coughing of infected persons. The coughing is induced by the tracheal cytotoxin, which is more than 95% reduced in BPZE1. The BPZE1 strain is not expected to induce coughing therefore the rate of transmission will be highly unlikely. The bacteria have fastidious growth requirements and have limited survival time outside the human body.

B pertussis has not been shown to be allergenic in any preclinical or clinical studies to date. On the contrary the BPZE1 has been demonstrated to protect against airway inflammations induced by allergens or viral infections in a mouse model. The BPZE1 has also been shown to protect against infection with wild type B pertussis infection already 3 hours after immunization in a mouse model.

The BPZE1 vaccine will be administered nasally via tuberculin syringes to healthy adult male volunteers under strictly controlled conditions at the Karolinska Trial Alliance phase I/II unit. To minimize the risk of transmission the volunteers will stay at the study centre for 6 hours after administration of the vaccine. In addition males with frequent contact with infants below one year of age or individuals with immunodeficiency will be excluded from participation in the study. The attenuated BPZE1 bacteria are expected to colonize the upper respiratory tract similarly to the wild-type B. pertussis. Shedding of live organism will be followed in nasopharyngeal washings performed at various intervals from the day of administration until the end of the study. Chronic carriage of B. pertussis has not been reported and is therefore not expected.

To avoid accidental exposure actions should be taken to minimize generation of aerosols, since the bacteria is strictly a respiratory tract organism. The volunteers and the staff members should wear eye-protective glasses during the vaccination Persons handling the BPZE1 bacteria should wear gloves and must wash their hands with a suitable disinfecting soap before touching their skin and eyes. In case of transmission to other humans, accidentally exposed, an efficient treatment against B. pertussis is commercially available and is based on administration of erythromycin. BPZE1 has been shown to be sensitive to erythromycin.

In summary, the risk assessment for this study shows a very low potential risk for the volunteers and impact associated with administering the BPZE1.

IV. Study objectives

IV.1. SAFETY (PRIMARY)

To determine

- general safety, i. e. general well-being of the volunteers and any symptoms felt by the volunteers with onset within one month after vaccine administration.
- vital signs: Blood pressure, heart rate, respiratory rate, oral temperature.
- abnormalities in the following laboratory data: Haemoglobin (i.e. B-haemoglobuline), total and differential white blood cell count (i.e. granulocytes**:** neutrophils, eosinophils**,** and basophils**,** non-granulocytes: lymphocytes **and** monocytes) platelets (thrombocytes) and red blood cells (RBC).
- specific side effects: Local symptoms from the respiratory tract: Sneezing, swollen nose, cough, bleeding from the nose, pain or other symptoms from the ear, symptoms from the eyes (redness, secretion).

IV.2. ATTACHMENT OF THE BPZE1 STRAIN TO THE NASOPHARYNGEAL MUCOSA (SECONDARY)

To describe in each vaccine group the time interval for detection of colonizing BPZE1 bacteria.

IV.3. IMMUNOGENICITY (SECONDARY)

To describe in each vaccine group

- IgG and IgA serum antibody concentrations to pertussis toxin, filamentous hemagglutinin, pertactin, fimbriae and whole B. pertussis cell lysate.
- IgA antibodies to the same 5 antigens in saliva and nasopharyngeal aspirate.
- T cell immune responses by measuring pre- and postvaccination production of cytokines and T cell memory after stimulation with pertussis toxin, filamentous hemagglutinin, and pertactin.
- B cell immune responses as determined by the numbers of effector and memory B cells after stimulation with pertussis toxin, filamentous hemagglutinin, and pertactin.

V. Selection of volunteers

V.1. INCLUSION CRITERIA

Subject will be included in the study if he meets all the following criteria:

- Healthy male born between 1979 and 1991 who have not experienced clinical pertussis during the past 10 years (lab verified) and who have not been vaccinated with any pertussis vaccine.
- The subject must be willing to give written informed consent to participate in all stages of the study (but can withdraw, see below).
- Subject shall be able to attend all scheduled visits and to understand and comply with the study procedures (e.g. able to read and write Swedish).

V.2. EXCLUSION CRITERIA

If any of the following criteria are met, the subject must not be included in the study:

1. Individuals with pertussis toxin serum IgG antibodies ≥ 20 international units/mL.
2. Blood pressure after resting ≥ 150/90 mmHg.
3. Heart rate after resting ≥ 80 bpm.
4. Respiratory rate after resting ≥ 20/minute.
5. Unwillingness to refrain from the use of nicotine products from screening through day 28.
6. Use of narcotic drugs and/or a history of drug/alcohol abuse whitin the past 2 years prior to screening
7. The subject has donated blood or suffered from blood loss of at least 450 ml (1 unit of blood) within 60 days prior to screening or donated plasma within 14 days prior to screening.
8. Receipt of immunoglobulin, blood derived products, systemic corticosteroids or other immunosuppressant drugs within 90 days prior to day 0.
9. Use of corticosteroids in the respiratory tract (e.g. nasal steroids, inhaled steroids) 30 days prior to day 0.
10. Use of herbal medications or dietary supplements within 7 days prior to day 0 at the discretion of the investigator. Unwillingness to refrain from herbal medications or dietary supplements within 30 days after day 0 at the discretion of the investigator.
11. Receipt of a vaccine within the last 30 days prior to day 0 or planned vaccination within the next 30 days after day 0.
12. Evolving encephalopathy not attributable to another identifiable cause within 7 days of administration of a previous dose of any vaccine.
13. Known hypersensitivity to any component of the study vaccine.
14. Current participation in any other clinical trial or participation (and during the whole study) in any clinical trial in the previous 3 months prior to day 0.
15. Inability to adhere to the protocol, including plans to move from the area.
16. Family history (first degree) of congenital or hereditary immunodeficiency.
17. Infection with HIV, hepatitis B or C.
18. Any medical condition which, in the opinion of the investigator, might interfere with the evaluation of the study objectives.
19. Clinically significant abnormal laboratory values at the discretion of the investigator.
20. Person in frequent contact with children less than 1 year of age (father, childcare worker, nurse, etc…) or residence in the same household as persons with known immunodeficiency including persons on immunosuppressive treatment.

V.3. TEMPORARY CONTRAINDICATIONS

Reschedule vaccination if

- Febrile illness and/or oral temperature ≥ 38.0°C at the time of vaccination.
- Ongoing rhinitis and/or rhinoconjunctivitis.

VI. Study plan and design

VI.1. Planned Study Calendar

FVFS	*August 2010*
FVLS	*December 2010*
LVLS	*June 2011*
Laboratory analysis	*August 2010 – December 2011*
Statistical analysis	*August 2011– January 2012*
Clinical study report	*February 2012*

The end of the trial will be the last visit of the last subject.

VI.2. General Study Plan and Design

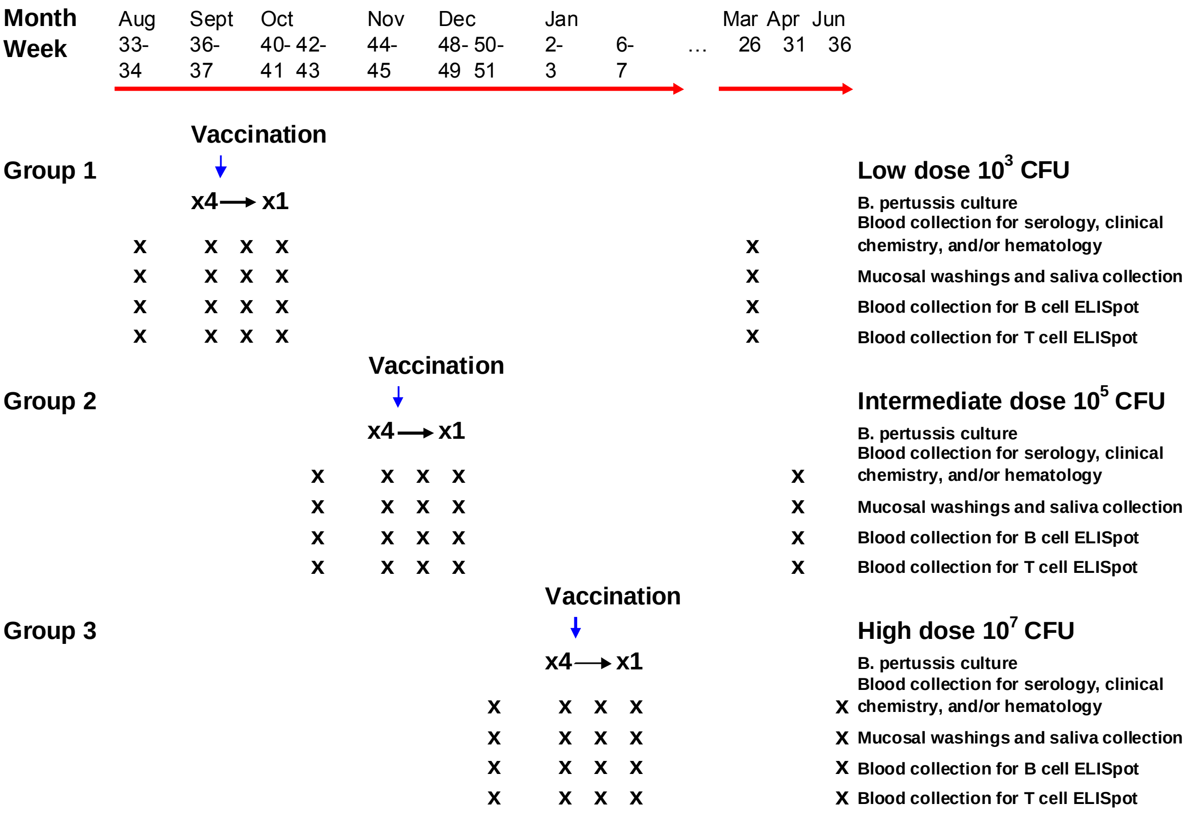

Inclusion of 1+1+2+3+4+5 group

The study is a dose-escalating study with a control group.

The volunteers will be recruited in a step-wise fashion with 16 individuals in each of three groups.

- Group 1: 12 individuals will be vaccinated once intranasally with 1,000 colony forming units (cfu) of the organism. 4 individuals will get placebo (the diluent alone).
- Group 2: 12 individuals will be vaccinated once intranasally with 100,000 colony forming units (cfu) of the organism. 4 individuals will get placebo (the diluent alone).
- Group 3: 12 individuals will be vaccinated once intranasally with 10,000,000 colony forming units (cfu) of the organism. 4 individuals will get placebo (the diluent alone).

The administration of vaccine or diluent alone will be performed in a double-blind fashion with administration of coded vials (see below) except that the first 2 subjects in each group will receive active substance.

The volunteers in each group will be included sequentially in the study and vaccinated according to the following schedule

The two first individuals will be vaccinated with active substance. The following 14 individuals will be randomized as described in VI.4 *“Subject Number and randomisation procedure”*.

- Day 1: 1 individual
- Day 2: 1 individual
- Day 3: 2 individuals with 3-4 hours interval
- Day 4: 3 individuals with 2 hours interval
- Day 5: 4 individuals with 1 hour interval
- Day 6: 5 individuals with 1 hour interval

Group 1 volunteers will be followed up during at least 4 weeks before the group 2 volunteers become vaccinated. Group 3 volunteers will be vaccinated when the group 2 volunteers have been followed for at least 4 weeks.

Interim safety meetings with the Independent Data Monitoring Committee and the sponsor will be held before administering the next higher dose.

VI.3. STUDY VISITS

This study will include 8 or if required 9 study visits. An overview of all study visits with interviews, clinical examinations and samples to be taken are presented in I.4 “*Procedural Flow Chart*”.

- Visit 1, recruitment visit 2-6 weeks before vaccination: medical history, physical examination and collection of blood and urine sample and ECG
- Visit 2, inclusion visit, 2-6 weeks after visit 1: physical examination, collection of blood, saliva and nasopharyngeal sample and vaccination with vaccine or placebo
- Visit 3, 4±1 days after visit 2: physical examination, safety follow up of vaccination and nasopharyngeal sample
- Visit 4, 7±1 days after visit 2: physical examination, safety follow up of vaccination and collection of blood, saliva and nasopharyngeal sample
- Visit 5, 11(±1) days after visit 2: physical examination, safety follow up of vaccination and nasopharyngeal sample
- Visit 6, 14±1 days after visit 2: physical examination, safety follow up of vaccination and collection of blood, saliva and nasopharyngeal sample
- Visit 7, 28 (- 1; +7) days after visit 2: physical examination, safety follow up of vaccination and collection of blood, saliva and nasopharyngeal sample
- Visit 7’, 45(±5) days after visit 2: nasopharyngeal sample in volunteers in which BPZE1 bacteria were still detectable on day 28±1
- Visit 8, 5-6 months after visit 2: physical examination, safety follow up of vaccination and collection of blood, saliva and nasopharyngeal sample

VI.3.1. First contact

The clinical trial site will either find the healthy volunteers in the Karolinska Trial Alliance own database (5000 healthy volunteers) or by advertising in the free papers or on the face book website.

All subjects will have a personal contact by phone and a screening visit can be booked.

Written information about the study will be sent to the potentially healthy volunteers before they come to their first visit at the clinic.

VI.3.2. Visit 1, screening 2-6 weeks before vaccination

During this visit the investigator will:

1. Verify the subject's eligibility by reviewing inclusion and exclusion criteria.
2. Inform the subject about the study and obtain a signed Informed Consent Form (ICF). ICF should be dated and signed by the volunteer and the investigator before any study related activities take place.
3. Assign a screening number to the subject (refer to section VI.4. "Screening *Number*").
4. Collect information regarding the subject's demography. Record the information in the CRF.
5. Collect information regarding the subject's personal and medical history and concomitant treatment as defined in section VII.1. "*Prior medications and vaccination*". Record the information in the CRF.
6. Perform a full physical examination including ECG and measure
   a) oral temperature
   b) blood pressure
   c) heart rate
   d) respiratory rate
   e) height
   f) weight

   and record the information in the subject's CRF
7. Collect blood samples as described in section XII.3. "*Blood samples*". Complete the appropriate document provided in the investigator or delegate Site file.
8. Collect urine samples as described in section XII.6. *"Urine samples".* Complete the appropriate document provided in the investigator or delegate Site file.

VI.3.3. Visit 2, day 0, vaccination

During this visit the investigator or delegate will:

1. Verify the subject's eligibility by reviewing inclusion and exclusion criteria.
2. Confirm that a signed ICF is on file for the subject.
3. Assign a "subject number" to the subject (refer to section VI.4. "*Subject Number*").
4. Perform a limited physical examination and measure vital signs (oral temperature, blood pressure, heart rate and respiratory rate) and record the information in the subject's CRF.
5. Check of temporary contra-indications to vaccination.
6. Collect blood samples as described in section XII.3. "*Blood samples*". Complete the appropriate document provided in the investigator or delegate Site file.
7. Collect saliva samples as described in section XII.4. "*Saliva samples*". Complete the appropriate document provided in the investigator or delegate Site file.
8. Collect nasopharyngeal aspirate as described in section XII.5. "*Nasopharyngeal samples*". Complete the appropriate document provided in the investigator or delegate Site file.
9. Record the allocated randomization number in the medical journal (source document).
10. Administer the study vaccine assigned to the subject by the investigator or delegate according to the randomization *described in section VI6.4 'Screening number and Randomization procedure'*.

Vaccination should be administered intranasally by two tuberculine syringes, one to each nostril. The air cushion is not removed after the extraction with the tuberculine syringe. The fluid and the air cushion are pushed slowly out the tuberculine syringe to ensure the administration of total extracted volume (100 µL) to the nostril of the volunteer. In case of volunteer sneezing or blowing nose immediately after vaccination this should be recorded in the subject's CRF, but a new vaccine dose should not be given.

The staff and the volunteer should adhere to the safety instructions described in the Standard operating procedure.

11. Observe the subject for 6 hours after vaccination for immediate adverse events and record any adverse event.
12. Give the subject the Diary and an oral thermometer.
13. Inform the subject to record in the Diary.
    a. Any respiratory tract adverse reaction occurring until Visit 7 and to specify the intensity of the reaction using definitions given in the Diary.
    b. Any other medical event occurring until Visit 7 and to specify the intensity of the reaction using definitions given in the Diary
    c. The temperature as measured orally with the provided thermometer daily in the evening from day 0 until day 14. In case of feverish feeling from Day 15 to Visit 7, temperature should be taken and monitored in Diary. If the temperature is taken more than once during a day the highest temperature of the day should be recorded in the Diary.
    d. Any medication taken from day 0 until Visit 7.
14. Inform the subject to
    a. Immediately contact the investigator or delegate in case of any serious adverse event and/or any visit to another physician that may occur at any time between visits.
    b. Complete the whole Diary and to bring Diary back at next visit.
15. Arrange for an appointment for visit 3 (4(±1) days after visit 2)

VI.3.4. Visit 3, 3-5 days after visit 2

During this visit the investigator or delegate will:

1. Collect and review the Diary. Check for any adverse event to the vaccination, interview on <u>adverse reactions</u> from the respiratory tract and systemic adverse events that might have occurred since visit 2, or any adverse event corresponding to the definition of a serious adverse event (refer to Section XIII. "*Recording and reporting of adverse events*), and check for any concomitant medication.
2. Perform a limited physical examination and measure vital signs (oral temperature, blood pressure, heart rate and respiratory rate) and record the information in the subject's CRF.
3. Collect nasopharyngeal aspirate as described in section XII.4. "*Nasopharyngeal samples*". Complete the appropriate document provided in the investigator or delegate Site file.
4. Arrange for an appointment for visit 4 (7±1 days after visit 2).

VI.3.5. Visit 4, 7±1 days after visit 2

During this visit the investigator or delegate will:

1. Collect and review the Diary. Check for any adverse event to the vaccination, *interview on adverse reactions from the respiratory tract and systemic adverse events that might have occurred since visit 3, or any adverse event corresponding to the definition of a serious adverse event (refer to Section XIII "Recording and reporting of adverse events), and check for any concomitant medication.*
2. Perform a limited physical examination and measure oral temperature, blood pressure, heart rate,

respiratory rate and record the information in the subject's CRF.

3. Collect blood samples as described in section XII.3. "*Blood samples*". Complete the appropriate document provided in the investigator or delegate Site file.
4. Collect saliva samples as described in section XII.4. "*Saliva samples*". Complete the appropriate document provided in the investigator or delegate Site file.
5. Collect nasopharyngeal aspirate as described in section XII.5. "*Nasopharyngeal samples*". Complete the appropriate document provided in the investigator or delegate Site file.
6. Arrange for an appointment for visit 5 (11±1 days after visit 2).

VI.3.6. Visit 5, 11±1 days after visit 2

During this visit the investigator or delegate will:

1. Collect and review the Diary. Check for any adverse event to the vaccination, interview on adverse reactions from the respiratory tract and systemic adverse events that might have occurred since visit 4, or any adverse event corresponding to the definition of a serious adverse event (refer to Section XIII. "*Recording and reporting of adverse events*"), and check for any concomitant medication.
2. Perform a limited physical examination and measure oral temperature, blood pressure, heart rate, respiratory rate and record the information in the subject's CRF.
3. Collect nasopharyngeal aspirate as described in section XII.5. "*Nasopharyngeal samples*". Complete the appropriate document provided in the investigator or delegate Site file.
4. Arrange for an appointment for visit 6 (14±1 days after visit 2).

VI.3.7. Visit 6, 14±1 days after visit 2

During this visit the investigator or delegate will:

1. Collect and review the Diary. Check for any adverse event to the vaccination, interview on adverse reactions from the respiratory tract and systemic adverse events that might have occurred since visit 5, or any adverse event corresponding to the definition of a serious adverse (refer to Section XIII. "*Recording and reporting of adverse events*"), and check for any concomitant medication.
2. Perform a limited physical examination and measure oral temperature, blood pressure, heart rate, respiratory rate and record the information in the subject's CRF.
3. Collect blood samples as described in section XII.3. "*Blood samples*". Complete the appropriate document provided in the investigator or delegate Site file.
4. Collect saliva samples as described in section XII.4. "*Saliva samples*". Complete the appropriate document provided in the investigator or delegate Site file.
5. Collect nasopharyngeal aspirate as described in section XII.5. "*Nasopharyngeal samples*". Complete the appropriate document provided in the investigator or delegate Site file.
6. Arrange for an appointment for visit 7 (28 (-1: +7) days after visit 2)

VI.3.8. Visit 7, 28 (-1: +7) days after visit 2

During this visit the investigator or delegate will:

1. Collect and review the Diary. Check for any adverse event to the vaccination, interview on adverse reactions from the respiratory tract and systemic adverse events that might have occurred since visit 6, or any adverse event corresponding to the definition of a serious adverse event (refer to Section XIII. "*Recording and reporting of adverse events*"), and check for any concomitant medication.
2. Perform a limited physical examination and measure oral temperature, blood pressure, heart rate, respiratory rate and record the information in the subject's CRF.
3. Collect blood samples as described in section XII.3. "*Blood samples*". Complete the appropriate

document provided in the investigator or delegate Site file.

4. Collect saliva samples as described in section XII.4. "*Saliva samples*". Complete the appropriate document provided in the investigator or delegate Site file.
5. Collect nasopharyngeal aspirate as described in section XII.5. "*Nasopharyngeal samples*". Complete the appropriate document provided in the investigator or delegate Site file.
6. Arrange for an appointment for visit 8 (5 - 6 months after visit 2).

VI.3.9. Visit 7', 45±5 days after visit 2

In case the nasopharyngeal aspirate contains detectable vaccine strain bacteria on day 28 the laboratory contacts the clinical investigator or delegate, who contacts the subject for a new visit.

During this visit the investigator or delegate will:

1. Collect information on any adverse event to the vaccination, interview on adverse reactions from the respiratory tract and systemic adverse events that might have occurred since visit 7, or any adverse event corresponding to the definition of a serious adverse event (refer to Section XIII. "*Recording and reporting of adverse events*"), and check for any concomitant medication.
2. Perform a limited physical examination and measure oral temperature, blood pressure, heart rate, respiratory rate and record the information in the subject's CRF.
3. Collect nasopharyngeal aspirate as described in section XII.5. "*Nasopharyngeal samples*". Complete the appropriate document provided in the investigator or delegate Site file.

VI.3.10. Visit 8, 5-6 months after visit 2

During this visit the investigator or delegate will:

1. Collect information on any adverse event to the vaccination, interview on adverse reactions from the respiratory tract and systemic adverse events that might have occurred since visit 7, or any adverse event corresponding to the definition of a serious adverse event (refer to Section XIII. "*Recording and reporting of adverse events*"), and check for any concomitant medication.
2. Perform a limited physical examination and measure oral temperature, blood pressure, heart rate, respiratory rate and record the information in the subject's CRF.
3. Collect blood samples as described in section XII.3. "*Blood samples*". Complete the appropriate document provided in the investigator or delegate Site file.
4. Collect saliva samples as described in section XII.4. "*Saliva samples*". Complete the appropriate document provided in the investigator or delegate Site file.
5. Collect nasopharyngeal aspirate as described in section XII.5. "*Nasopharyngeal samples*". Complete the appropriate document provided in the investigator or delegate Site file.
6. Complete the End of study module in the CRF.

VI.4. SCREENING NUMBER AND RANDOMISATION PROCEDURE

Subject

At visit 1 after a signed and dated Informed Consent Form has been obtained from the subject, each subject will be assigned a **unique screening number** (USN) consisting of 3 digits (for example, 001) and the intitials of the subject consisting of 3 letters (for example ATA for Anders Tomas Andersson). In case of no middle name there should be a hyphen in the middle (for example S-S for Sven Svensson).

If the consent to participate in the study is withdrawn before randomisation, this screening number will be kept and not reassigned to another subject, for any reason.

Randomisation procedure and blinding

Vaccine and placebo will come in identical, coded vials.

The vials will be coded with group number and subject number: group1: 101 – 116, group 2: 217 – 232 and group 3: 333 – 348, for the respective groups described above under point VI.2 "*General study plan and design*".

The vials will be opened consecutively and the randomisation number given to the volunteer will be recorded both in the CRF and the medical record (source data).

The list with the codes/information if a vial contains vaccine or placebo will be kept hidden from everyone involved in the vaccination or follow-up of the volunteers .The list of codes will also be kept hidden from the investigators.

The decision whether the code shall be broken for an individual volunteer or all participants will be made by the PI or delegate in consultation with the sponsor as appropriate.

An identical series of back up vials will be generated for each group. These vials will be kept and stored in a separate freezer at the clinical trial site.

VII. Prior and Concomitant medications and Non-study vaccines

VII.1. Prior medications and vaccination

All prior medications and vaccinations will be asked for and documented (in subjects' medical record) at visit 1 and updated at visit 2 as indicated below:

The following therapies must be excluded prior to the time of the vaccination (Visit 2):

- Receipt of immunoglobulin, blood derived products, systemic corticosteroids or other immunosuppressant drugs within the previous 90 days and receipt of corticosteroids for use in the respiratory tract (e.g. nasal steroids, inhaled steroids) within 30 days prior to Visit 2.
- Non-study vaccines 30 days prior to Visit 2

All medications received by the subject within 7 **days** prior to Visit 2 (Day 0) should be recorded in the CRF on page "*Concomitant medications*". The trade name, route, dose, start date, stop date and indication will be documented.

VII.2. Concomitant medications and Non-study vaccines

Any administration of concomitant medications or non-study vaccines after the Visit 2 (Day 0) will be recorded in the Diary by the subject and will be reported in the CRF by the investigator or delegate according to the following rules:

For **medications**, the trade name, route, dose, start date, stop date and indication should be recorded in the CRF on page "*Prior and Concomitant medications*".

For **non-study vaccines**, the trade name, injection side, injection site and date of vaccination should be recorded in the CRF on page "*Concomitant non-study vaccines*".

The principal investigator will decide whether the volunteer can continue in the study or not.

VII.3. Not allowed medications & vaccines

Not allowed medications and vaccines during the first month after vaccination

The following therapies should be avoided during the first month after vaccination. If it is necessary to give a medication or a vaccine, this shall for obvious reasons be permitted. All medications and vaccines will be documented in the CRF. The volunteer will be followed as pre-planned for adverse events. The immunological part of the study can also continue, but the results from the volunteer must be reported separately under the following conditions:

- Antibacterial agents with an effect on B. pertussis, notably macrolides, azitromycin, tetracyklines, trimetoprim-sulphamethoxazole, quinolones.
- Immunoglobulin, corticosteroids for systemic use or for use in the respiratory tract (e.g. nasal steroids, inhaled steroids), other immunosuppressive or immunostimulating agents.
- Topical nasal therapies.
- Pertussis vaccines. No other vaccine is known to cross-react with pertussis vaccines. Therefore the volunteer can continue in the study if other vaccines are given. Vaccines which can be anticipated, though only rarely, are tetanus vaccine in case of a wound, influenza vaccines in case of an outbreak of influenza, vaccines intended for travellers in case the volunteer will perform a visit in other countries with short notice. If a journey is known and vaccines are planned already at the screening visit, the volunteer will not be included in the study.

If the volunteer needs a medication or a vaccine more than one month after inclusion in the study this will be registered and the principal investigator will decide whether the volunteer can continue in the study or not. But as far as possible the subject must be followed as scheduled.

VIII. Withdrawals/study termination

Subjects may be withdrawn from the study and/or the study vaccination schedule for the following reasons:

- At their own request
- On PI decision
- The Independent data monitoring committee (IDMC), PI and the sponsor have the right to terminate the study at any time if adverse events which may be related to the study vaccine occur.

In all cases, the reason for withdrawal can if available be recorded in the CRF. The subject must be followed up to establish whether the reason was an adverse event, and, if so, this must be reported in accordance with the procedures in Section XIII. *"Recording and reporting of Adverse Events*". The adverse event should be followed-up by the investigator until its complete resolution or stabilisation.

As far as possible, all examinations scheduled for the final study day (at 5-6 months) must be performed on all subjects who received the investigational product (including withdrawn subjects) but did not complete the study according to protocol.

The investigator must make every effort to contact subjects lost to follow-up. This information will be documented in the CRF. Withdrawn subjects will not be replaced.

IX. Follow up of volunteers

After successful completion and evaluation of the present study (Child Innovac I) the volunteers will be asked if they would like to participate in a next study, in which they will become vaccinated with one of the commercially available acellular pertussis vaccine after due approval from the Medical Product Agency and the Local Ethics Committee.

X. Investigational Medicinal Products i.e. Study vaccine

X.1. IDENTITY OF STUDY VACCINE

Live attenuated Bordetella pertussis BPZE1 bacteria

X.2. SUPPLY OF STUDY VACCINE

Formulated attenuated Bordetella pertussis BPZE1 at 3 different doses (Colony Forming Unit) (see Investigational Medicinal Product Dossier)

Liquid formulation of live attenuated Bordetella pertussis BPZE1 bacteria in phosphate buffered saline (containing sodium chloride and potassium chloride) and 5 % saccharose at three different colony-forming units (see Investigational Medicinal Product Dossier)

X.3. DOSES AND TREATMENT REGIMENS

A dose escalation placebo controlled study will be performed, starting with a low dose (1,000 cfu), followed by a medium dose (100,000 cfu), followed by a high dose (10,000,000 cfu) + placebo consisting of the formulation buffer (phosphate buffered saline (containing sodium chloride and potassium chloride) and 5 % saccharose).

Vaccine or placebo will be given as single administrations by nasal application in 0.1 mL in each nostril. The dose of BPZE1 active ingredient or placebo will be administered to the volunteer within 2 hours after thawing and leaving the freezer.

The first 2 subjects of each dose/group will receive the live attenuated Bordetella pertussis BPZE1.

X.4. LABELLING AND PACKAGING OF STUDY VACCINE

Vials of vaccine and placebo are provided in cryotubes (polypropylene) of 1.8 mL. The vial closure system is a polypropylene screw cap (see Information Manufacturing Drug Product).

Vaccine and placebo will come in identical vials.

Per dose/group, the vials with the vaccine or placebo will be identically coded. Only the group/subject number on the vials will be different.

Per subject, the vials (2 vials for administration and one extra as reserve) will be packed in a small plastic bag (outer package).

The bags with the vaccine vials or placebo vials will receive an identical label. Only the group/subject number on the bag will be different.

The bags containing the vaccine or the placebo will be packed in per group/dose in 2 boxes of 8 bags.

Primary labels for the cryovials and secondary labels for the plastic bags are designed.

Primary labels for the 3 different groups in the BPZE1 Drug Product Phase I study

Group 1: Low dose	Group 2: Middle dose	Group3: High dose
Trial code: BT06-04	Trial code: BT06-04	Trial code: BT06-04
BPZE1 $5x10^2$ CFU-100µl	BPZE1 $5x10^4$ CFU-100µl	BPZE1 $5x10^6$ CFU-100µl
Nasal administration	Nasal administration	Nasal administration
Batch: XXXXXX	Batch: XXXXXX	Batch: XXXXXX
Exp.: DDMMYYYY	Exp.: DDMMYYYY	Exp.: DDMMYYYY
Store below -60°C	Store below -60°C	Store below -60°C
For clinical use only	For clinical use only	For clinical use only
Sponsor INSERM	Sponsor: INSERM	Sponsor: INSERM
Tel: +33 (0)1 44 23 60 90	Tel: +33 (0)1 44 23 60 90	Tel: +33 (0)1 44 23 60 90
Patient No: 1XY*	Patient No: 2XY*	Patient No: 3XY*

*: XY varies from 1 to 16 for group 1, from 17 to 32 for group 2 and from 33-48 for group 3.

Label for the outer package for the BPZE1 Phase I study

INSERM – ISP- 75013 Paris – FRANCE / +33 (0)1 44 23 60 90

Clinical trial: BT06-04 **ChildInnovac**

Nasal drops Administration: nasal only

Production: Innogenetics - Belgium

Investigator: Karolinska University Hospital, Nabil Al-Tawil

BPZE1 xx CFU-100µl **Batch n°:** xxxxxx

Exp.: xx/xx/xxxx Storage below – 60°C

Genetic Modified Organism

Use under medical surveillance

For clinical trial use only

Group/ Patient N°: XXY

BPZE1 xx CFU is 5×10^2 CFU for group 1, 5×10^4 CFU for group 2 and 5×10^6 CFU for group3.

Group/ Patient N° is 101 to 116 for group 1, 217 to 232 for group 2 and 333 to 348 for group3.

X.5. SHIPMENT OF STUDY VACCINE

The 2 boxes containing the vaccine and placebo will be shipped on dry ice in boxes with temperature charts directly to the clinical trial site. The backup vials will be shipped in a second shipment. The reason for not shipping the study vaccine to the local pharmacy is to minimize the risk of breaking the cold chain (see below). The two series will at the clinical centre be stored in two separate freezers below -60°C.
The boxes will be shipped by the company Movianto Belgium Dirk Raes NV, Moerstraat 48-50, B-9031 Gent (Drongen), Belgium; (Phone +32 9 280 74 40; Fax +32 9 280 74 44).

- At the moment of receipt, check the presence of dry ice and temperature charts
- Care must be taken to minimize the exposure to room temperature :remove the material out of the dry ice box in front of the freezer with set point below -60°C

X.6. STORAGE OF STUDY VACCINE

Frozen storage, below -60°C at the clinical trial site.

It is critical that the vaccine is kept frozen at -60°C until the thawing just prior to vaccination to maintain the viability of the pertussis BPZE1 bacteria. The storage of the BPZE1 at room temperature should not be longer than 4 hours. It is recommended that the thawed BPZE1 solution is administered within 2 hours after leaving the freezer. Once the product has been thawed, it should be used. It is not allowed to freeze the product after it was thawed and to use it afterwards.

The vaccine will be kept in a locked freezer with alarm and continous temperature monitoring (temperature charts). Back up vials will be kept in a separate freezer with alarm and continous temperature monitoring (temperature charts).

- *Remember that samples which were thawed should never be refrozen.*

X.7. HANDLING AND ADMINISTRATION OF STUDY VACCINE TO PARTICIPANTS

It is critical that the vaccine is kept frozen below -60°C until the thawing just prior to vaccination to maintain the viability of the *B. pertussis* BPZE1 bacteria. Initial stability studies have shown that the BPZE1 Drug Product is very sensitive to freeze-thaw cycles and therefore the following actions must be taken to eliminate the risk of breaking the cold chain before administrating study drug. The following handling procedure is recommended for having a delivery of a uniform dose of BPZE1 colony forming units:

- Take the 3 cryovials out of the plastic bags immediately after removal out the freezer
- Put the cryovials immediately in a "cryovial rack", make sure that the cryovial is in vertical position
- Check that the cryovials are not damaged.
- Leave cryovials for about 30 min at room temperature before administration
- ***Do not invert or shake the cryovials before use***
- Attention: 1 cryovial was added as a reserve vial. Do not use this reserve vial without approval of the responsible person.
- Extract slowly the total volume of one (1) cryovial by placing a 1 mL- tuberculine syringe on the bottom of the cryovial.

- ***NB! Do not remove the air present in the syringe***
- Administer to one nostril the whole content of the syringe by slowly pushing the fluid and the air cushion out of the tuberculine syringe.
- Take a new 1-mL tuberculine syringe and repeat the extraction and administration procedure for the second cryovial
- Administer the whole content of the syringe to the other nostril.

Thawed samples, which were not administered (delivered) within the maximum time of exposure to room temperature (max. 4 hours), may "NOT" be used and may "NOT" be refrozen below -60°C. These cryovials must be destroyed according to the applicable procedure.

X.8. ACCOUNTABILITY OF STUDY VACCINE

The investigator or delegate is responsible for the management of study vaccine available at the investigational site and will have to maintain accountability records of the study vaccine. The accountability will include quantity of study vaccine delivered to the site, study vaccine inventory at the site (stock), study vaccine administered to subjects, study vaccine wasted/lost and unused product destroyed (see X.9. below).

The **"*IMP Accountability Form*"** must be completed. The monitoring staff will verify the investigational site's product accountability records against the record of administered study vaccine in the CRF.

X.9. DESTRUCTION OF STUDY VACCINE

All vials (empty, unused, unusable) must be kept at the investigator site until the reconciliation has been performed by the clinical Monitor.

After authorisation by the sponsor the empty vials will be destroyed at the investigational site.

Unusable study vaccine, i.e. expired vaccine and vaccine having experienced a cold chain break **must not** be administered.

Following verification of vaccine accountability, all unused and unusable study vaccine will be destroyed at the clinical trial site.

XI. Collection of study variables

XI.1. Recording of data

A sheet with all results of physical examinations, medical history, vital signs and blood chemistry will be completed on every visit = CRF.

XI.2. Screening and demographic procedures

At the screening visit, the subject will be informed of the study objectives, obligations, benefits and risks and will be given time to ask questions. The subject must sign an informed consent document before any study related activities take place. The subject will be allocated a screening number. The subjects' eligibility for the study will be examined with regard to the inclusion/exclusion criteria and will include medical history, a full physical examination, assessment of vital signs and ECG, clinical laboratory evaluation, screening for hepatitis B and C and HIV, drug screening, measurement of pertussis toxin IgG antibody levels. Collection of demographic information will also be done at the screening visit.

XI.3. Safety

- Recording of adverse events. All adverse events, including findings at physical examination and vital signs, will be collected, documented and reported by the principal investigator/delegates as described in *XIII.2 Recording of safety parameters.*
- Reporting of adverse events. The principal investigator/subinvestigator will report all adverse events to the independent data monitoring committee and the sponsor.

XI.4. Laboratory safety assessment

All laboratory abnormalities which occur during the study will be evaluated by the principal investigator/sub-investigator and those deemed clinically significant will be reported to the independent data monitoring committee and the sponsor.

XII. Collection, transport and storage of biological samples

XII.1. RESPONSIBLE LABORATORIES

	Responsible laboratory	Time for delivery of samples
**Safety and other Labs:**		
Hematology, haemoglobin, WBC with differential (automatic) cell count, (i.e. granulocytes: neutrophils, eosinophils, and basophils, non-granulocytes: lymphocytes and monocytes) platelets (thrombocytes) and red blood cells (RBC).	Labmedicine Karolinska University hospital	
Blood chemistry	Labmedicine Karolinska University hospital	
Screening HIV, hepatitis B and C	Labmedicine Karolinska University hospital	
Total IgE	Labmedicine Karolinska University hospital	
Urine	Labmedicine Karolinska University hospital	
Urine drug analysis dipstick	Karolinska Trial Alliance	
Serum pertussis toxin IgG antibodies	SMI	<6 h RT
Nasopharyngeal culture for B. pertussis	SMI	<4 h RT
**Immunogenicity Labs and Sample Archive:**		
Binding pertussis IgG and IgA serum antibodies	SMI*	<6 h RT
Binding pertussis IgA saliva antibodies	SMI*	<6 h RT
Binding pertussis IgA nasopharyngeal antibodies	SMI*	<6 h RT
B cell ELISpot	SMI	<4 h RT
IFN-γ, IL-2 ELISpot	SMI	<4 h RT
Multicolour memory lymphoproliferative response	SMI	<4 h RT
Cryo-preserved cells, optional assays	SMI	<4 h RT

* a vial of the sample will be shipped in batch to Prof. Françoise Mascart, ULB, Belgium from SMI for analysis of antibodies against whole B. pertussis lysate
RT = room temperature

XII.2. LABELLING OF SAMPLES

The Laboratory Analytical Plan will contain detailed information about the labelling of the samples. All samples will have information about the study (Child Innovac), sponsor name (INSERM), the unique screening number (USN), initial of the subject and date of collection.

XII.3. BLOOD SAMPLES

Blood Collection

After the subject's eligibility has been verified and after the ICF has been signed by the subject and by the investigator all subjects will have blood samples collected according to the following schedule for

- Cell blood counts i.e Hematology (haemoglobin, total and differential (automatic) WBC (i.e. granulocytes: neutrophils, eosinophils, and basophils, non-granulocytes: lymphocytes and monocytes) platelets (thrombocytes) and red blood cells (RBC).
- Blood chemistry
- HIV and hepatitis screening
- Total IgE

- Pertussis specific humoral and cellular immunity

**Blood samples**								
**Study Visit**	**1**	**2**	**3**	**4**	**5**	**6**	**7**	**8**
	2-6 weeks prior to visit 2	Day 0	Day 4±1	Day 7±1	Day 11±1	Day 14±1	Day 28±1	5-6 months
**Safety Labs:**								
Hematology[e]	4 ml	4 ml		4 ml		4 ml	4 ml	4 ml
Blood chemistry[f]	11 ml							
Screening for HIV, hepatitis B and C (no anticoagulant)	3.5 ml							
Serum pertussis toxin IgG antibodies (no anticoagulant)	4.5 ml							
Total IgE (no anticoagulant)	3.5 ml						3.5 ml	3.5 ml
**Immunogenicity Labs and Sample Archive:**								
Binding pertussis IgG and IgA serum antibodies (no anticoagulant)		9 ml		4.5 ml		9 ml	9 ml	9 ml
B cell ELISpot (Na-heparin)		x		x[j]		x[j]	x	x
IFN-γ, IL-2 ELISpot (Na-heparin)		x				x[i]	x	x
Multicolour memory lymphoproliferative response (Na-heparin)		x					x	x
Cryo-preserved cells (Na-heparin)	x	x		x[j]		x[j]	x	x
Volume for cellular immunogenicity (Na-heparin)	4x8 ml	12x8 ml		5x8 ml		11x8 ml	12x8 ml	13x8 ml
TOTAL BLOOD VOLUME (ML)	58.5 ml	109 ml		48.5 ml		101 ml	112.5 ml	120.5

(e) Hematology (haemoglobin, total and differential (automatic) WBC (i.e. granulocytes: neutrophils, eosinophils, and basophils, non-granulocytes: lymphocytes and monocytes) platelets (thrombocytes) and red blood cells (RBC).
(f) Blood chemistry (potassium, calcium, sodium, creatinin, albumin, serum bilirubin, alkaline phosphatases, alaninaminotransferase (ALAT), aspartataminotransferase (ASAT), glutamyltransferase (GT), C-reactive protein, blood glucose, thyreoidea stimulating hormone (TSH).
(j) Samples from the first two subjects of each group will not be collected on day 7 (±1) and 14 (±1).
The blood sample should be drawn using aseptic techniques (see detailed procedure in the investigator's file). Blood must be collected in tubes with or without anticoagulant as indicated.

Blood samples transport

The blood samples for safety analysis should be transported to the department of Lab Medicine at the Karolinska University hospital, Solna.

The blood samples for immunology analysis should be transferred to the laboratory at SMI within 4 hours. At SMI the samples are handled according to the Laboratory analytical plan.

Samples for analysis of antibodies against whole B. pertussis lysate will be shipped from SMI to Prof. Françoise Mascart, ULB in Brussels after completion of the sample collection in accordance with the Laboratory analytical plan.

Serum samples management and storage

The samples without anticoagulant should be left at room temperature for 1-2 hours for clotting before centrifugation. After aliquoting, serum samples should be kept frozen at -18ºC or lower in two different freezers. The temperature of the freezers will be monitored weekly and recorded during the entire storage period.

Peripheral blood mononuclear cells (PBMC) management and storage

The samples with sodium heparin as anticoagulant should be handled at SMI within 4 hours after collection. After purification of PBMC according to the Laboratory analytical plan the cells are either used fresh for analysis of humoral and/or cellular immunity or will be aliquoted and stored frozen in two different liquid nitrogen containers.

XII.4. SALIVA SAMPLES

Saliva collection

After the subject's eligibility has been verified and after the ICF has been signed by the subject and by the investigator all subjects will have saliva samples collected according to the following schedule for analysis of pertussis specific IgA antibodies.

Saliva samples

**Study Visit**	**1**	**2**	**3**	**4**	**5**	**6**	**7**	**8**
	2-6 weeks prior to visit 2	Day 0	Day 4±1	Day 7±1	Day 11±1	Day 14±1	Day 28±1	5-6 months
**Immunogenicity Labs and Sample Archive:**								
Binding pertussis IgA saliva antibodies (OmniSal)		2x1 ml		2x1 ml		2x1 ml	2x1 ml	2x1 ml

The saliva samples should be collected by the Omni-Sal, which employs a compressed, absorbent cotton pad attached to a plastic stem. The pad should be placed under the tongue to absorb fluid from the bottom of the mouth. The device incorporates an indicator on the plastic stem that turns from white to blue when an adequate amount of sample has been collected. The collection pad should then be inserted into a stoppered transport tube containing 1.1 ml phosphate-buffered saline, pH 7.0, protease inhibitors, surfactants, antimicrobial agents and 0.2% sodium azide as a preservative.

The subject should not eat or drink 15 minutes prior to saliva collection. Further details will be described in the Laboratory analytical plan.

Saliva samples transport, management and storage

Detailed information will be available in the Laboratory analytical plan.

On the day of collection the samples should be transferred to the laboratory at SMI.

At SMI the collection pad will be compressed and the eluate will be filtered with a piston-style filter. The eluate should be aliquoted into at least 2 vials. The saliva should be kept frozen at -18ºC or lower in two different freezers temperature monitored weekly during the study analysis period. For long term storage (after the study analysis are completed) the saliva aliquots will be kept at -50ºC or lower in freezers with alarm.

Samples for analysis of antibodies against whole B. pertussis lysate will be shipped from SMI to Prof. Françoise Mascart, ULB in Brussels after completion of the sample collection in accordance with the Laboratory analytical plan.

XII.5. NASOPHARYNGEAL SAMPLES

Nasopharyngeal aspirate collection

After the subject's eligibility has been verified and after the ICF has been signed by the subject and by the investigator all subjects will have nasopharyngeal aspirates collected according to the following schedule for determination of

- Shedding of B. pertussis strain BPZE1 in the nasopharyngeal mucosa
- Analysis of pertussis specific IgA antibodies

Nasopharynx samples

**Study Visit**	**1**	**2**	**3**	**4**	**5**	**6**	**7**	**7'**	**8**
	2-6 weeks prior to visit 2	Day 0	Day 4±1	Day 7±1	Day 11±1	Day 14±1	Day 28±1	Day 45±5	5-6 months
**Safety and Other Labs:**									
Nasopharyngeal culture for B. pertussis			x	x	x	x	x[j]	x[j]	
**Immunogenicity Labs and Sample Archive:**									
Binding pertussis IgA nasopharyngeal antibodies		x		x		x	x		x

[j] If positive culture after 28 days (+-1) a new nasopharyngeal sample should be collected 2-3 weeks later for culture.

Using a vacuum pump the nasopharyngeal aspirate will be collected from the posterior pharynx along the floor of the nasopharynx. After aspirate collection the bardic feeding tube used will be flushed with 1 ml PBS. Further details will be described in the Laboratory analytical plan.

Nasopharyngeal samples transport, management and storage

Detailed information will be available in the Laboratory analytical plan.

The tip of the feeding tube will immediately (within 1 hour) be transferred to enrichment medium at the clinical trial site and thereafter transported to the laboratory where the enriched bacteria will be seeded on Bordet-Gengou blood agar plates.

The remaining aspirate will be transferred to the laboratory at SMI. The aspirate is aliquoted into at least 2 vials. For long term storage (after the study analysis are completed) the nasopharyngeal aspirate aliquots will be kept at -50ºC or lower in freezers with alarm.

Samples for analysis of antibodies against whole B. pertussis lysate will be shipped from SMI to Prof. Françoise Mascart, ULB in Brussels after completion of the sample collection in accordance with the Laboratory analytical plan.

XII.6. URINE SAMPLES

Urine collection

After the subject's eligibility has been verified and after the ICF has been signed by the subject and by the investigator all subjects will have urine collected for safety analysis of pH, erythrocytes, leucocytes, protein, glucose, ketones and bacteria (nitrite) and the drugs cocaine, amphetamine, cannabis, morphine, benzodiazepines, and methylenedioxymethamphetamine.

Urine samples and transport

Detailed information will be available in the Laboratory analytical plan.

The urine samples for safety analysis should be transported to the department of Lab Medicine at the Karolinska University Hospital, Solna.

Drug analysis will be performed with dipstick test at Karolinska Trial Alliance.

The samples will not be stored after completed analysis.

XII.7. WITHDRAWAL OF INFORMED CONSENT FOR DONATED BIOLOGICAL SAMPLES

All volunteers have the right to withdraw their consent for storage of biological samples at any time of the study without giving any reason. As a consequence the sample would be destroyed if asked by the subject.

XII.8. BIOBANK STORAGE OF BIOLOGICAL SAMPLES

After completion of the study all biological samples (i.e. from blood, saliva and nasopharynx samples) will be stored in the SMI biobank according to Swedish law with possibility for future analysis. If such analysis will be planned outside the scope of this study or the next study the project should be approved by the Local Ethics Committee who is the one to decide if the volunteers should give their consent. The samples are coded and identification of the donor of the samples will be available in a locked safe. Responsible for the samples at SMI is the Director General.

XIII. Recording and reporting of Adverse Events

XIII.1. DEFINITIONS

Adverse Event (AE)

An adverse event is any untoward medical occurrence in a patient or clinical trial subject administered an investigational medicinal product (IMP) and which does not necessarily have to have a causal relationship with this treatment.

An adverse event can therefore be any unfavourable and unintended sign (including an abnormal laboratory finding), symptom, or disease temporal associated with the use of a medicinal (investigational) product, whether or not considered related to the investigational medicinal product.

A pre-existing condition is a medical condition (including a condition being treated), which is diagnosed prior to the administration of the study vaccine or implementation of a protocol-specified intervention and which is documented as part of the subject's medical history.

Any **worsening** of a pre-existing condition temporal associated with the use of the study vaccine is by definition an adverse event. The start date of the adverse event is the date the pre-existing condition worsened.

Any **discovery** during the course of the study of a pre-existing condition not diagnosed prior to the administration of the study vaccine or protocol-specified procedure is by definition an adverse event. The start date of the adverse event is the date of discovery.

A surgical procedure is not an adverse event. The medical condition leading to the surgical procedure is the adverse event if it starts during the study period.

A protocol-specified intervention is a procedure specified in the study protocol (e.g.: blood sample). The procedure itself is not an adverse event; however, any condition resulting from the protocol-specified intervention is an adverse event, if it starts during the study period.

Serious Adverse Event (SAE)

Any untoward medical occurrence or effect that at any dose

- **Results in death**
- **Is life-threatening**

Life-threatening in this context refers to an event in which the patient was at risk of death at the time of the event; it does not refer to an event, which hypothetically might have caused death if it were more severe.

- **Requires in-patient hospitalisation or prolongation of existing hospitalisation**

An in-patient hospitalisation implies admission to a department (a patient is allocated a bed, regardless of length of stay), and not a hospital consultation (even an emergency consultation). A hospitalisation for treatment, monitoring, further investigations, without a bed allocation is an out-patient hospitalisation and therefore does not constitute a serious adverse event. A hospitalisation for an elective procedure (e.g. medical conditions leading to surgery that started prior to the study but did not worsen during the study) or for a pre-existing condition, which has not worsened does not constitute a serious adverse event

- **Results in a persistent or significant disability/incapacity**

Means substantial disruption of one's ability to conduct normal life function

- **Is a congenital anomaly/birth defect**
- **Is another medically important event**

Medical and scientific judgement should be exercised in deciding whether other situations should be considered serious events, such as "important medical events" that might not be immediately life threatening or result in death or hospitalisation but might jeopardise the patient or might require intervention to prevent one of the other outcomes listed above.

Adverse Reaction (AR)

Adverse reactions are all untoward and unintended responses to an investigational medicinal product related to any dose administered. All adverse events judged by either the reporting investigator or the sponsor as having a reasonable causal relationship to a medicinal product qualify as adverse reactions. The expression reasonable causal relationship means to convey in general that there is evidence or argument to suggest a causal relationship.

An adverse event is considered an adverse reaction if the investigator or sponsor (applicable only for serious adverse event) indicates that the adverse event is possibly, probably or definitely related to the study vaccine or if the relation with the study vaccine has not been indicated.

In addition, any serious adverse event assessed by the investigator as possibly, probably or definitely related to a protocol-specified intervention is considered a serious adverse reaction.

Relationship of an Adverse Event to the Study Vaccine

The relationship of an adverse event to the study vaccine has to be determined by the PI or delegate as follows:

- **NONE** [no relation with the study vaccine]: subject has not received the study vaccine or inconsistent temporal relationship (excessively long interval between administration of IMP and the onset of the symptom or the symptom appeared before administration of IMP) or there is *another obvious or more likely cause of the adverse event.*
- **POSSIBLE** [possible relation with the study vaccine]: has a temporal relationship with the study vaccine; however, a potential alternative aetiology that may be responsible for the symptom has not been investigated. *The adverse event could have been due to another equally likely cause.*
- **PROBABLE** [probable relation with the study vaccine]: has a relevant temporal relationship with the study vaccine, suggestive symptoms and a potential alternative aetiology is not apparent. *The adverse event is more likely explained by vaccine than by another cause.*
- **DEFINITE** [definite relation with the study vaccine]: has a relevant temporal relationship with the study vaccine and no alternative aetiology is apparent (after investigation other aetiologies have been ruled out, *the adverse event is most likely explained by vaccine than by another cause)*, or positive rechallenge with suggestive symptoms or reaction at the site of injection.

Intensity of an AE / AR

The adverse events will be graded according to the Food and Drug Administration guidelines Appendix 4 *Toxicity Grading Scale for Healthy Adult and Adolescent Volunteers Enrolled in Preventive Vaccine Trials.*
For systemic adverse events, the intensity recorded in the CRF will be the maximum intensity observed.

- mild: awareness of sign or symptom but easily tolerated
- moderate: discomfort enough to cause interference with usual activity
- severe: incapacitating with inability to do usual activity

For fever, the intensity recorded in the CRF will be the highest observed temperature of the day measured oral in degrees Celsius if temperature is taken more than once a day otherwise the evening temperature.

XIII.2. Recording of Safety Parameters

Immediate AE

To evaluate immediate adverse events, subjects will be kept at the investigator site under surveillance of the investigational site staff for at least 6 hours following vaccination.

Adverse events (respiratory tract adverse reactions and systemic adverse events, serious or not) occurring during this time period will have to be reported by the investigator or delegate in accordance with applicable laws (LVFS 2003:6, Patientdatalagen 2008:355)

Local AE from the respiratory tract

Local AE which lead to difficulties in breathing will be considered as serious and reported to the independent data monitoring committee and sponsor.

All other local AE from the respiratory tract will be recorded in the volunteer´s CRF.

After the immediate medical surveillance at the investigator site, local respiratory tract adverse events occurring from the day of vaccination (Day 0) to Day 14 following vaccination will be recorded in the Diary by the subject:

- Solicited adverse reactions from the respiratory tract that are pre-listed in the Diary or Internet Form will be daily documented from Day 0 to Day 14
- Unsolicited adverse reactions from the respiratory tract will be spontaneously recorded in the Diary from Day 0 to Day 28.

At Visit 7, 28 days (-1: +7) after vaccination, the Diary will have to be returned to the investigational site by the subject. The investigator or delegate will check the Diary, interview the subject and record adverse reactions from the respiratory tract in the CRF.

Systemic AE

After the immediate medical surveillance at the investigator site, systemic adverse events occurring from the day of vaccination (Day 0) to Day 28 following vaccination will be recorded in the Diary or Internet Form by the subject:

- Solicited systemic adverse events (pyrexia, headache and pain) that are pre-listed in the Diary or Internet Form will be daily documented from Day 0 to Day 14
- Unsolicited systemic adverse events will be spontaneously recorded in the Diary or Internet Form from Day 0 to Day 28

At Visit 7 the Diary will have to be returned to the investigational site by the subject. The investigator or delegate should check the Diary or Internet Form, interview the subject and record systemic adverse events in the CRF.

Temperature

After the immediate medical surveillance at the investigator site, numeric values of temperature taken from the day of vaccination (Day 0) to Day 14 following vaccination will be recorded in the Diary or Internet Form by the subject.

- Daily from Day 0 to Day 14
- Each time the subject is feverish from Day 15 to Day 28

Tympanic measurement of temperature is not allowed. If temperature is measured more than once during a day, the highest temperature measured that day should be recorded in the Diary or Internet Form.

At Visit 7 the Diary or will have to be returned to the investigational site by the subject. The investigator or delegate should check the Diary or Internet Form, interview the subject and record

numeric values of temperature in the CRF.

Serious Adverse Event (SAE)

All serious adverse events should be reported from Visit 1 (screening visit) to Visit 8.

After the immediate medical surveillance at the investigator site, any hospitalisations and/or visits to a physician (within the definitions of SAE) from the day of vaccination at Visit 2 (Day 0) to Visit 7 will also be recorded on the Diary by the subject.

The subject should be instructed to immediately contact the investigator in case of any serious adverse event.

At Visit 7, the Diary or Internet Form will have to be returned to the investigator site by the subject. The investigator or delegate should check the Diary or Internet Form, interview the subject and record the serious adverse events in the CRF.

Any SAE should be recorded by the investigator in a "*Serious adverse event form*" to be sent to Independent data monitoring Committee and to INSERM within 24 hours of the investigator becoming aware of the event (see section XIII.3 "*Reporting of Adverse Events*").

General requirements

All efforts should be made by the investigator to retrieve follow-up information for all serious adverse events.

In case of premature discontinuation of a subject, particular efforts should be made to collect any adverse event, serious and not, that occurred at least within 14 days following the study vaccine administration.

The investigator should follow until their complete disappearance (resolution) or stabilisation:

- All serious adverse events, which persist at the time of the last visit of the concerned subject (Visit 8). The investigator will inform the Independent data monitoring Committee and INSERM of the date of resolution or stabilisation of the adverse event and will document it.
- Non-serious adverse events assessed as possibly, probably or definitely related to the study vaccine by the investigator which persist 28 days after vaccination of the concerned subject (Visit 7)

Any serious adverse event likely to be related to the study vaccine or to the protocol specified-intervention and occurring after subject's study termination will not be recorded into the CRF but should be reported by the investigator to the sponsor INSERM (see section XIII.3 *"Reporting of Adverse Events").*

Regarding subjects screened but not included (screening failure), the SAE occurring during the 1st visit will be reported.

XIII.3. Reporting of Adverse Events by the investigator

Contact persons at INSERM for the reporting of adverse events

Béatrice Barraud
INSERM
101, rue de Tolbiac, F-75654 Paris, Cedex 13, France
Phone:+ 33 1 44 23 67 29
Fax: + 33 1 44 23 67 10

Email: rqrc.siege@inserm.fr

The anonymity of subjects shall be respected when the investigator reports adverse events to the sponsor INSERM

Reporting of SAE

Serious adverse events must be reported by the investigator to INSERM **as soon as possible but not later than 1 business day** of knowledge by the investigator. This notification must be made regardless of the relationship to vaccination.

The initial notification can be made by faxing the completed and signed "*Serious adverse event form* ***(initial)***" to:

Responsable de la Mission Réglementation et Qualité et recherche clinique (RQRC)

Fax : +33 1 44 23 67 10

Tél : +33 6 85 36 09 50

E-mail : rqrc.siege@inserm.fr

Any relevant information concerning the serious adverse event that becomes available after the "*Serious adverse event form* ***(initial)***" has been sent (outcome, precise description of medical history, results of the investigation, copy of hospitalisation report, etc.) should be forwarded as soon as possible of knowledge by the investigator, by completing a "*Serious adverse event form* ***(follow-up)***".

This form should be signed by the investigator and sent by fax to the person listed above.

Reporting of other safety information

At any time throughout the study period the investigator could contact INSERM to report any suspected vaccine-related non-serious adverse event occurring outside the period of collection defined in the CRF or any information of safety interest (e.g. overdose, vaccine failure, misuse or medication error) - refer to section XIII.3. "*Reporting of serious adverse events*" for Pharmacovigilance contact details.

XIII.4. ASSESSMENT AND REPORTING OF ADVERSE EVENTS

In order to comply with European and local regulations on serious adverse event reporting to Health Authorities and Ethics Committees and to allow INSERM to carry out a precise analysis of the safety of its vaccine, the investigator is committed to documenting accurately the event, to respecting notification deadlines described in section XIII.3 "*Reporting of Adverse Events*", to providing INSERM with all necessary information and, if requested by INSERM, to giving access to de-identified source documents.

According to available information and current medical knowledge, INSERM will assess the causal relationship with the study vaccine. A serious adverse event will be considered a vaccine-related serious adverse event (i.e. serious adverse reaction) if either the investigator or INSERM indicates that the serious adverse event is possibly, probably or definitely related to the study vaccine. The causality assessment given by the investigator will not be downgraded by INSERM. The opinion of both the investigator and INSERM will be provided with the report.

INSERM will also determine the expectedness of the SAR based on the Investigator Brochure of the study vaccine.

XIII.5. REPORTING OF SUSAR

Any unexpected serious adverse event assessed as possible, probable or definite to the study vaccine (SUSAR) will be expedited reported in a timely manner.

Notification of SUSAR, death or life-threatening reactions to Swedish Medical Product Agency, EMEA Eudravigilance database, Ethics Committees and investigators will be performed by the sponsor.

The sponsor will also be responsible for the prompt notification to all concerned investigators, Ethics Committees and Competent Health Authorities of findings that could adversely affect the safety of subjects.

A contemporaneous copy of all reports sent to Swedish Medical Product Agency, EMEA Eudravigilance database, Ethics Committees and investigators will be sent by fax to SMI:

Swedish Institute for Infectious Disease Control (SMI)
Fax: +46 8 33 74 60
Att: Rigmor Thorstensson

Regarding the notification to the Ethics Committees, SMI will act on behalf of the sponsor.

XIII.6. ANNUAL SAFETY REPORT

The sponsor shall submit a report to the Medical Products Agency and the Ethics Committee at least three months after the end of the study, of all suspected, unexpected, serious adverse reactions that have occurred during the period, including an assessment of the safety of the persons participating in the trial as trial subjects.

XIII.7. INDEPENDENT DATA MONITORING COMMITTEE (IDMC)

The committee will consist of

Prof. Ragnar Norrby

Address: Primusgatan 104, SE-11267 STOCKHOLM, Sweden

Phone: +46-8-679 91 04

E mail: ragnar.norrby@gmail.com

Prof. Patrick Olin

Address: Valhallavägen 157, SE-115 53 STOCKHOLM, Sweden

Phone: +46-8-6608891

Mobile: +46-708-703511

E mail: patrick.olin@mailbox.euromail.se

Prof. Roland Dobbelaer

Address: Waasmunsterbaan, 26, B-9160 LOKEREN, Belgium

Phone: +32 9 348 33 37

E mail: roland.dobbelaer@skynet.be

Dr. Odile Launay
Address : Centre d’investigation clinique de vaccinologie Cochin Pasteur, Groupe Hospitalier Cochin Saint-Vincent de Paul, 27, rue du Faubourg Saint Jacques, 75 679 PARIS cedex 14, France
Phone: + 33 1.58.41.28.60
Email: odile.launay@cch.aphp.fr

XIV. Evaluation criteria

XIV.1. Safety (primary)

The safety evaluation criteria will be the number of subjects with the following adverse events:

From Day 0 (Visit 2) to Day 7

Solicited and unsolicited adverse reactions from the respiratory tract:

Local AE which lead to difficulties in breathing will be considered as serious and reported to the independent data monitoring committee and sponsor.

All other local AE from the respiratory tract will be recorded in the volunteer´s CRF.

From Day 0 to Day 28:

Solicited systemic adverse events (pyrexia, headache, pain)

Unsolicited respiratory tract reactions

Unsolicited systemic adverse events

Serious adverse events (SAE) should be reported from the signing of the ICF and throughout the study period.

XIV.2. Attachment of the BPZE1 strain to the nasopharyngeal mucosa (secondary)

The ability of the modified B. pertussis strain to colonise the human respiratory tract and for how long the microorganism is shedding. BPZE1 bacteria will be detected in nasopharyngeal samples collected twice a week during the first 2 weeks and after 4 weeks by cultivation on Bordet-Gengou blood agar plates. If the BPZE1 is still detectable in a sample collected 4 weeks after vaccination a new sample must be collected 2-3 weeks later to confirm the clearance of the vaccine strain BPZE1. The number of days during which bacteria are detected will be determined

XIV.3. Immunogenicity (secondary)

Pertussis antibodies will be measured in serum before and 7, 14, 28 days and 5-6 months after vaccination. The number with positive IgA and IgG antibody response will be measured to the following antigens:

- Pertussis toxin
- Filamentous hemagglutinin A
- Fimbriae 2/3
- Pertactin
- Whole Bordetella pertussis cell lysate

A positive antibody response after the vaccination is defined as follows: At least 100% increase from pre- to postvaccination, to at least 4 times MLD (minimum level of detection) for pertussis toxin, filamentous hemagglutinin A, pertactin, fimbriae 2/3 and whole Bordetella pertussis cell lysate in the postvaccination sample

To describe in each vaccine group pre- and postvaccination

- Number of responders and levels of IgG and IgA antibodies to pertussis toxin, filamentous hemagglutinin, pertactin, fimbriae and whole Bordetella pertussis cell lysate in serum.
- Number of responders and levels of IgA antibodies to pertussis toxin, filamentous

hemagglutinin, pertactin, fimbriae and whole Bordetella pertussis cell lysate in saliva and nasopharyngeal aspirate (exploratory assays)

- Number of responders and levels of T cell immune responses by measuring production of cytokines and T cell memory after stimulation with pertussis toxin, filamentous hemagglutinin, and pertactin (exploratory assays)
- Number of responders and levels of B cell immune responses as determined by the numbers of effector and memory B cells after stimulation with pertussis toxin, filamentous hemagglutinin

For all assays the geometric means should be displayed for pre- and postvaccination samples in each vaccine group.

Method for measurement

Sera will be tested blindly for pertussis specific IgA and IgG antibodies by validated enzyme-linked immunoassay methods. Saliva and nasopharyngeal samples will be tested blindly for pertussis specific IgA by the same validated enzyme-linked immunoassays. Antibody levels to PT, FHA, fimbriae 2/3, pertactin and whole Bordetella pertussis cell lysate will be expressed in ELISA units (EU/mL) calibrated against reference antisera from the National Institute for Biological Standards and Controls.

For the pre- and post-vaccination samples the number of memory and effector B cells specific for PT, FHA, and pertactin will be determined by a B cell ELISpot assay and the number of cells producing IFN-γ and IL-2 after stimulation of the peripheral blood mononuclear cells will be measured by T cell ELISpot and flow cytometric analysis.

XV. Statistical considerations

Interim safety meetings will be held before administering the next higher dose.

The study will be too small to make statistical evaluations of serious adverse events. In this case evaluation of single cases will be necessary by the prinicipal clinical investigator, the Independent data monitoring committee and the sponsor.

Descriptive statistics will be provided for demographic and other baseline characteristics.

XV.1. LOCAL SYMPTOMS

Any local symptoms from the respiratory tract will be compared for each of the 3 vaccine groups with the control group and between the 3 vaccine groups. Fisher´s exact test will be used for comparison of proportions.

XV.2. HEMATOLOGY

Pre- and post-vaccination values of cell blood counts (haemoglobin, total and differential WBC and platelets) will be compared for each group separately with a-test for paired samples. Depending on the distribution of values, no transformation, geometric transformation of rangtransformation will be performed. Thereafter mean, geometric mean and/or median values between all vaccine groups and the control group and between all vaccine groups will be compared separately using an appropriate t-test for geometric mean values and Mann-Whitney U test for median values.

XV.3. IMMUNOGENICITY ANALYSIS

All immunologic tests (serum IgG and IgA antibodies, saliva IgA and nasopharyngeal aspirate IgA; B- and T-cell memory cells) will be compared in the same way as the hematology test, but it will not be decided whether geometric mean values or median values will be used until the distribution of the results are known. Most likely serum antibody levels (the most important immunologic variable) will be logarithmically transformed and geometric mean values will be compared. This procedure is almost always the case for serum antibody levels.

XVI. Administrative and regulatory requirements

XVI.1. Study documents and retention of records

Study documents include Case Report Forms (CRF), "*Serious Adverse Event"* Forms, data correction/ request forms, source documents, study books and appointment schedules, investigator correspondence and regulatory documents (*e.g. confidentiality agreement, signed protocol and amendments, Ethics Committee correspondence and approval, approved and signed consent forms, receipts for clinical supplies and records for their dispensing*).

Source documents include all recorded observations or notes of clinical activities and all reports and records necessary for the evaluation of the clinical study. They include, but are not limited to, laboratory reports, progress notes, and any other reports or records of procedures performed in accordance with the protocol. The Diary is a source document.

Whenever possible, the original recording of an observation should be retained as a source document. However, a photocopy is acceptable if certified by the investigator, provided it is clear, legible and an exact duplication of the original document.

The investigators and/or institutions will have to permit study-related monitoring, audits, Independent Review Board and/or Independent Ethic Committee review and regulatory inspections, providing direct access to source data and source documents.

The investigator will have to retain the study documents for 15 years after completion or discontinuation of the trial unless specified otherwise by the sponsor for a longer retention period.

XVI.2. Recording of data

The subject medical journal and the Diary are source documents.

However, study-related data obtained during the study visit and which, from a medical perspective, are not essential for subject follow up in routine medical practice, may be recorded directly in the CRF (source data). All study data location will be described in the "source data location form". All data in the protocol will be documented in the CRF. No other data will be collected.

XVI.3. Data quality assessment

CRF will be provided by SMI to record all subject data.

Periodically, a CRA working on behalf of INSERM will review study documents to verify compliance with the protocol, GCP and the accuracy of the data referring to the source documents.

Periodicity of the monitoring visits will be defined in the monitoring plan.

All information requested in the CRF will have to be completed. Whenever information is not available, this should be noted. All entries must be made with a ballpoint pen.

Any correction or changes made on the CRF must be initialled and dated by the investigator or delegate. In such cases, the incorrect entry must be crossed out with a single line in order to remain legible. The use of correction fluid is not allowed.

The last page of each CRF will have to be signed by the investigator before it is sent to SMI to certify that all the data recorded in the CRF are consistent with the source data and reflect the status of the subject during the corresponding part of the study. For any unsigned CRF, the investigator will be asked (correction/clarification forms) to confirm CRF data.

After monitoring, the original of the CRF page will be sent to SMI for data management and a copy will be kept and archived by the investigator at site. The information collected in the CRFs will be reviewed for legibility and captured in a database. To avoid possible inconsistencies, logical controls and tests

of validation will be carried out on this database which will be subject to both human visual and computer driven procedures in order to maximize logical consistencies in the collected CRF data.

Inconsistencies will generate queries, which will be sent to the investigator. The investigator or delegate will have to answer queries and signed them to authorize any database modification.

INSERM requires the investigator or subinvestigator to obtain documented consent from each potential subject for the clinical study.

Written informed consent must be obtained from the subject before participation in the study and before any study related procedure commences (e.g. collection of blood sample). The signature on the ICF must be dated by the subject. Consent form must be dated and signed by the principal investigator or subinvestigator.

One signed consent must be kept by the investigator or delegate and a copy of the signed consent must be given to the subject, a third copy will be collected in sealed envelopes and kept by SMI (investigator). Moreover, a duplicate of each consent will be kept and sent to the sponsor at the end of the study in a sealed and opaque envelop.

XVI.4. DATA MANAGEMENT

The study will be reported to the person responsible for handling with personal identifiers under the Swedish law ("personuppgiftslagen") at SMI.

Data entry

The study will be conducted by the Karolinska Trial Alliance, where investigators and study nurses will enter data on vaccinations and adverse events in the CRF.

Data management

The Data management procedure will be performed in accordance with ICH guidelines and Standard Operating Procedures and conducted in accordance with good clinical, scientific and data management principles.

All the data in the CRFs and diaries will be reviewed for legibility and entered into a SAS database which will be subject to both human visual and computer driven procedures in order to maximize logical consistencies in the collected CRF data. All data will be single entered with 5% verification.

Approved data of the immune response determinations will be entered on a computer at SMI. All data items will be verified by check-reading

Data analysis

Data analysis will be undertaken by the investigators and a statistician at SMI.

XVI.5. ETHICS COMMITTEE

Karolinska Trial Alliance, Karolinska University Hospital will submit an application to the relevant Swedish Ethics Committee for approval of the clinical study. A copy of the Ethics Committee's approval must be received by INSERM and SMI before study starts.

Any substantial amendment that becomes necessary during the course of the study and approved by the sponsor must be submitted to the same Ethics Committee and needs to receive a positive approval before entering into force (*in case of refusal, the subjects will still have to be followed*).

Any minor change or modification (*e.g., modification of clinical supply procedure with no change in dose and/or route and/or schedule of administration*) will be notified to the Ethics Committee for information only.

INSERM (or delegate) will notify the end of the clinical study to the Ethics Committee within 90 days following the end of the clinical study.

XVI.6. Health Authorities

INSERM (or delegate) will submit an application to the relevant Swedish Health Authorities for approval of the clinical study. A copy of the Health Authorities approval must be received by INSERM (or delegate) before study starts.

Any substantial amendment that becomes necessary during the course of the study must be submitted to the same Health Authorities and needs to receive a positive approval before entering into force (*in case of refusal, the subjects will still have to be followed*).

Any minor change or modification (*e.g., modification of clinical supply procedure with no change in dose and/or route and/or schedule of administration*) will be notified to the Health Authorities for information only.

INSERM (or delegate) will notify the end of the clinical study to the Health Authorities within 90 days following the end of the clinical study.

XVI.7. Confidentiality

By signing this protocol, the principal investigator undertakes that the protocol and all attached information are and will be kept confidential. The Principal investigator agrees that after providing the protocol and all information attached to the Ethics Committee, affiliated institution and/or specified employees, he/she remains responsible for their overall confidentiality. Such obligation is detailed in the confidentiality agreement signed by the investigator prior to the initiation of the study.

The Principal investigator agrees that, subject to local regulations and ethical considerations, an INSERM representative or any regulatory agency may consult directly and/or copy study documents in order to verify a case report, provided that the subject's identity remains anonymous.

The investigator undertakes to treat all subjects' data used or disclosed in connection with the conduct of study in compliance with European and Swedish applicable laws relating to data protection.

XVI.8. Compliance with Law and Audit

The investigator and INSERM agree to conduct the study in an efficient and diligent manner in accordance with this protocol, ICH Good Clinical Practices standards, Declaration of Helsinki and applicable regulatory requirements [see GCP 6.2.5] (Appendix 3) as well as any European and Swedish applicable laws and regulations relating to the conduct of the study.

The principal investigator will prepare and maintain complete and accurate study documents for each subject participating in the study. The investigator will promptly submit to INSERM (or delegate) all original case report forms and other reports as required by this protocol following completion or termination of the clinical study or as otherwise required by INSERM.

Study documents and source documents will be promptly and fully disclosed to INSERM by the investigator upon request and will be made available at the investigational site upon request for inspection, copying, review and audit by INSERM representatives or any regulatory agency representatives, provided that the subject´s identity remains anonymous.

Persons prohibited from conducting or working on clinical studies by any court or regulatory agency will not be allowed to conduct or work on studies sponsored by INSERM. The investigator will immediately inform INSERM in writing if any person involved in conducting the study is prohibited from conducting or working on clinical studies by any court or regulatory agency or if any proceeding against this person is pending.

XVI.9. INSURANCE

The Swedish Patient Insurance Scheme, i.e. Patientskadelagen (SFS 1996:799) cover all subjects in the clinical trial in a similar way as during other medical care. INSERM as sponsor takes out an insurance (Responsabilité Civile) for the duration of study.

XVI.10. PUBLICATIONS AND DATA PROPERTY

Intellectual property rights and dissemination activities including but not restricted to publications and presentations shall be governed by the rules defined in the European consortium agreement Child – Innovac

XVII. Study management

XVII.1. TRAINING OF STUDY SITE PERSONNEL

SMI will provide SOPs describing the procedures for handling, and storage of the study vaccine, vaccination, sample collection, transport and storage, data handling.

The procedure for vaccination and collection of nasopharyngeal specimens will be demonstrated by staff from SMI.

XVII.2. MONITORING OF THE STUDY

A plan for monitoring the study will be provided by the sponsor before the study starts.

XVIII. List of appendices

- APPENDIX 1. ENVIRONMENT RISK ASSESSMENT FOR B. PERTUSSIS STRAIN BPZE1
- APPENDIX 2 COPY OF OFFICIAL FRENCH AGREEMENT ON THE DOWNGRADING OF BPZE1 AS A CLASS I ORGANISM
- APPENDIX 3. WORLD MEDICAL ASSOCIATION DECLARATION OF HELSINKI
- APPENDIX 4. FDA TOXICITY GRADING SCALE FOR HEALTHY ADULT AND ADOLESCENT VOLUNTEERS ENROLLED IN PREVENTIVE VACCINE CLINICAL TRIALS

APPENDIX 1

XVIII.1. ENVIRONMENT RISK ASSESSMENT FOR A VACCINE TO BE USED IN THE CHILD INNOVAC PHASE I CLINICAL TRIAL / MILJÖRISKANALYS FÖR VACCIN INGÅENDE I CHILD INNOVAC 1

I. **Allmänna uppgifter**

A. Sökandens (företag, institution eller motsvarande) namn och adress.
The Swedish Institute for Infectious Disease Control (Smittskyddsinstitutet), 171 82 Solna, Sweden
B. Den eller de ansvariga forskarnas namn, utbildning och erfarenhet.
Birger Trollfors, MD, professor, principal clinical investigator, with more than 20 years of experience with clinical vaccine trials, especially pertussis vaccine trials; trained in GCP
Rigmor Thorstensson, PhD, assoc. professor, principal laboratory investigator, with more than 20 years of experience with follow up of immune responses after infection and vaccination; trained in GCLP.
C. Projektets namn.
A phase 1, single centre, dose-escalating, placebo-controlled study of a genetically modified B. pertussis strain given as a single intranasal dose to healthy adult male volunteers

II. **Uppgifter om den genetiskt modifierade organismen**

A. Egenskaper hos a) givarorganism, b) mottagarorganism eller c) moderorganism (i förekommande fall)
1. Vetenskapligt namn.
Bordetella pertussis (*B. pertussis*)
2. Taxonomi.
Bordetella
3. Övriga namn (vedertaget namn, stamnamn osv.).
Bordetella pertussis (*B. pertussis*) strain Tohama Japan WS 21/08/1987 (Glaxo SmithKline)
4. Fenotypiska och genetiska markörer.
The *B. pertussis* strain is a Gram-negative coccobacillus: aerobic, encapsulated. *B. pertussis* has fastidious growth requirements.
5. Grad av släktskap mellan givar- och mottagarorganism eller mellan moderorganismer.
None
6. Beskrivning av identifierings- och detekteringsmetoder.
B pertussis is detected by culture on selective media (Bordet Gengou agar plates with 20% sheep blood) and identified by molecular techniques based on PCR amplification.
7. Detekterings- och identifieringsmetodernas känslighet, tillförlitlighet (i kvantitativa termer) och specificitet.
Cultivation on selective medium is highly specific for detection of *B pertussis*, but the sensitivity has been reported to be only 50% for diagnosis of pertussis infection. The collection procedure has a great impact on the recovery of *B pertussis*. The recovery of *B pertussis* can be improved by different means for collection such as use of aspirate instead of swab, bedside culture vs transport, enrichment on Regan Lowe substrate, collecting an additional second aspirate.
The PCR method is highly specific and sensitive with a lower detection limit of approximately 30 bacteria.
8. Beskrivning av organismens geografiska utbredning och naturliga livsmiljö, inklusive information om naturliga predatorer och bytesorganismer, parasiter och konkurrenter, symbionter och värdorganismer.
B pertussis is spread world wide and causes 30-50 million cases of whooping cough and approximately 200,000 – 400,000 deaths are reported annually despite extensive immunization programs. The bacteria colonize in the human respiratory tract and display a strong tropism for the cilia of the respiratory mucosa, which seem the only site of infection for these bacteria. Colonization is followed by proliferation on the ciliated mucosal surface, resulting in damage of the respiratory epithelium, induction of mucus release and an inflammatory influx in the lumen of the respiratory tract. Disruption of the ciliated mucosal function and damage to the respiratory epithelium are the primary pathologies associated with *Bordetella* infections. The disease is very contagious and typically manifested in children with paroxysmal spasms of severe coughing, whooping and posttussive vomiting. Major complications of whooping cough include hypoxia, pneumonia, seizures, encephalopathy and malnutrition. *B. pertussis* is predominately a human pathogen and there is no known animal vector or reservoir.

The parental strain Tahoma used to produce the GMO is widely used in laboratories.
9. Organismer med vilka det är känt att överföring av genetiskt material förekommer under naturliga förhållanden.
The potential for exchange of genetic material is virtually inexistent, since *B. pertussis* does not harbour plasmids or conjugative transposons. In addition *B pertussis* Tahoma strain (background used for the BPZE1 GMO) does not harbour intact prophage genomes and is therefore incapable of producing functional phage particles.
10. Verifiering av organismernas genetiska stabilitet och faktorer som påverkar denna.
N/A
11. Patologiska, ekologiska och fysiologiska egenskaper:
a) Faroklassificering enligt gällande gemenskapsregler för skyddet av människors hälsa och miljön.
B. pertussis colonization is strictly limited to respiratory epithelium without extrapulmonary dissemination of the bacteria. Although pertussis in immunocompromised individuals, such as HIV-infected subjects, have been described occasionally, it is rare in AIDS patients. There is no known animal vector or reservoir for *B. pertussis*, which is predominately a human pathogen although a number of animal models (mice, rats, rabbits and non-human primates) have been described.
B. pertussis is classified as a biosafety level 2 pathogen.
b) Generationslängd i naturliga ekosystem, sexuell och asexuell reproduktionscykel.
In optimal laboratory culture conditions the generation time is approximately 4 hours. The reproduction is asexual
c) Uppgifter om överlevnadsförmåga, inklusive anpassning till årstidsväxlingar samt förmåga att bilda överlevnadsstrukturer.
Limited knowledge. One report from Canadian authority reports *"B. pertussis* is susceptible to cold temperatures and desiccation. *B. pertussis* survives 1-2 hours on surfaces; 3-4 hours in human sputum samples and up to 7 days in diluted saliva; 19-20 hours in air and 3-5 days on plastic".
d) Patogenicitet; infektionsförmåga, toxicitet, virulens och allergicitet, bärare (vektor) av patogener, möjliga vektorer, spektrum av värdorganismer inklusive icke-målorganismer, möjlig aktivering av latenta virus (provirus). Förmåga att kolonisera andra organismer.
B. pertussis produces several virulence factors including toxins. Among them, pertussis toxin (PTX) has been shown to induce systemic toxic effects. Tracheal cytotoxin (TCT) is likely to be responsible for the cough syndrome. *B pertussis* is usually spread by coughing individuals. There is no known animal vector or reservoir for *B. pertussis*, which is predominately a human pathogen although a number of experimental models (mice, rats, rabbits and non-human primates) have been described.
e) Antibiotikaresistens och möjlig användning av dessa antibiotika för profylax och behandling av människor.
The standard therapy for *B. pertussis* infection is the treatment with the antibiotic erythromycin. Other antibiotics that may be used for therapeutic intervention are azithromycin, clarithromycin and trimethoprim/sulfamethoxazole
f) Medverkan i miljöprocesser; primärproduktion, näringsomsättning, nedbrytning av organiskt material, respiration osv.
N/A
12. Egenskaper hos naturliga vektorer:
Plasmid vector was used for transformation but no plasmid material is left in the final GMO strain
a) Sekvens.
N/A
b) Mobiliseringsfrekvens.
N/A
c) Specificitet.
N/A
d) Förekomst av gener som överför resistens.
N/A
13. Historik över tidigare genetiska modifieringar.
N/A
B. Vektorns egenskaper
Plasmid vector was used for transformation but no plasmid material is left in the final GMO strain
1. Vektorns beskaffenhet och ursprung.
N/A
2. Sekvens av transposoner, vektorer och andra icke-kodande genetiska segment, som används för att konstruera en viss genetiskt modifierad organism och för att uppnå att den införda vektorn och den infogade DNA-sekvensen fungerar i denna.
N/A
3. Den införda vektorns mobiliseringsfrekvens eller förmåga att överföra genetiskt material samt metoder för att fastställa detta.

N/A

4. Uppgifter om i vilken omfattning vektorn är begränsad till det DNA som krävs för den avsedda funktionen.

N/A

C. Den modifierade organismens egenskaper

1. Uppgifter om den genetiska modifieringen.

BPZE1 is an attenuated *Bordetella pertussis* (*B. pertussis*) strain which has the following genetic modifications: deletion of the dermonecrotic toxin (*dnt*), substitution of the *ampG* gene of *B. pertussis* for the *ampG* gene of *Escherichia coli* (*E. coli*) and 2 nucleotide mutations in the pertussis toxin (*ptx*) leading to amino acid replacements of Arg9 by Lys and Glu129 by Gly in the substrate binding and catalytic site respectively.

a) Använda modifieringsmetoder.

b) Metoder som använts för att konstruera och införa den eller de aktuella DNA-sekvenserna i mottagaren eller för att ta bort en sekvens.

c) Beskrivning av det införda genmaterialets eller vektorns uppbyggnad.

d) Det införda genmaterialets renhet från okända sekvenser samt uppgifter om i vilken omfattning den införda sekvensen är begränsad till det DNA som krävs för den avsedda funktionen.

e) Metoder och kriterier som används för selektion.

f) Sekvens, funktionell identitet och lokalisering av berörda ändrade, införda eller borttagna nukleinsyrasegment, med särskild hänvisning till eventuellt förekommande känd skadlig sekvens.

Citation from Mielcarek et al 2006_PLoS Pathogens, July 2006, Volume 2, Issue 7 Supplement

Construction of B. pertussis BPZE1

To construct B. pertussis BPZE1, we first replaced the *B. pertussis ampG* gene by *Escherichia coli ampG* using allelic exchange. A PCR fragment named met and located at position 49,149 to 49,990 of the B. pertussis genome (http://www.sanger.ac.uk/Projects/B_pertussis/), upstream of the *B. pertussis ampG* gene, was amplified using oligonucleotides A : 5’-TATAAATCGATATTCCTGCTGGTTTCGTTCTC-3’ and B : 5’-TATAGCTAGCAAGTTGGGAAACGACACCAC-3’, and *B. pertussis* BPSM [1] genomic DNA as template. This 634-bp fragment was inserted into Topo PCRII (InVitrogen Life Technology, Groningen, The Netherlands) and then excised as a *Cla*I-*Nhe*I fragment and inserted into *Cla*I- and *Nhe*I-digested pBP23 [2], a suicide vector containing the *E. coli ampG* gene with flanking *B. pertussis* DNA of 618 bp (from position 50,474 to 51,092 of the *B. pertussis* genome) and 379 bp (from position 52,581 to 52,960 of the *B. pertussis* genome) at the 5’ and 3’ end of *E. coli ampG*, respectively. The resulting plasmid was transferred into *E. coli* SM10 [3], which was then conjugated with BPSM, and two successive homologous recombination events were selected as described [4]. Ten individual colonies were screened by PCR as follows. The colonies were suspended in 100 µl H_2O, heated for 20 min. at 95°C, and centrifuged for 5 min at 15,000 x g. One µl of supernatants was then used as template for PCR using oligonucleotides A and C : 5’-TAAGAAGCAAAATAAGCCAGGCATT-3’ to verify the presence of *E. coli ampG* and using oligonucleotides D : 5’-TATACCATGGCGCCGCTGCTGGTGCTGGGC-3’ and E : 5’-TATATCTAGACGCTGGCCGTAACCTTAGCA-3’ to verify the absence of *B. pertussis ampG*. One of the strains containing *E. coli ampG* and lacking *B. pertussis ampG* was then selected, and the entire *ampG* locus was sequenced. This strain was then used for further engineering.

The *ptx* genes were deleted from the chromosome of this strain as described [5] and then replaced by mutated *ptx* coding inactive PTX. The *Eco*RI fragment containing the mutated *ptx* locus from pPT-RE [6] was inserted into the *Eco*RI site of pJQ200mp18rpsl [7]. The resulting plasmid was integrated into the *B. pertussis* chromosome at the *ptx* locus by homologous recombination after conjugation via E. coli SM10. The *ptx* locus in the chromosome of the resulting *B. pertussis* strain was sequenced to confirm the presence of the desired mutations. Toxin production was analyzed by immunoblotting using a mix of monoclonal antibodies IB7 [8] specific for subunit S1, and 11E6 [9] specific for subunits S2 and S3 of PTX.

Finally, the *dnt* gene was deleted from the resulting B. pertussis strain as follows. The *dnt* flanking regions were amplified by PCR using BPSM genomic DNA as template and oligonucleotides F : 5’-TATAGAATTCGCTCGGTTCGCTGGTCAAGG-3’ and G : 5’-TATATCTAGAGCAATGCCGATTCATCTTTA-3’ for the *dnt* upstream region, and H 5’-TATATCTAGAGCGGCCTTTATTGCTTTTCC-3’ and I: 5’-TATAAAGCTTCTCATGCACGCCGGCTTCTC-3’ for the *dnt* downstream region, as primers. The resulting 799-bp and 712-bp DNA fragments were digested with *Eco*RI/*Xba*I and *Xba*I/*Hind*III, respectively, and linked together using the Fast Link kit (Epicentre Biotechnologies, Madison, WI). The ligated fragment was amplified by PCR using oligonucleotides F and I, and the 1505-bp PCR fragment was then inserted into pCR2.1-Topo (Invitrogen), re-isolated from the resulting plasmid as an *Eco*RI fragment and inserted into the unique *Eco*RI site of pJQmp200rpsL18. The resulting plasmid was introduced into *B. pertussis* by conjugation via *E. coli* SM10. Successful deletion of the *dnt* gene by allelic exchange was verified by Southern blot analysis on *Pvu*II-digested *B. pertussis*

genomic DNA using the PCR fragment corresponding to the *dnt* upstream region as a probe. The probe was labeled with digoxigenin (DIG) using the DIG Easy Hyb labeling kit (Roche, Meylan, France). The sizes of the hybridizing bands were determined from the migration distance of the Dig-labeled DNA molecular marker III (Roche). The *dnt* locus of this final strain, named BPZE1 was sequenced.

2. Uppgifter om den färdiga genetiskt modifierade organismen:

a) Beskrivning av genetiska eller fenotypiska egenskaper, särskilt sådana nya egenskaper som kan yttra sig eller inte längre yttrar sig.

The double amino acid mutation of the pertussis toxin results in an over 90% decrease of the enzymatic activity.

The substitution of the *ampG* gene results in a more efficient recycling of degradation products of the peptidoglycans by the *E. coli* transport protein in the attenuated BPZE1 strain and reduces the pathogenicity of the BPZE1 strain. The residual environment (medium) is less than 5% compared to this of the wild type *B. pertussis* strain.

The *dnt* gene is deleted and there is no protein expression of this toxin in the BPZE1 strain.

b) Struktur hos och mängd av den nukleinsyra från vektor eller givare som finns kvar i den modifierade organismens slutliga konstruktion.

The GMO contains the whole *B. pertussis* genome with the exception for the *dnt* and *ampG* genes and in addition the *ampG* gene from *E. coli.*

c) Organismens genetiska stabilitet.

The genetic stability of the BPZE1 has been confirmed after 20 in vitro passages and also after 9 in vivo passages in mice (Feunou et al Vaccine 26 (2008), 5722-5727) and all the genes present before passaging were still present after the in vitro and in vivo passages.

d) Halt av och uttrycksnivå för det nya genetiska materialet samt mätmetoden och dess känslighet.

Reduced amount of mutated pertussis toxin is produced by the GMO strain compared to the production of native pertussis toxin by virulent B. pertussis strain. The level of mutated pertussis toxin produced by the GMO has been evaluated by immunoblot assay. The expression of AmpG has been determined indirectly through the reduction (>95%) of tracheal cytotoxin measured by HPLC in culture supernatant of the GMO strain.

e) De uttryckta proteinernas aktivitet.

The double amino acid mutation of the pertussis toxin results in an over 90% decrease of the enzymatic activity.

The substitution of the *ampG* gene results in a more efficient recycling of degradation products of the peptidoglycans by the *E. coli* transport protein in the attenuated BPZE1 strain and reduces the pathogenicity of the BPZE1 strain. The residual environment (medium) is less than 5% compared to this of the wild type *B. pertussis* strain. The decrease in free TCT results in a decrease in pathogenic effects like mitochondrial bloating, disruption of the tight junctions and extrusion of ciliated cells. The latter TCT-dependent effect is accompanied by a decrease in inflammation and reduction in nitric oxide production.

f) Beskrivning av identifierings- och detekteringsmetoder, inklusive metoder för att identifiera och detektera införd sekvens och vektor.

GMO is identified by genetic characterization using PCR and sequencing techniques.

PCR analysis of the *ampG* and *dnt* loci of BPZE1: Genomic DNA is extracted from isolated colonies and used as template for the PCR using appropriate sense and anti-sense oligonucleotides. The amplified products were analyzed by electrophoresis within a 1% agarose gel in TAE buffer containing ethidium bromide and vizualized by UV light.

Sequence analysis of the *ptx* locus of BPZE1: the DNA fragments containing the region encompassing the R9K and the E129G mutations of the *ptxS1* gene are amplified by PCR from bacterial genomic DNA, using appropriate primers. The amplified DNA fragments are directly sequenced in both directions by automated sequencing.

g) Detekterings- och identifieringsmetodernas känslighet, tillförlitlighet (i kvantitativa termer) och specificitet.

The methods used for GMO detection/identification are based on genetic characterization using PCR and sequencing techniques which are 100% specific, sensitive and reliable.

h) Historik över tidigare utsättningar eller användningar av aktuell genetiskt modifierad organism.

BPZE1 has not been deliberately released before.

i) Hänsyn till människors och djurs hälsa samt växtskydd:

i) Toxiska eller allergiframkallande effekter av genetiskt modifierade organismer eller deras metaboliska produkter.

The GMO is strongly attenuated and does not induce airway inflammation and in fact protects against airway inflammation induced by allergens or viral infections as demonstrated in animal models.

ii) Jämförelse mellan den modifierade organismen och givaren, mottagaren eller (i förekommande fall) moderorganismen avseende patogenicitet.

B. pertussis colonization is strictly limited to respiratory epithelium without extrapulmonary dissemination of the bacteria, which naturally excludes systemic bacteremia of the BPZE1 strain. There is no known animal vector or reservoir for *B. pertussis*, which is predominately a human pathogen although a number of animal models (mice, rats, rabbits and non-human primates) have been described. The genetic modifications (replacement of the ampG gene, deletion of the dermonecrotic toxin and the mutations of the pertussis toxin) are not expected to alter the host range of *B. pertussis* BPZE1 compared to the wild type *B. pertussis* bacteria.
The genetic modifications in BPZE1 strongly increase the in vivo and in vitro safety:
- No lethal effect of BPZE1 was observed in mice, even after nasal administration of 10^6 colony forming units (cfu). The histological analysis data showed a decreased colonization and proliferation power of the BPZE1 cells in the trachea and no weight reduction was observed after nasal administration of the BPZE1-strain.
- Dissemination of BPZE1 was not observed in mice with severe immunodeficiency.
- No cell toxicity of the pneumocyte and macrophage cell lines was observed after incubation with the BPZE1-strain (in vitro safety test).
- The double nucleotide mutation in the substrate binding and the active site of the pertussis toxin (PTX) results in a strong reduction of the enzyme activity.
- The replacement of the *B. pertussis ampG* gene by the *E. coli amp G* gene results in an over 95% reduction in release of the tracheal cytotoxin (TCT) in the medium.
- The dermonecrotic toxin (DNT) is not expressed in the BPZE1 strain.
Based on these data the BPZE1 has been classified as a BSL 1 organism by the French authorities (Appendix)
iii) Koloniseringsförmåga.
BPZE1 colonizes the mouse respiratory tract similar to the wild-type strain, but appears to be highly attenuated as evidenced by histopathological studies. The histology of BPZE1-infected mice is similar to that of control mice. The colonization of the upper respiratory tract of humans will be carefully investigated during the phase I clinical trial.
iv) Om organismen är patogen för människor med ett fungerande immunförsvar:
- De sjukdomar som uppkommer samt patogen mekanism inklusive invasiv förmåga och virulens.
Due to the genetic alterations in BPZE1 in particular the strong reduction of TCT and the genetic inactivation of PTX and also supported by the toxicity testing in mice this strain should not be expected to induce inflammation of the upper respiratory tract.
- Grad av smittsamhet.
B. pertussis is spread by aerosol formed by coughing of infected persons. The coughing is induced by the TCT, which is more than 95% reduced in BPZE1. The GMO is not expected to induce coughing therefore the rate of transmission will be highly unlikely.
- Infekterande dos.
Unknown
- Spektrum av värdorganismer, möjliga förändringar.
The genetic modifications of BPZE1 are not expected to alter the host range of *B. pertussis*. The colonization and growth of BPZE1 is very similar to that of the wildtype *B. pertussis* in vitro and in vivo.
- Förmåga att överleva utanför mänsklig värd.
The genetic modifications of BPZE1 are not expected to alter the host range or the stability of *B. pertussis.*
- Förekomst av vektorer eller spridningssätt.
- Biologisk stabilitet.
The biological stability of BPZE1 does not seem to be different from the wild type *B pertussis.* In mice models BPZE is detected up to 28 days. Bacterial shedding in humans will be investigated twice a week during the first two weeks and thereafter as long as detectable.
- Mönster för antibiotikaresistens.
The GMO kept the same pattern of resistance to antibiotics (nalidixic acid and streptomycin) as the parental strain. The GMO is sensitive to erythromycin, the standard therapy for *B. pertussis* infection. Other antibiotics that may be used for therapeutic intervention are azithromycin, clarithromycin and trimethoprim/sulfamethoxazole
- Allergiframkallande egenskaper.
B pertussis has not been shown to be allergenic in any preclinical or clinical studies to date. On the contrary the BPZE1 has been demonstrated to protect against airway inflammations induced by allergens in a mouse model.
- Befintliga lämpliga behandlingsmetoder.
The BPZE1 strain is sensitive to the antibiotic erythromycin and this standard therapy can be used in case of an accidental infection.
v) Övriga risker förknippade med produkten.

III. Uppgifter om utsättningsförhållanden och den mottagande miljön

A. Uppgifter om utsättningen

1. Beskrivning av den planerade avsiktliga utsättningen, inklusive dess ändamål och förväntade produkter.

BPZE1 is a live, attenuated intranasal vaccine being investigated for the prevention of whooping cough caused by respiratory *B pertussis* in young infants. This study is being conducted to evaluate the safety and immune response of this vaccine in healthy male volunteers.

2. Planerade utsättningstidpunkter och ett tidsschema för försöket med angivande av utsättningarnas frekvens och varaktighet.

It is anticipated that the study will begin in August 2010 and will be completed by June 2011. First a group of12 healthy males will be vaccinated with a single low dose of (10^3 cfu) BPZE1. All twelve individuals will be vaccinated during one week. Blood samples for safety and immunogenicity analysis will be collected before and after vaccination according to the study protocol. Nasopharyngeal samples will be collected twice weekly during the first two weeks and thereafter weekly for analysis of colonization and bacterial shedding as long as bacteria are detected. After at least 4 weeks a new group of 12 healthy males will be vaccinated once with a higher dose (10^5 cfu) of BPZE1 and followed up and after at least 4 weeks a third group of 12 healthy males will be vaccinated once with the highest dose (10^7 cfu) of bacteria and followed. In parallel a group of 12 healthy males will get placebo.

3. Förberedelser avseende platsen för utsättningen.

The study vaccine will be administered at the Karolinska Trial Alliance phase I/II unit, Karolinska University Hospital and conducted by qualified and trained site personnel. The visits will consist of a clinical evaluation of the study subject, as well as the collection of biological specimens (nasal aspirate, saliva and serum samples) according to the study specific laboratory manual and promptly transported to the laboratory for processing and storage via a secure container.

4. Platsens storlek.

N/A

5. Utsättningsmetoder.

The vaccine will be shipped from Innogenetics in a secure freezer container to the phase I/II unit at Karolinska University Hospital, where it will be kept in a looked -80°C freezer until used. The vaccine will be administered intranasally by two tuberculine syringes, one to each nostril.

6. Den mängd genetiskt modifierade organismer som skall sättas ut.

It is estimated that a total of 72 BPZE1 kits including back up vials will be distributed to the trial site. 12 individuals will get 10^3 live bacteria, 12 individuals will get 10^5 live bacteria and 12 individuals will get 10^7 live bacteria.

7. Störningar på platsen (odlingsslag och -metod, gruvbrytning, konstbevattning eller annan verksamhet).

N/A

8. Arbetarskyddsåtgärder som skall vidtas under utsättningen.

Clinical site staff with the responsibility of administering BPZE1, collection of serum and nasal wash specimens, or the clinical evaluation of study subjects are instructed to follow the World Health Organization (WHO) universal precautions for the prevention of transmission of infectious agents in healthcare settings (WHO Standard Precautions 2006). To avoid accidental exposure actions should be taken to minimize generation of aerosols, since the bacteria is strictly a respiratory tract organism. The staff members should wear gloves and eye-protective glasses during the vaccination. Persons handling the BPZE1 bacteria must wash their hands with a suitable disinfecting soap before touching their skin and eyes.

9. Behandling av platsen efter utsättningen.

Equipment and surfaces will be disinfected according to standard medical procedures. All material used for nasal administration will be put in special containers and destroyed according to procedures for hospital waste. The volunteers will stay at the study center for 6 hours after nasal administration.

10. Planerade metoder för eliminering eller inaktivering av genetiskt modifierade organismer vid försökets slut.

All empty vials will be destroyed at the investigational site according to the standard procedures. Unusable study vaccine, i.e. expired vaccine and vaccine having experienced a cold chain break must not be administered but should be destroyed. Following verification of vaccine accountability all unused and unusable study vaccine will be destroyed at the Karolinska Trial Alliance according to standard procedures for hospital waste..

11. Uppgifter om och resultat av tidigare utsättningar av samma genetiskt modifierade organismer, framför allt utsättningar som genomförts i annan skala och i andra ekosystem.

N/A

B. Uppgifter om miljön (både på utsättningsplatsen och i dess omgivningar)

1. Utsättningsplatsens eller -platsernas geografiska lokalisering med hänvisning till rutsystem på

karta (vid ansökningar enligt del C motsvarar utsättningsplatserna de områden där produkten är tänkt att användas).
N/A
2. Fysiskt eller biologiskt avstånd till människor och andra livsformer av betydelse.
N/A
3. Avstånd till betydelsefulla biotoper, skyddade områden eller dricksvattentag.
N/A
4. Klimatförhållanden inom de regioner som kan komma att påverkas.
N/A
5. Geografiska, geologiska och pedologiska förhållanden.
N/A
6. Flora och fauna, inklusive grödor, boskap och migrerande arter.
N/A
7. Beskrivning av målekosystem och andra ekosystem som kan komma att påverkas.
N/A
8. En jämförelse mellan mottagarorganismens naturliga livsmiljö och tänkta utsättningsplatser.
N/A
9. Känd planerad utveckling eller förändring av markanvändningen i regionen som skulle kunna påverka utsättningens miljöpåverkan.
N/A

IV. Uppgifter om interaktion mellan genetiskt modifierade organismer och miljön

A. Egenskaper som påverkar överlevnad, förökning och utbredning
1. Biologiska egenskaper som påverkar överlevnad, förökning och spridning.
None
2. Kända eller förutsedda miljöförhållanden, som kan påverka överlevnad, förökning och utbredning (vind, vatten, mark, temperatur, pH osv.).
N/A
3. Känslighet för specifika former av påverkan.
N/A
B. Interaktion med miljön
1. Förutsedd livsmiljö för de aktuella genetiskt modifierade organismerna.
N/A
2. Undersökningar av uppträdande och egenskaper hos de genetiskt modifierade organismerna samt av ekologiska effekter, utförda i simulerade naturliga miljöer, såsom mikrokosmer, växtkammare eller växthus.
N/A
3. Genöverföringsförmåga:
a) Överföring av genetiskt material från genetiskt modifierade organismer till organismer i påverkade ekosystem efter utsättningen.
The potential for exchange of genetic material is virtually inexistent, since *B. pertussis* does not harbour plasmids or conjugative transposons. In addition B pertussis Tahoma strain (background used for the BPZE1 (GMO) does not harbour intact prophage genomes and is therefore incapable of producing functional phage particles.
b) Överföring av genetiskt material från naturligt förekommande organismer till genetiskt modifierade organismer efter utsättningen.
See above
4. Sannolikheten för att en selektion efter utsättningen leder till att oväntade eller oönskade egenskaper yttrar sig i den modifierade organismen.
N/A
5. Åtgärder för att säkerställa och verifiera genetisk stabilitet. Beskrivning av genetiska egenskaper, som kan hindra eller begränsa spridning av genetiskt material. Metoder för att verifiera genetisk stabilitet.
N/A
6. Biologiska spridningsvägar samt känd eller potentiell interaktion med det som sprids, t.ex. inandning, förtäring, ytkontakt, inträngning osv.
The nasal administration will be performed under strictly controlled conditions at the Karolinska Trial Alliance phase I/II unit.
7. Beskrivning av ekosystem till vilka spridning av de genetiskt modifierade organismerna skulle kunna ske.
N/A
8. Potential för extraordinär populationsökning i miljön.

N/A

9. Konkurrensfördelar för de genetiskt modifierade organismerna i förhållande till icke-modifierade mottagar- eller moderorganismer.

There is no advantage of the GMO compared to wild-type *B. pertussis*. But in mouse model it has been shown that already 3 hours after nasal inoculation of BPZE1 it protects against infection with wild-type *B. pertussis.*

10. Identifiering och beskrivning av målorganismerna (i tillämpliga fall).

N/A

11. Förväntat förlopp och resultat av interaktionen mellan genetiskt modifierade organismer som sätts ut och målorganismerna (i tillämpliga fall).

N/A

12. Identifiering och beskrivning av icke-målorganismer som kan påverkas negativt av utsättningen av den genetiskt modifierade organismen, och förväntade förlopp för identifierad negativ interaktion.

N/A

13. Sannolikheten för förskjutningar i biologisk interaktion eller i spektrum av värdorganismer efter utsättningen.

N/A

14. Känd eller förutsedd interaktion med icke-målorganismer i miljön, inklusive konkurrentorganismer, bytesorganismer, värdorganismer, symbionter, predatorer, parasiter och patogener.

N/A

15. Känd eller förutsedd medverkan i biogeokemiska processer.

N/A

16. Annan potentiell interaktion med miljön.

N/A

V. Uppgifter om övervakning, kontroll, avfallshantering och åtgärdsplaner för nödsituationer

A. Övervakningsmetoder

1. Metoder för att spåra genetiskt modifierade organismer och för att övervaka deras effekter.

Molecular identification by PCR and sequencing

2. Övervakningsmetodernas specificitet (för att identifiera genetiskt modifierade organismer och för att skilja dem från givar-, mottagar- eller i förekommande fall moderorganismerna), känslighet och tillförlitlighet.

The methods used to discriminate GMO and wild-type B. pertussis are based on genetic characterization using PCR and sequencing techniques which are 100% specific, sensitive and reliable.

3. Metoder för att upptäcka överföring av det tillförda genetiska materialet till andra organismer.

N/A

4. Övervakningens varaktighet och frekvens.

N/A

B. Kontroll av utsättningen

1. Metoder och förfaranden för att undvika eller begränsa spridning av genetiskt modifierade organismer bortom utsättningsplatsen eller det avsedda området.

Standard Operating Procedures are available how to store, transport and administer the GMO BPZE1. The procedures are similar to those used for work with infectious material in health care settings.

2. Metoder och förfaranden för att skydda platsen mot tillträde av obehöriga.

The Karolinska Trial Alliance, Karolinska University Hospital is a unit for phase I/II clinical trials with strict rules for access. Only study staff has access to the rooms where the vaccine is stored. The volunteers participating in the study are accompanied by the study staff.

3. Metoder och förfaranden för att hindra att andra organismer tränger in på platsen.

N/A

C. Avfallshantering

1. Typ av avfall som uppstår.

Plastic cryo tubes, syringes, gloves, disposable aprons

2. Förutsedd avfallsmängd.

Less than 5000 cc

3. Beskrivning av planerad avfallshantering.

Destruction according to procedures meant for hospital waste.

D. Åtgärdsplaner i nödsituationer

1. Metoder och förfaranden för att kontrollera de genetiskt modifierade organismerna vid oväntad spridning.

Unexpected spread resulting from accidental spills during any of the administration procedures will be cleaned using 70% alcohol and absorbent material. All material used during the cleaning procedures will be destructed according to procedures meant for hospital waste.
In case of highly unlikely transmission to other humans, accidentally exposed, an efficient treatment against *B. pertussis* is commercially available and is based on administration of erythromycin. BPZE1 has been shown to be sensitive to erythromycin.
2. Metoder för dekontaminering av påverkade områden, t.ex. utrotning av de aktuella genetiskt modifierade organismerna.
N/A
3. Metoder för omhändertagande eller sanering av växter, djur, jord osv., som exponerats i samband med eller efter spridningen.
N/A
4. Metoder för isolering av det område som påverkats av spridningen.
N/A
5. Åtgärdsplaner för att skydda människors hälsa och miljön om oönskade effekter uppträder.
N/A

Ytterligare uppgifter

1. *Produktens föreslagna handelsbeteckning och namn på de genetiskt modifierade organismer som den innehåller, samt eventuella specifika kännetecken, namn eller koder som används av sökanden för att identifiera den genetiskt modifierade organismen. Sedan tillstånd har getts skall uppgift om eventuella nya handelsbeteckningar lämnas till den behöriga myndigheten.*
The product will be called BPZE1
2. Namn och fullständig adress för den person som är etablerad i gemenskapen och som svarar för utsläppandet på marknaden, antingen detta är tillverkaren, importören eller distributören.
Commercialization is not planned yet.
Dr. Camille Locht, Inserm – Institut Pasteur de Lille, 1, rue du Prof. Calmette, F-59019 Lille Cedex, France; Phone + (0) 3 20 87 11 51; Fax + (0) 3 20 87 11 58; e-mail : camille.locht@pasteur-lille.fr
3. *Namn och fullständig adress för den eller de personer som lämnar kontrollprover.*
N/A
4. Beskrivning av hur produkten, och den genetiskt modifierade organism som utgör eller ingår i en produkt, är avsedd att användas. Skillnader i användningen eller hanteringen av den genetiskt modifierade organismen jämfört med liknande produkter som inte är genetiskt modifierade bör belysas.
BPZE1, a live attenuated *B. pertussis*, will be given as a single intranasal dose to healthy male volunteers in a phase I, single centre, dose-escalating, placebo-controlled study. The proposed live BPZE1 is expected to induce protection against whooping cough caused by the wild-type *B. pertussis*.
5. Beskrivning av det geografiska område eller de geografiska områden och miljötyper där produkten är avsedd att användas inom gemenskapen inbegripet, om det är möjligt, en beräkning av användningens omfattning i varje område.
N/A
6. Tänkta kategorier av användare t.ex. industri, jordbruk och övriga yrkesutövare, konsumenter i allmänhet.
At present for research use only in hospital settings; in future hopefully for common use in national child immunization programs.
7. Uppgifter om den genetiska modifieringen. Uppgifterna bör i förekommande fall innehålla upplysningar om deponeringen av prover av den genetiskt modifierade organismen eller dess genetiska material hos den behöriga myndigheten samt upplysningar om nukleotidsekvenser och övriga typer av uppgifter som krävs för att identifiera en produkt som innehåller genetiskt modifierade organismer och dess resultat. Med sistnämnda uppgifter avses exempelvis en metod för att upptäcka och identifiera den produkt som innehåller genetiskt modifierade organismer, inbegripet uppgifter om experiment som visar metodens särskilda egenskaper. Konfidentiella uppgifter, som inte kan införas i den offentliga delen av registret, skall identifieras.
Dessa uppgifter skall lämnas för att göra det möjligt att föra in modifieringar av organismer i ett eller flera register. Registren har till syfte att underlätta kontrollen och övervakningen av produkter efter det att de släppts ut på marknaden, och skall användas för att spåra och identifiera särskilda produkter som innehåller genetiskt modifierade organismer.
Information will be available in the application to the Medical Product Agency, Sweden.
8. Föreslagen märkning på en etikett eller i ett följedokument. Denna skall innehålla, åtminstone i komprimerad form, en handelsbeteckning för produkten, en förklaring att "denna produkt innehåller genetiskt modifierade organismer", den genetiskt modifierade organismens namn och de uppgifter som avses i punkt 2. Märkningen bör ange hur uppgifter kan erhållas i den offentliga delen av registret.

Information about the labelling will be available in the application to the Medical Product Agency, Sweden.
B. Utöver vad som anges i punkt A skall, i enlighet med 3 kap. 4 § förordningen (2002:1086) om utsättning av genetiskt modifierade organismer i miljön, följande uppgifter lämnas i ansökan om tillstånd när det är relevant
1. Åtgärder som skall vidtas vid oavsiktlig utsättning eller missbruk.
N/A
2. Särskilda instruktioner eller rekommendationer för lagring och hantering.
Standard Operating Procedures for transport, storage, administration and destruction will be available at the Karolinska Trial Alliance, Karolinska University Hospital.
3. Särskilda instruktioner till sökanden för att denne skall kunna genomföra övervakning och rapportering. Om det behövs skall sådana instruktioner även rikta sig till behörig tillsynsmyndighet, så att denna kan få nödvändig information om eventuella negativa effekter. Dessa instruktioner bör stämma överens med bilaga 4 del C.
4. Föreslagna restriktioner för användningen av den genetiskt modifierade organismen, exempelvis uppgifter om var produkten får användas och för vilket ändamål.
5. Föreslagen förpackning.
6. Beräknad produktion i eller import till gemenskapen.
7. Föreslagen ytterligare märkning. Detta kan innefatta, åtminstone i komprimerad form, de uppgifter som avses i punkterna A4, A5, B1, B2, B3 och B4.

Övervakningsplan
N/A

APPENDIX 2

XVIII.2. OFFICIAL FRENCH AGREEMENT ON THE DOWNGRADING OF BPZE1 AS A CLASS I ORGANISM

Liberté • Égalité • Fraternité
RÉPUBLIQUE FRANÇAISE

MINISTERE
DE L'ENSEIGNEMENT SUPERIEUR
ET DE LA RECHERCHE

Direction générale pour la recherche et l'innovation

04 JUIN 2009

DECISION D'AGREMENT

La Ministre de l'Enseignement Supérieur et de la Recherche,

Vu le décret n° 93-773 du 27 mars 1993 pris pour l'application s'agissant des utilisations civiles de l'article 6 de la loi n° 92-654 du 13 juillet 1992 relative au contrôle de l'utilisation et de la dissémination des organismes génétiquement modifiés et modifiant la loi n° 76-663 du 19 juillet 1976 relative aux installations classées pour la protection de l'environnement, et notamment ses articles 20 et 21 ainsi que l'arrêté du 27 décembre 1994 relatif au dossier de demande d'agrément prévu au titre Ier dudit décret.

Vu le décret n° 93-774 du 27 mars 1993 fixant la liste des techniques de modification génétique et les critères de classement des organismes génétiquement modifiés.

Vu l'arrêté du 27 décembre 1994 relatif au dossier de demande d'agrément prévu au titre Ier du décret n°93-773 du 27 mars susmentionné.

Vu le complément de dossier à la demande d'agrément enregistrée sous les n^{os} **4279/4280,** déposé par l'**INSERM**, exploitant représenté par Madame **Christine MAZINGUE** et le directeur scientifique du projet, Madame **Camille LOCHT**, conformément à l'arrêté du 27 décembre 1994, et enregistré sous le n° **4279/4280 CA-II.**

Vu l'avis de la Commission de génie génétique en date du **09 avril 2009.**

Décide :

Article 1 : l'agrément de groupe II est accordé à l'utilisation comprenant les projets suivants pour une durée maximale de **5 ans** , à compter de la décision princeps d'agrément pour les dossiers n^{os} **4279/4280** en date du **11 avril 2005.**

1, rue Descartes – 75231 PARIS Cedex 05 – Tél. : 01.55.55.98.09 – Fax : 01.55.55.99.97
http://wwww.enseignementsup-recherche.gouv.fr/commis/genetique/index.htm

APPENDIX 3

XVIII.3. DECLARATION OF HELSINKI

WORLD MEDICAL ASSOCIATION DECLARATION OF HELSINKI

Ethical Principles for Medical Research Involving Human Subjects

Adopted by the 18th WMA General Assembly, Helsinki, Finland, June 1964, and amended by the:
29th WMA General Assembly, Tokyo, Japan, October 1975
35th WMA General Assembly, Venice, Italy, October 1983
41st WMA General Assembly, Hong Kong, September 1989
48th WMA General Assembly, Somerset West, Republic of South Africa, October 1996
52nd WMA General Assembly, Edinburgh, Scotland, October 2000
53th WMA General Assembly, Washington 2002 (Note of Clarification on paragraph 29 added)
55th WMA General Assembly, Tokyo 2004 (Note of Clarification on Paragraph 30 added)
59th WMA General Assembly, Seoul, October 2008

A. INTRODUCTION

1. The World Medical Association (WMA) has developed the Declaration of Helsinki as a statement of ethical principles for medical research involving human subjects, including research on identifiable human material and data.

The Declaration is intended to be read as a whole and each of its constituent paragraphs should not be applied without consideration of all other relevant paragraphs.

2. Although the Declaration is addressed primarily to physicians, the WMA encourages other participants in medical research involving human subjects to adopt these principles.

3. It is the duty of the physician to promote and safeguard the health of patients, including those who are involved in medical research. The physician's knowledge and conscience are dedicated to the fulfilment of this duty.

4. The Declaration of Geneva of the WMA binds the physician with the words, “The health of my patient will be my first consideration,” and the International Code of Medical Ethics declares that, “A physician shall act in the patient's best interest when providing medical care.”

5. Medical progress is based on research that ultimately must include studies involving human subjects. Populations that are underrepresented in medical research should be provided appropriate access to participation in research.

6. In medical research involving human subjects, the well-being of the individual research subject must take precedence over all other interests.

7. The primary purpose of medical research involving human subjects is to understand the causes, development and effects of diseases and improve preventive, diagnostic and therapeutic interventions (methods, procedures and treatments). Even the best current interventions must be evaluated continually through research for their safety, effectiveness, efficiency, accessibility and quality.

8. In medical practice and in medical research, most interventions involve risks and burdens.

9. Medical research is subject to ethical standards that promote respect for all human subjects and protect their health and rights. Some research populations are particularly vulnerable and need special protection. These include those who cannot give or refuse consent for

themselves and those who may be vulnerable to coercion or undue influence.

10. Physicians should consider the ethical, legal and regulatory norms and standards for research involving human subjects in their own countries as well as applicable international norms and standards. No national or international ethical, legal or regulatory requirement should reduce or eliminate any of the protections for research subjects set forth in this Declaration.

B. PRINCIPLES FOR ALL MEDICAL RESEARCH

11. It is the duty of physicians who participate in medical research to protect the life, health, dignity, integrity, right to self-determination, privacy, and confidentiality of personal information of research subjects.

12. Medical research involving human subjects must conform to generally accepted scientific principles, be based on a thorough knowledge of the scientific literature, other relevant sources of information, and adequate laboratory and, as appropriate, animal experimentation. The welfare of animals used for research must be respected.

13. Appropriate caution must be exercised in the conduct of medical research that may harm the environment.

14. The design and performance of each research study involving human subjects must be clearly described in a research protocol. The protocol should contain a statement of the ethical considerations involved and should indicate how the principles in this Declaration have been addressed. The protocol should include information regarding funding, sponsors, institutional affiliations, other potential conflicts of interest, incentives for subjects and provisions for treating and/or compensating subjects who are harmed as a consequence of participation in the research study. The protocol should describe arrangements for post-study access by study subjects to interventions identified as beneficial in the study or access to other appropriate care or benefits.

15. The research protocol must be submitted for consideration, comment, guidance and approval to a research ethics committee before the study begins. This committee must be independent of the researcher, the sponsor and any other undue influence. It must take into consideration the laws and regulations of the country or countries in which the research is to be performed as well as applicable international norms and standards but these must not be allowed to reduce or eliminate any of the protections for research subjects set forth in this Declaration. The committee must have the right to monitor ongoing studies. The researcher must provide monitoring information to the committee, especially information about any serious adverse events. No change to the protocol may be made without consideration and approval by the committee.

16. Medical research involving human subjects must be conducted only by individuals with the appropriate scientific training and qualifications. Research on patients or healthy volunteers requires the supervision of a competent and appropriately qualified physician or other health care professional. The responsibility for the protection of research subjects must always rest with the physician or other health care professional and never the research subjects, even though they have given consent.

17. Medical research involving a disadvantaged or vulnerable population or community is only justified if the research is responsive to the health needs and priorities of this population or community and if there is a reasonable likelihood that this population or community stands to benefit from the results of the research.

18. Every medical research study involving human subjects must be preceded by careful assessment of predictable risks and burdens to the individuals and communities involved in the research in comparison with foreseeable benefits to them and to other individuals or

communities affected by the condition under investigation.

19. Every clinical trial must be registered in a publicly accessible database before recruitment of the first subject.

20. Physicians may not participate in a research study involving human subjects unless they are confident that the risks involved have been adequately assessed and can be satisfactorily managed. Physicians must immediately stop a study when the risks are found to outweigh the potential benefits or when there is conclusive proof of positive and beneficial results.

21. Medical research involving human subjects may only be conducted if the importance of the objective outweighs the inherent risks and burdens to the research subjects.

22. Participation by competent individuals as subjects in medical research must be voluntary. Although it may be appropriate to consult family members or community leaders, no competent individual may be enrolled in a research study unless he or she freely agrees.

23. Every precaution must be taken to protect the privacy of research subjects and the confidentiality of their personal information and to minimize the impact of the study on their physical, mental and social integrity.

24. In medical research involving competent human subjects, each potential subject must be adequately informed of the aims, methods, sources of funding, any possible conflicts of interest, institutional affiliations of the researcher, the anticipated benefits and potential risks of the study and the discomfort it may entail, and any other relevant aspects of the study. The potential subject must be informed of the right to refuse to participate in the study or to withdraw consent to participate at any time without reprisal. Special attention should be given to the specific information needs of individual potential subjects as well as to the methods used to deliver the information. After ensuring that the potential subject has understood the information, the physician or another appropriately qualified individual must then seek the potential subject's freely-given informed consent, preferably in writing. If the consent cannot be expressed in writing, the non-written consent must be formally documented and witnessed.

25. For medical research using identifiable human material or data, physicians must normally seek consent for the collection, analysis, storage and/or reuse. There may be situations where consent would be impossible or impractical to obtain for such research or would pose a threat to the validity of the research. In such situations the research may be done only after consideration and approval of a research ethics committee.

26. When seeking informed consent for participation in a research study the physician should be particularly cautious if the potential subject is in a dependent relationship with the physician or may consent under duress. In such situations the informed consent should be sought by an appropriately qualified individual who is completely independent of this relationship.

27. For a potential research subject who is incompetent, the physician must seek informed consent from the legally authorized representative. These individuals must not be included in a research study that has no likelihood of benefit for them unless it is intended to promote the health of the population represented by the potential subject, the research cannot instead be performed with competent persons, and the research entails only minimal risk and minimal burden.

28. When a potential research subject who is deemed incompetent is able to give assent to decisions about participation in research, the physician must seek that assent in addition to the consent of the legally authorized representative. The potential subject's dissent should be respected.

29. Research involving subjects who are physically or mentally incapable of giving consent, for

example, unconscious patients, may be done only if the physical or mental condition that prevents giving informed consent is a necessary characteristic of the research population. In such circumstances the physician should seek informed consent from the legally authorized representative. If no such representative is available and if the research cannot be delayed, the study may proceed without informed consent provided that the specific reasons for involving subjects with a condition that renders them unable to give informed consent have been stated in the research protocol and the study has been approved by a research ethics committee. Consent to remain in the research should be obtained as soon as possible from the subject or a legally authorized representative.

30. Authors, editors and publishers all have ethical obligations with regard to the publication of the results of research. Authors have a duty to make publicly available the results of their research on human subjects and are accountable for the completeness and accuracy of their reports. They should adhere to accepted guidelines for ethical reporting. Negative and inconclusive as well as positive results should be published or otherwise made publicly available. Sources of funding, institutional affiliations and conflicts of interest should be declared in the publication. Reports of research not in accordance with the principles of this Declaration should not be accepted for publication.

C. ADDITIONAL PRINCIPLES FOR MEDICAL RESEARCH COMBINED WITH MEDICAL CARE

31. The physician may combine medical research with medical care only to the extent that the research is justified by its potential preventive, diagnostic or therapeutic value and if the physician has good reason to believe that participation in the research study will not adversely affect the health of the patients who serve as research subjects.

32. The benefits, risks, burdens and effectiveness of a new intervention must be tested against those of the best current proven intervention, except in the following circumstances:

• The use of placebo, or no treatment, is acceptable in studies where no current proven intervention exists; or

• Where for compelling and scientifically sound methodological reasons the use of placebo is necessary to determine the efficacy or safety of an intervention and the patients who receive placebo or no treatment will not be subject to any risk of serious or irreversible harm. Extreme care must be taken to avoid abuse of this option.

33. At the conclusion of the study, patients entered into the study are entitled to be informed about the outcome of the study and to share any benefits that result from it, for example, access to interventions identified as beneficial in the study or to other appropriate care or benefits.

34. The physician must fully inform the patient which aspects of the care are related to the research. The refusal of a patient to participate in a study or the patient’s decision to withdraw from the study must never interfere with the patient-physician relationship.

35. In the treatment of a patient, where proven interventions do not exist or have been ineffective, the physician, after seeking expert advice, with informed consent from the patient or a legally authorized representative, may use an unproven intervention if in the physician's judgement it offers hope of saving life, re-establishing health or alleviating suffering. Where possible, this intervention should be made the object of research, designed to evaluate its safety and efficacy. In all cases, new information should be recorded and, where appropriate, made publicly available.

APPENDIX 4

XVIII.4. GUIDANCE FOR INDUSTRY. TOXCICITY GRADING SCALE FOR HEALTHY ADULT AND ADOLESCENT VOLUNTEERS IN PREVENTIVE VACCINE CLINICAL TRIALS
