# Supplementary material for: A Phase I Clinical Study of a Live Attenuated Bordetella pertussis Vaccine - BPZE1; A Single Centre, Double-Blind, Placebo-Controlled, Dose-Escalating Study of BPZE1 Given Intranasally to Healthy Adult Male Volunteers
Source: PLoS One. 2014 Jan 8;9(1):e83449. doi: 10.1371/journal.pone.0083449 (PMC3885431; doi:10.1371/journal.pone.0083449)
Supplement: Method S1 — Inclusion and Exclusion Criteria. (DOCX) [file pone.0083449.s007.docx]

**Method S1. INCLUSION AND EXCLUSION CRITERIA**

**INCLUSION CRITERIA**

Subjects meeting all the following criteria will be eligible for inclusion:

- Healthy male born between 1979 and 1991 who has not experienced clinical pertussis (lab. verified) during the past 10 years and who has not been vaccinated with any pertussis vaccine.
- Informed consent form signed by the subject.
- Subject shall be able to attend all scheduled visits and to understand and comply with the study procedures (e.g. able to read and write Swedish).

**EXCLUSION CRITERIA**

Subjects meeting at least one of the following criteria were ineligible for inclusion:

- Pertussis toxin serum IgG antibodies ≥20 international units/ml.
- Blood pressure after resting ≥ 150/90 mm Hg.
- Heart rate after resting ≥ 80 bpm.
- Respiratory rate after resting ≥ 20 per minute minute.
- Unwillingness to refrain from the use of nicotine products from screening through day 28.
- Use of narcotic drugs and/or a history of drug/alcohol abuse whitin the past 2 years prior to screening
- Donation of blood or suffering from blood loss of ≥ 450 ml within 60 days prior to screening or donation of plasma within 14 days prior to screening.
- Receipt of immunoglobulin, blood derived products, systemic corticosteroids or other immunosuppressant drugs within 90 days prior to day 0.
- Use of corticosteroids in the respiratory tract (e.g. nasal steroids, inhaled steroids) 30 days prior to day 0.
- Use of herbal medications or dietary supplements within 7 days prior to day 0 at the discretion of the investigator. Unwillingness to refrain from herbal medications or dietary supplements within 30 days after day 0 at the discretion of the investigator.
- Receipt of a vaccine within the last 30 days prior to day 0 or planned vaccination within 30 days after day 0.
- Evolving encephalopathy not attributable to another identifiable cause within 7 days of administration of a previous dose of any vaccine.
- Known hypersensitivity to any component of the study vaccine.
- Current participation in any other clinical trial or participation in any clinical trial in the previous 3 months prior to day 0.
- Inability to adhere to the protocol, including plans to move from the area.
- Family history (first degree) of congenital or hereditary immunodeficiency.
- Infection with HIV, hepatitis B or C.
- Any medical condition which, in the opinion of the investigator, might interfere with the evaluation of the study objectives.
- Clinically significant abnormal laboratory values at the discretion of the investigator.
- Frequent contact with children less than 1 year of age (father, childcare worker, nurse, etc…) or residence in the same household as persons with known immunodeficiency including persons on immunosuppressive treatment.
