# Supplementary material for: A Phase I Clinical Study of a Live Attenuated Bordetella pertussis Vaccine - BPZE1; A Single Centre, Double-Blind, Placebo-Controlled, Dose-Escalating Study of BPZE1 Given Intranasally to Healthy Adult Male Volunteers
Source: PLoS One. 2014 Jan 8;9(1):e83449. doi: 10.1371/journal.pone.0083449 (PMC3885431; doi:10.1371/journal.pone.0083449)
Supplement: Method S2 — Nasopharyngeal Aspirate Cultivation. (DOCX) [file pone.0083449.s008.docx]

**Method S2. NASOPHARYNGEAL ASPIRATE CULTIVATION**

**Purpose**

To describe workflow in the cultivation of nasopharyngeal aspirates in the study BT0604, also known as Child Innovac 1.

**Materials**

1. Test tube rack (large and small)
2. White loop (1μL)
3. Blue loop (10μL)
4. Sterile forceps
5. Culture medium, Charcoal plates, ordered from the Karolinska University Laboratory, Substrates Division Solna, Sweden
6. Swabs with rayon wool
7. Plastic container with screw cap, for waste
8. Absorbent towels
9. Gloves
10. 70% ethanol / surface disinfection DES +45
11. Labels with sample id
12. Form of receipt of sample
13. Form of readouts
14. Slides
15. Cryotubes containing 0.5 ml of freezing medium
16. Sarstedt tubes art.no. 72,694,005
17. Storage Boxes

**Prescription**

Medium for cultivation
Containment and transport medium according to Regan and Lowe (J Clin Microbiol 1977, 6:303-309).

*Recipe for 1 L*

Charcoal agar (Oxoid, CM119) with 10% horse blood and cephalexin 40 mg / L Solve content below (51 g) in 1000 ml of distilled water *Content Grams / L*

- LabLemco powder 10.0
- Peptone, 10.0
- Starch, 10.0
- Bacteriologically carbon, 4.0
- NaCl, 5.0
- Nicotinic acid, 0.001
- Agar, 12.0

Autoclave (121˚ C for 15 minutes) and allow to cool to 50 ˚ C
Add 10% defibrinated horse blood and mix gently
Add cephalexin (final conc 40 mg / L) 40 mg
pH should be 7.4 ± 0.2 at 25˚ C

Use approximately 30 ml of medium for each plate. The plates are stored at 4-8˚ C. They can be stored in a plastic bag in the refrigerator up to 4 weeks. If this deadline is exceeded, there is a risk of reduced isolation frequency. Each batch of agar should be checked by culturing the control strains.

Each batch of plates is tested with the following control strains:

- Bordetella pertussis CCUG 35196
- Bordetella parapertussis ATCC 15237
- BPZE1 GM vaccine strain from Inserm.

Transport medium/ enrichment medium

The transport medium has the same content as the plates although they have half of the agar concentration. Add 10 % defibrinated lysed horse blood. 2.5-4 ml medium is filled in a tube with a screw-on cap (Sarstedt art nr 60.542.024). Store the tubes in the same way as the plates. The transport medium can be ordered from: Karolinska Universitetslaboratoriet, Substratenheten Solna, Sweden.

Freezing Medium

Bovine serum albumin (BSA)
25% sterile filtered solution in 0.01 M phosphate buffer pH 7.2 to 7.4

Saccharose phosphate glutamate (SPG) with BSA

- Sucrose, 74.6 g
- K-glutamate, 0.91 g
- KH_2_PO_4_, 0.512 g
- K_2_HPO_4_, 1.24 g
- Add distilled water to 1000 ml
- Add 110 ml of sterile filtered BSA as described above, i.e. the final concentration of 2.5%.

Dispense 0.5 ml of SPG + BSA in tubes with freezing medium.

Make a strong inoculum and store at -50˚ C or less.
 **Equipment**

1. Incubator (35 - 37° C)
2. Refrigerator
3. Freezer -50˚ C or less
4. Microscope
5. Vortex

**Working Procedure**

1. The test samples come with courier transportation from KTA Huddinge.
2. Unzip the sample and check sample id against referral slip.
3. Record incoming sample form Child Innovac - Reception of nasopharyngeal aspirates.

*The cultivation of aspirates*

1. Allow the plate to stand at room temperature for 15 min before use.
   Label plate with sample id.
2. Check marking of the plate with the labeling of the aspirates.
3. Homogenize aspirate thoroughly (at least 60 sec) on the Vortex.
4. Transfer with a sterile pipette 100 μL homogenized aspirate on a charcoal plate. Spread with a loop.
5. Incubate at 35-37˚ C and high humidity (60-70%). Keep the plates with the lid down to prevent condensation on the agar surface.
6. The plates are read daily for 3-7 days.
7. Note the reading results on form.
8. Label 4 sterile tubes (Sarstedt art.no. 72,694,005) relating to sample id.
9. Transfer 50 μL aspirate into one tube and 200 μL to another tube.
10. Distribute the remaining aspirates into two tubes. Aspirates are kept frozen in -50˚ C or below for later transport to the SMI.

**Enrichment Medium**

1. Incubate transportation / enrichment medium at 35-37˚ C for 72 hours after collection.
2. Inoculate the catheter tip onto a plate (use sterile tweezers).
3. Dip a swab with rayon wool in enrichment media and spread out onto the same plate.
4. Incubate at 35-37˚ C and high humidity (60-70%). Keep the plates with the lid down to prevent condensation on the agar surface.
5. Read the plates on a daily basis for 4 days.
6. Note the readouts on form.

*Note! If there is any growth on day 28 ± 1, contact the Karolinska Trial Alliance.* *They can be notified by telephone but the message must be confirmed by fax or e-mail.*
**Identification of B. Pertussis**

*Suspicious colonies are tested as follows*

- Gram stain (Bordetella is a small gram negative rod)
- Oxidase Test
- Catalase

*Biochemical identification of Bordetella species*

| Test | B.pertussis | B.parapertussis | B.bronchiseptica |
| --- | --- | --- | --- |
| Catalase | + | + | + |
| Oxidase | + | - | - |

**Strains**

Strains are frozen in freezing medium for subsequent molecular typing.

**Safety and environmental aspects**

KUL0016-3, Premises and Environment, Karolinska University Laboratory.

**References**

Efficacy Trial of acellular pertussis vaccine, Technical Report 1995^th^

**Certificate of qualifications**

Certificate of qualifications is issued after individual assessment of competence and is signed by the responsible for the method.

**Routine for replying**

Written response to the growth made on aspirates and enrichment media from day 28 and day 45
Label with "Growth of Bordetella pertussis" or "No growth of Bordetella pertussis" pasted on the referral slip. The response is to be dated and signed.
