# Supplementary material for: A Phase I Clinical Study of a Live Attenuated Bordetella pertussis Vaccine - BPZE1; A Single Centre, Double-Blind, Placebo-Controlled, Dose-Escalating Study of BPZE1 Given Intranasally to Healthy Adult Male Volunteers
Source: PLoS One. 2014 Jan 8;9(1):e83449. doi: 10.1371/journal.pone.0083449 (PMC3885431; doi:10.1371/journal.pone.0083449)
Supplement: Method S3 — Serum IgG Antibody Analyses. (DOCX) [file pone.0083449.s009.docx]

Method S3. SERUM IgG ANTIBODY ANALYSES

ELISA in general

High binding polystyrene plates were coated with antigen. Eight two-fold dilutions of sera were used. A reference line assay was used to calculate arbitrary units (EU) per ml of test samples from the absorbance value (A405) of a calibrator (14). The results were accepted if the controls of the plates were within given limits of antibody concentration. If not the plate was retested. An increase is considered as significant if antibody level is doubled compared to pre-vaccination level and at least 4 times the minimum level of detection (MLD).

Anti-PT

Pertussis toxin (TOH15, SmithKline Beecham) diluted to 1 mg/L in PBS pH 7.4 was used for coating over night at room temperature. A NIBSC reference human antiserum, 06/142, containing 106 IU/ml of IgG anti-PT was used as calibrator. A pool of fractioned human plasma (IgG42) 46.5 IU/ml diluted 1/20 was used as a monitor. The minimum level of detection (MLD) was 1 IU/ml.

Anti-FHA

FHA (TOH 15, Smith-Kline-Beecham) diluted to 1.5 mg/L in PBS pH 7.4 was used for coating over night at room temperature. A NIBSC reference human antiserum, 06/142, containing 122 IU/ml of IgG anti-FHA was used as calibrator. A pool of fractioned human plasma (Ig42) 112 IU/ml diluted 1/20 was used as a monitor. The minimum level of detection (MLD) was 1 IU/ml.

Anti-pertactin

Pertactin (SKA-QCDSCO4420, Aventis Pasteur Canada) diluted to 2.5 mg/L in carbonate buffer pH 9.6 was used for coating over night at room temperature. A NIBSC reference human antiserum, 06/142, containing 39 IU/ml of IgG anti-pertactin was used as calibrator. A pool of fractioned human plasma (IgG42) 14 IU/ml diluted 1/100 was used as a monitor. The minimum level of detection (MLD) was 2 IU/ml.

Anti-fimbriae 2/3

Fimbriae 2+3 (CAG9/14-PDS, Connaught Laboratories Ltd, Ontario Canada) diluted to 1.0 mg/L in PBS pH 7.4 was used for coating over night at room temperature. As there is no international reference human antiserum, a pool of fractioned human plasma (IgG42) assigned the value of 4000 EU/ml IgG anti-fim diluted 1/100 was used as calibrator. A pool of positive sera (pool 2/2000) 37.5 EU/ml was used as a monitor. The minimum level of detection (MLD) was 1 EU/ml.
